# Visible light-driven Giese reaction with alkyl tosylates catalysed by nucleophilic cobalt†

**DOI:** 10.1039/d0ra10739e

**Published:** 2021-01-18

**Authors:** Kimihiro Komeyama, Takuya Michiyuki, Yoshikazu Teshima, Itaru Osaka

**Affiliations:** Department of Applied Chemistry, Graduate School of Advanced Science and Engineering, Hiroshima University 1-4-1 Kagamiyama Higashi-Hiroshima City Hiroshima 739-8527 Japan kkome@hiroshima-u.ac.jp

## Abstract

The scope of the Giese reaction is expanded using readily available alkyl tosylates as substrates and nucleophilic cobalt(i) catalysts under visible-light irradiation. The reaction proceeds preferentially with less bulky primary alkyl tosylates. This unique reactivity enables the regio-selective Giese reaction of polyol derivatives.

## Abstract 2

We report an efficient approach for the Giese reaction with abundant alkyl tosylates as alkyl radical sources using a nucleophilic cobalt catalyst under visible-light irradiation.

## Graphical abstract

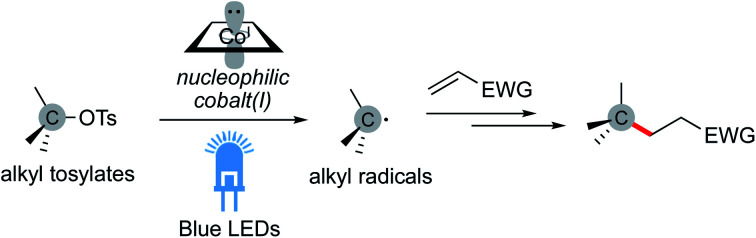

Organic transformations involving alkyl radicals are powerful tools for the construction of C(sp^3^)-containing carbon–carbon bonds.^1^ Particularly, the conjugate radical addition to electron-deficient olefins (Giese reaction) is a versatile protocol for C(sp^3^)–C(sp^3^) bond formation in the synthesis of natural products and pharmaceuticals^2^ because it enables the introduction of alkyl moieties in a site-selective manner.

Typically, the Giese reaction involves the generation of alkyl radicals from organohalides using stannyl radicals,^3^ silyl radicals,^4^ and transition-metal catalysts.^5^ More recently, oxidative protocols have been extensively developed to generate alkyl radicals from alkyl metallic reagents.^6^ However, these approaches are disadvantageous; halogenated waste is generated, and the preparation of most of these alkyl radical sources requires several steps from commercially available chemicals, resulting in a multi-step process to construct the desired carbon–carbon bonds. Therefore, the development of Giese reactions with naturally abundant carbon sources such as alkyl carboxylic acids^7^ and alkyl amines^8^ is receiving increased attention.

Alkanols are also among the most critical carbon sources because they are abundant in bioactive molecules and natural products in the form of sugars, steroids and among others. Furthermore, the hydroxyl substituent plays a vital role as a directing group for C–H bond functionalisations.^9^ Therefore, the development of the Giese reaction from alkanols is of great importance in modern synthetic organic chemistry (Scheme 1).

**Scheme 1:**
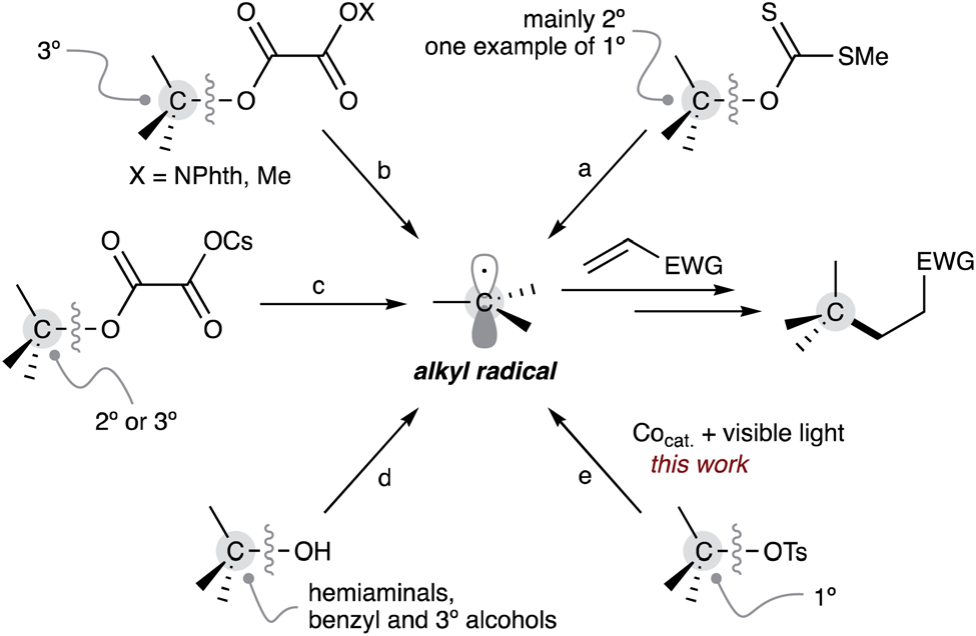
Examples of alcohol-based Giese reaction.

In 2000, Togo reported the first example of a Giese reaction using alkyl xanthates as alkyl radical equivalents (Scheme 1a).^10^ Overman applied a reductive approach for alkyl radical generation from alkyl *N*-phthalimidoyl oxalates by a photocatalyst to the Giese reaction (Scheme 1b).^11^ Similarly, Gong demonstrated the reductive strategy of alkyl methyl oxalates as alternative alkyl radical sources according to a unique reaction design.^12^ Moreover, Overman and MacMillan found a radical addition using alkyl cesium oxalates as the source of alkyl radicals (Scheme 1c).^13^ Recently, a direct approach from alkanols to alkyl radicals using a low-valent titanocene catalyst was also demonstrated (Scheme 1d).^14^

These protocols are pioneering works that allowed using abundant alkanols and their derivatives as alkyl radical sources for the Giese reaction. However, since these approaches involve the homolytic cleavage of the robust alcoholic C–O bond, the efficiency of the alkyl radical formation greatly depends on the thermodynamic stability of the generated alkyl radicals. Therefore, these Giese reactions are typically limited to tertiary, secondary, and benzylic alkyl radicals as well as heteroatom-linked carbon radicals generated from hemiaminals. Access to non-stabilised primary alkyl radicals from primary alcohols and their derivatives is more challenging.

We recently demonstrated various nickel and nucleophilic cobalt(i)-catalysed transformations of alkyl tosylates, including C(sp^3^)–C(sp^2^)^15^ and C(sp^3^)–C(sp^3^) couplings^16^ and amidation.^17^ These reactions start from an S_N_2-type oxidative addition of, especially, primary alkyl tosylates to a nucleophilic cobalt(i) centre, producing an alkyl-cobalt(iii) species that enables a transalkylation with a nickel to form a reactive alkyl nickel intermediate. On the other hand, the resulting alkyl-cobalt(iii) generates an alkyl radical through homolytic Co–C bond cleavage by visible-light irradiation. Numerous radical reactions that involve the catalytic photo-cleavage of a Co–C bond has been developed;^18^ in contrast, the Giese reaction using unactivated alkyl tosylates as alkyl radical sources has never been reported so far. Herein, we report a Giese reaction that uses alkyl tosylates as alkyl radical sources, benefiting from the generation of non-stabilised alkyl radicals by photo-cleavage of the Co–C bond on the corresponding alkyl-cobalt(iii) species (Scheme 1e).

Our investigation started by optimising the reaction of alkyl tosylate 1a with activated olefin 2a (Table 1). When 1a was treated with 2a (1.0 equiv.) in the presence of vitamin B_12_ (VB_12_, 5 mol%) as a catalyst, Mn (3.0 equiv.) as a reductant, and Et_3_N·HCl (1.5 equiv.) as a proton donor under blue-light irradiation (*λ* = 454 nm, 40 W), the expected radical adduct 3a was obtained in 61% yield (Table 1, entry 1). In the reaction, the presence and type of proton donors greatly affected the reaction efficiency (entries 2–4). The choice of the planar-cobalt catalysts was also crucial. Thus, CoCl(dmgH)_2_Py, Co(salen), and Co(Pc) catalysts gave the product in negligible yields (entries 5–7), whereas Co(TMPP) afforded the product in 58% yield (entry 8). The present standard conditions gave 3a in 15% yield, even under ambient conditions without the light (entry 9). In contrast, the reaction rarely occurred in complete darkness (entry 10). Furthermore, a periodic light-ON/OFF switching experiment in the cobalt-catalysed reaction of 1a with 2a (Fig. 1) and time-dependent UV-Vis absorption spectra of methylcobalamin under blue-light irradiation (Fig. 2) indicated that the photo-induced homolytic cleavage of the Co–C bond on the alkyl-Co(iii) intermediate produced a Co(ii) complex and an alkyl radical.^19^ A slight reduction of the yield was obtained using Zn instead of Mn (entry 11). Control experiments confirmed that no reaction occurred in the absence of VB_12_ catalyst (entry 12) and Mn reductant (entry 13). Finally, increasing the amount of olefin 2a (1.5–2.0 equiv.) improved the yield (70–82%, entries 14 and 15).

**Table 1:** Optimisation of the reaction conditions^a^

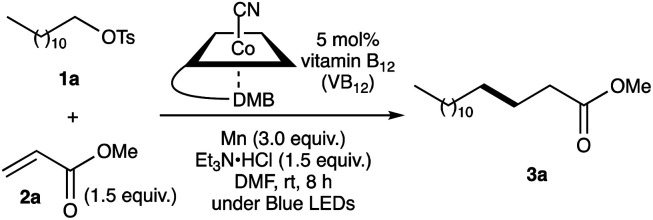		
Entry	Changes from standard conditions	Yield^b^ (%)
1	None	61
2	Without Et_3_N·HCl	10
3	H_2_O instead of Et_3_N·HCl	24
4	NH_4_Cl instead of Et_3_N·HCl	54
5	CoCl(dmgH)_2_Py^c^ instead of VB_12_	Trace
6	Co(salen)^c^ instead of VB_12_	Trace
7	Cp(Pc)^c^ instead of VB_12_	Trace
8	Co(TMPP)^c^ instead of VB_12_	58
9	In ambient conditions^d^	15
10	In dark	5
11	Zn instead of Mn	51
12	Without VB_12_	0
13	Without Mn reductant	0
14	Olefin (1.5 equiv.)	82 (80)
15	Olefin (2.0 equiv.)	70

a Standard reaction conditions: 1a (0.25 mmol), 2a (0.25 mmol), VB_12_ (12.5 μmol, 5 mol%), Et_3_N·HCl (0.38 mmol), Mn powder (0.75 mmol), DMF (1.5 mL); room temperature for 8 h; the blue-light irradiation; argon atmosphere.

b Yields were determined by GC using mesitylene as an internal standard. The parenthesis value indicates the isolated yield.

c See ESI.

d The reaction was performed without blue-light irradiation.

**Fig. 1:**
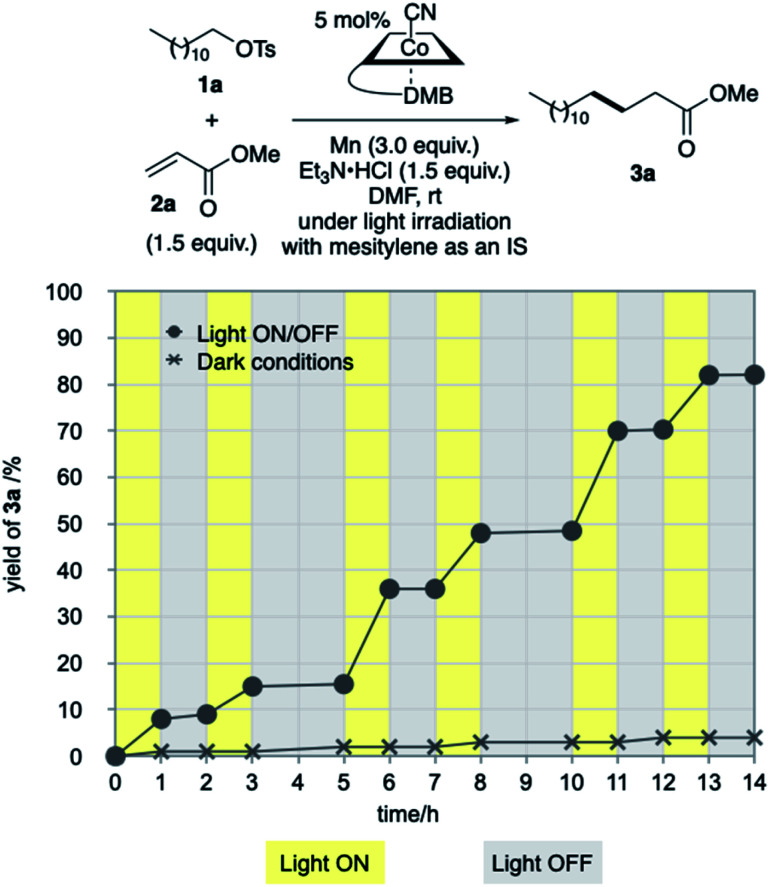
Plots of product yield (%) against reaction time (h) in the cobalt-catalysed Giese reaction of 1a and 2a, ●: under light ON/OFF conditions, ×: in dark.

**Fig. 2:**
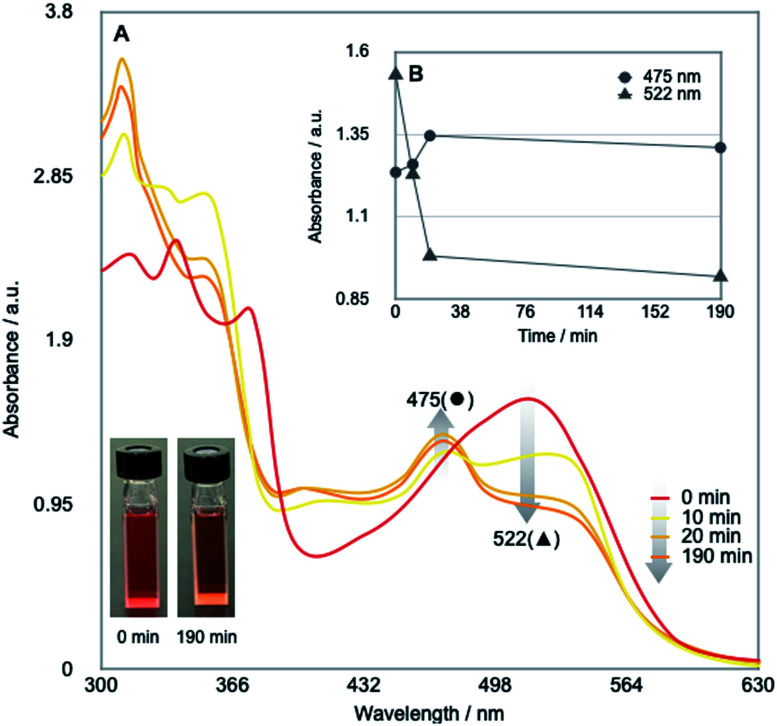
(A) Time-dependent UV-Vis absorption spectra of Me-Cbl in DMF (10^−4^ mol L^−1^) under the blue-light irradiation under argon atmosphere. (B) Variation over time of absorbances at 475 and 522 nm.

We explored the substrate scope for alkyl tosylates and activated olefins with these optimised reaction conditions in hand (Table 2). Various activated olefins possessing amide, ester, ketone, sulfonyl and pinacolboryl functions were well tolerated in the reaction with some primary alkyl tosylates, producing the target products 3b–3h in high yields. Notably, the radical addition was performed on a gram-scale to give the desired adduct 3h in 78% yield.

**Table 2:** Substrate scope of the cobalt-catalysed Giese reaction of alkyl tosylates with activated olefins^e^

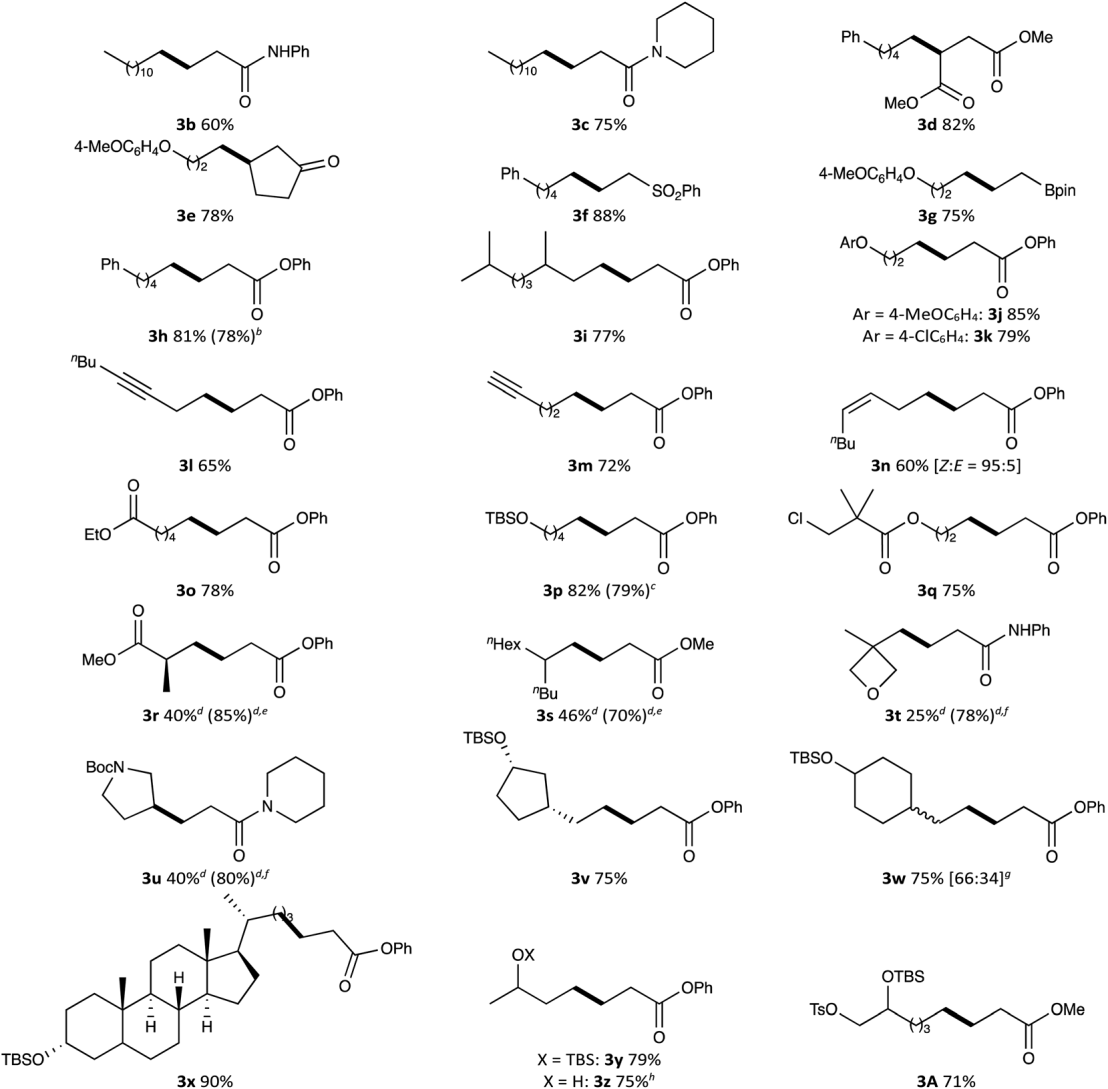

a Reaction conditions: alkyl tosylate (0.25 mmol), activated olefin (0.375 mmol), vitamin B_12_ (12.5 μmol, 5 mol%), Et_3_N·HCl (0.38 mmol), Mn powder (0.75 mmol), DMF (1.5 mL); room temperature for 16 h; the blue-light irradiation; argon atmosphere. Isolated yield.

b The reaction was conducted with 5-phenylpenthyl tosylate (5 mmol) and phenyl acrylate (7.5 mmol).

c 5-(*tert*-Butyldimethylsilyl)-1-chloropenthane was used instead of the corresponding tosylate.

d Reaction time: 24 h.

e 10 mol% of vitamin B_12_ was used.

f Co(TMPP) was used instead of vitamin B_12_.

g Diastereomeric alkyl tosylate [65 : 35] was used. The values in bracket indicate a diastereomeric ratio of the radical adduct.

h See main text.

Next, the substrate scope for alkyl tosylates was evaluated. Branched alkyl chain (for 3i), aryl ethers (for 3j and 3k), internal (for 3l) and terminal alkynyl groups (for 3m), a *cis*-configurated alkenyl group (for 3n) and an ester (for 3o) afforded the corresponding radical adducts in good yields (60–85%). An alkyl chloride was also suitable for this reaction, being converted to radical adduct 3p in 79% yield. Interestingly, a chloro-substituent located at the sterically congested position did not participate in the transformation; chloro-substituted radical adduct 3q was selectively obtained in 75% yield. In the alkyl radical generation from alkyl tosylates, steric factors can be expected to affect the S_N_2 displacement of nucleophilic cobalt(i). Indeed, the conversion of bulky alkyl tosylates such as 3r–3u was slow under the optimised conditions. The reactivity was improved by increasing the catalyst loading up to 10 mol% (for 3r and 3s) or using a smaller planar-cobalt catalyst like Co(TMPP) (for 3t and 3u), providing the corresponding adducts in 70–85% yields, even in the reaction with secondary alkyl tosylate like 3-pyrrolidinyl tosylate. Unfortunately, secondary alkyl tosylates such as cyclohexyl and 2-hexyl tosylates were not compatible with the reaction. The low reactivity associated with the steric repulsion between the cobalt catalyst and the tosylate is a drawback of this reaction; however, it could be also considered a peculiarity. Thus, since in the tosylation of alkyl polyols the tosylation selectively occurs at the primary alcoholic position,^20^ multi-functionalised primary alkyl tosylates could be readily synthesised by the tosylation of polyols and subsequent protection of the remaining hydroxy groups. We believed that a regioselective Giese reaction starting from polyols could be envisaged by combining the present radical addition with the classical tosylation. This strategy enabled the regioselective transformation of various diols bearing primary and secondary alcoholic moieties for the synthesis of functionalised alcohol derivatives 3v–3y. Although the present reaction was strongly inhibited by a naked hydroxyl group, pre-treatment of 3-hydroxylbutyl tosylate with trimethylsilyl chloride (1.2 equiv.) in the presence of Mn (4.2 equiv.) at 25 °C for 3 h enabled the direct conversion of the hydroxylated alkyl tosylate to the corresponding radical adduct 3z in 75% yield without any deprotection process. A selective Giese reaction of a triol derivative having one TBSO and two TsO groups in different steric environments was also demonstrated, in which the reaction proceeded at the less bulky TsO site without lacking the other TsO group (for 3A).

The reaction of 5-hexenyl tosylate (4) with phenyl acrylate under the standard conditions afforded a mixture of cyclised 5 and linear adducts 6 in 19% and 34% yields, respectively (Scheme 2, eqn (1)). Additionally, the VB_12_-catalysed reaction of dodecyl tosylate (1a) in the presence of γ-terpinene (3.0 equiv.) as a hydrogen atom donor^21^ afforded dodecane (7) in 80% GC yield. These results support the generation of alkyl radicals during the reaction. Additionally, in the absence of alkyl tosylate, phenyl acrylate produced diphenyl adipate in 30% yield and 60% conversion (Scheme 2, eqn (2)). This result indicates that the nucleophilic cobalt generated *in situ* could react faster with alkyl tosylates than with activated olefins.^22^

**Scheme 2:**
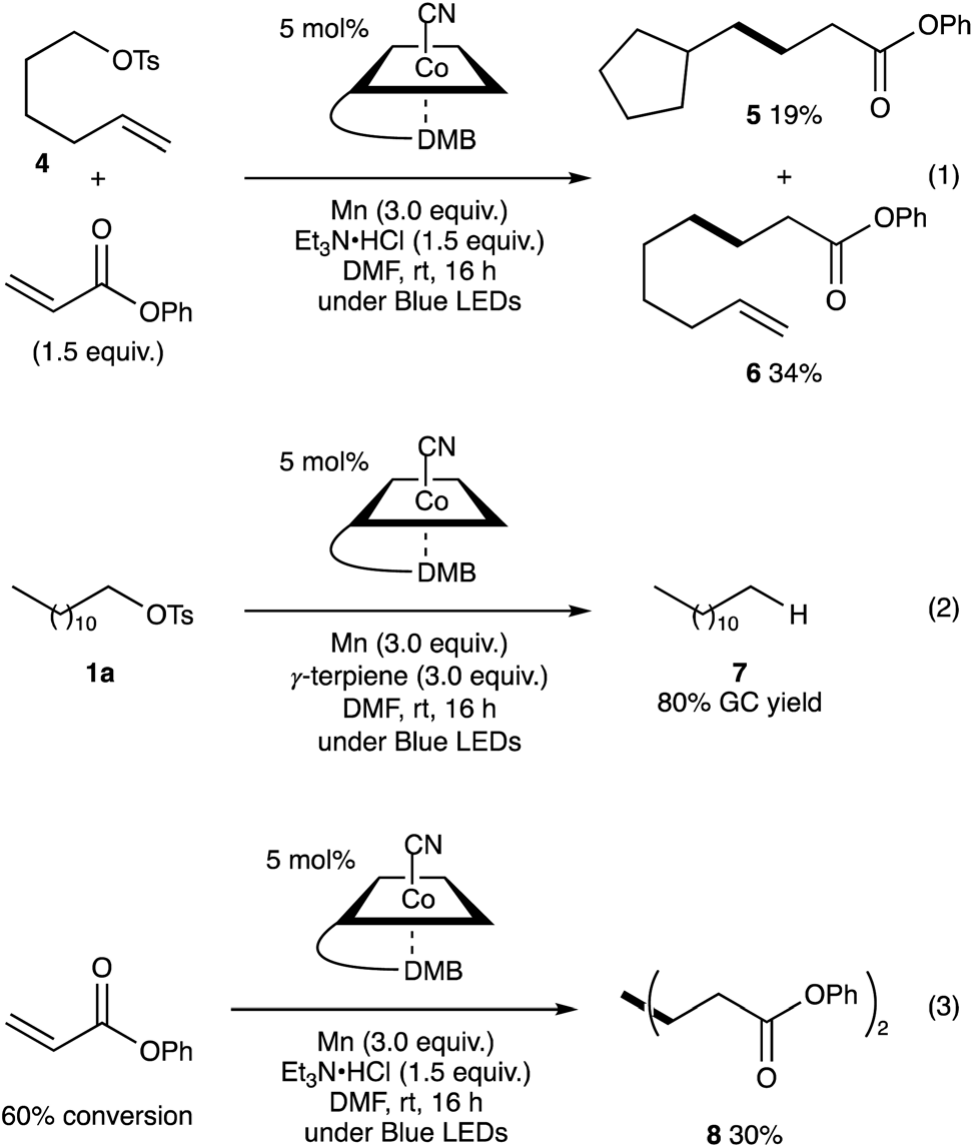
Radical clock experiment using 5-hexenyl tosylate 6 (eqn (1)). Radical trapping reaction with γ-terpinene (eqn (2)). Reductive dimerization of phenyl acrylate (eqn (3)).

According to these results, we tentatively proposed a catalytic cycle depicted in Scheme 3. The S_N_2-type oxidative addition of alkyl tosylates to nucleophilic cobalt(i) A would afford alkyl-cobalt(iii) B, which would undergo the Co–C bond cleavage under the visible-light irradiation to generate an alkyl radical and planar-cobalt(ii) C. The generated alkyl radical would add to the β-position of an activated olefin, followed by reduction with Mn and protonation to give adduct 3. The catalytic cycle would be closed by Mn reduction of planar-cobalt(ii) C to regenerate the nucleophilic cobalt(i) A.

**Scheme 3:**
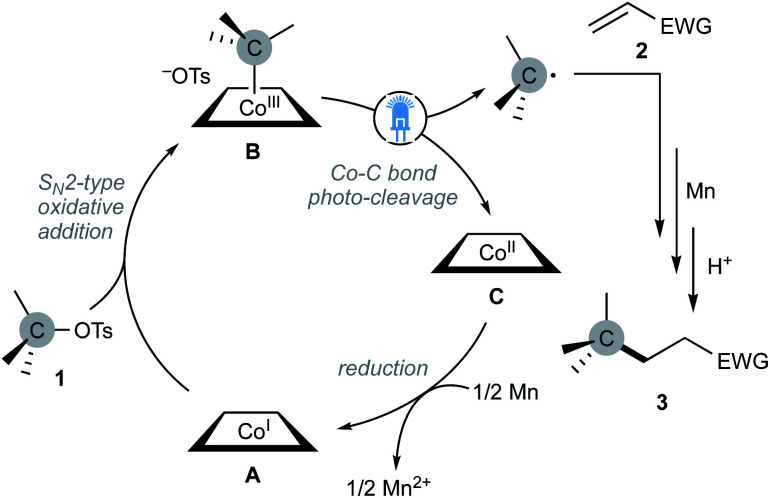
Plausible reaction mechanism of nucleophilic cobalt-catalysed Giese reaction with alkyl tosylates under the light irradiation.

In conclusion, we have demonstrated a Giese reaction utilising readily available alkyl tosylates as alkyl radical sources for the first time, which proceeded efficiently in the presence of vitamin B_12_ or Co(TTMP) catalysts, Mn reductant, and a proton donor under the visible-light irradiation. The critical step in this reaction is the generation of alkyl-cobalt(iii) intermediates by S_N_2-type oxidative addition of alkyl tosylates to nucleophilic cobalt(i). Hence, the bulkiness around the TsO group on alkyl tosylates affected the reaction efficiency. Nevertheless, classical tosylation and subsequent Giese reaction enabled the regioselective functionalisation of polyol compounds. Further studies on the alkyl radical generation protocol to extend the scope of this strategy to novel organic transformations are now ongoing in our laboratory.

## Experimental

### General procedure for cobalt-catalysed Giese reaction of alkyl tosylates

In a Schlenk tube equipped with a stirring bar, Mn powder (41.2 mg, 0.75 mmol) was added and heated at 400 °C for 3 min under vacuum. After cooling and filling with argon, the tube was added triethylamine hydrochloride Et_3_N·HCl (51.6 mg, 0.38 mmol) and vitamin B_12_ (VB_12_; 16.9 mg, 12.5 μmol), evacuated and filled with argon three times. After adding dry DMF (1.5 mL) and stirring for 5 min, the color instantly changed to green by adding trimethylsilyl chloride (*ca.* 6 μL). Then, alkyl tosylate (0.25 mmol) and activated olefin (0.35 mmol) were added to the solution. The reaction mixture was stirred at room temperature for the appropriate time (18–24 h) under blue-light irradiation using a PhotoRedOx Box (HeptoChem Inc.) equipped with a Kessil A160W TUNE BLUE (*λ* = 454 nm, 40 W) as shown in Fig. S1 (ESI).† The color of the reaction mixture drastically changed in each step, as depicted in Fig. S2 (ESI).† The obtained mixture was diluted with ethyl acetate and quenched by saturated aqueous NH_4_Cl. The aqueous phase was extracted with ethyl acetate, and the combined organic phase was dried over anhydrous MgSO_4_. After filtration and removal of the solvent, the residue was purified by silica-gel flash column chromatography to give the radical adduct.

### Procedure for direct Giese reaction of 3-hydroxylbutyl tosylate: synthesis of 3z

In a Schlenk tube equipped with a stirring bar, Mn powder (57.7 mg, 1.05 mmol) was added and heated at 400 °C for 3 min under vacuum. After cooling and filling with argon, the tube was added Et_3_N·HCl (51.6 mg, 0.38 mmol), DMF (1.5 mL) and 3-hydroxylbutyl tosylate (61.1 mg, 0.25 mmol). Trimethylsilyl chloride (38 μL, 0.30 mmol) was added into the mixture. The solution was stirred for 3 h at 25 °C. Then, vitamin B_12_ (VB_12_; 16.9 mg, 12.5 μmol) and phenyl acrylate (55.6 mg, 0.38 mmol) were successively added. The reaction mixture was stirred at room temperature for 16 h under blue-light irradiation. After reaction, the obtained mixture was diluted with ethyl acetate and quenched by saturated aqueous NH_4_Cl. The aqueous phase was extracted with ethyl acetate, and the combined organic phase was dried over anhydrous MgSO_4_. After filtration and removal of the solvent, the residue was purified by silica-gel flash column chromatography using as 10% ethyl acetate in hexane an eluent to give the radical adduct 3z (41.7 mg, colorless oil) in 75% yield.

### Characterisation of products

#### 3a

Isolated as a colorless oil in 80% yield; ^1^H NMR (500 MHz, CDCl_3_) *δ* 3.66 (s, 3H), 2.30 (t, *J* = 7.6 Hz, 2H), 1.66–1.57 (m, 2H), 1.35–1.16 (m, 22H), 0.87 (t, *J* = 7.0 Hz, 3H); ^13^C NMR (126 MHz, CDCl_3_) *δ* 174.4, 51.4, 34.1, 31.9, 29.7, 29.7, 29.6, 29.6, 29.4, 29.3, 29.2, 29.1, 24.9, 22.7, 14.1; HRMS calcd for C_16_H_33_O_2_ [M + H]^+^: 257.2481, found 257.2479.

#### 3b

Isolated as a colorless oil in 60% yield; ^1^H NMR (500 MHz, CDCl_3_) *δ* 7.51 (d, *J* = 8.0 Hz, 2H), 7.35–7.28 (m, 2H), 7.13–7.06 (m, 1H), 2.35 (t, *J* = 7.6 Hz, 2H), 1.73 (p, *J* = 7.5 Hz, 2H), 1.42–1.27 (m, 4H), 1.25 (s, 19H), 0.88 (t, *J* = 6.9 Hz, 3H); ^13^C NMR (126 MHz, CDCl_3_) *δ* 171.3, 138.0, 129.0, 124.1, 119.7, 37.9, 31.9, 29.7, 29.7, 29.6, 29.6, 29.5, 29.4, 29.3, 29.3, 25.6, 22.7, 14.1; HRMS calcd for C_21_H_36_NO [M + H]^+^: 318.2797, found 304.2642.

#### 3c

Isolated as a colorless oil in 75% yield; ^1^H NMR (500 MHz, CDCl_3_) *δ* 3.54 (t, *J* = 5.6 Hz, 2H), 3.38 (t, *J* = 5.4 Hz, 2H), 2.34–2.26 (m, 2H), 1.71–1.44 (m, 10H), 1.24 (s, 24H), 0.87 (t, *J* = 6.8 Hz, 3H); ^13^C NMR (126 MHz, CDCl_3_) *δ* 171.5, 46.7, 42.6, 33.5, 31.9, 29.7, 29.7, 29.6, 29.6, 29.5, 29.5, 29.4, 29.3, 26.6, 25.6, 25.5, 24.6, 22.7, 14.1; HRMS calcd for C_20_H_40_NO [M + H]^+^: 310.3110, found 310.3108.

#### 3d

Isolated as a colorless oil in 82% yield; ^1^H NMR (500 MHz, CDCl_3_) *δ* 7.27 (t, *J* = 7.5 Hz, 2H), 7.17 (t, *J* = 8.8 Hz, 3H), 3.69 (s, 3H), 3.67 (s, 3H), 2.83 (dddd, *J* = 9.3, 7.4, 6.3, 5.1 Hz, 1H), 2.71 (dd, *J* = 16.5, 9.3 Hz, 1H), 2.62–2.55 (m, 2H), 2.42 (dd, *J* = 16.5, 5.2 Hz, 1H), 1.69–1.56 (m, 3H), 1.55–1.45 (m, 1H), 1.32 (qt, *J* = 6.6, 2.6 Hz, 4H); ^13^C NMR (126 MHz, CDCl_3_) *δ* 175.4, 172.4, 142.5, 128.4, 128.2, 125.6, 51.8, 51.7, 41.1, 35.8, 31.8, 31.2, 29.0, 26.8; HRMS calcd for C_17_H_25_O_4_ [M + H]^+^: 293.1753, found 293.1754.

#### 3e

Isolated as a colorless oil in 78% yield; ^1^H NMR (500 MHz, CDCl_3_) *δ* 6.83 (s, 4H), 3.92 (t, *J* = 6.3 Hz, 2H), 3.76 (s, 3H), 2.46–2.37 (m, 1H), 2.37–2.26 (m, 1H), 2.28–2.10 (m, 3H), 1.89–1.73 (m, 3H), 1.69–1.46 (m, 3H); ^13^C NMR (126 MHz, CDCl_3_) *δ* 219.5, 153.8, 153.1, 115.4, 114.6, 68.4, 55.7, 45.2, 38.5, 37.0, 32.1, 29.5, 27.7; HRMS calcd for C_15_H_21_O_3_ [M + H]^+^: 249.1491, found 249.1493.

#### 3f

Isolated as a colorless oil in 88% yield; ^1^H NMR (500 MHz, CDCl_3_) *δ* 7.91 (dd, *J* = 8.4, 1.4 Hz, 2H), 7.69–7.62 (m, 1H), 7.60–7.53 (m, 2H), 7.30–7.23 (m, 2H), 7.20–7.12 (m, 3H), 3.11–3.04 (m, 2H), 2.61–2.54 (m, 2H), 1.70 (ddt, *J* = 13.4, 10.6, 6.4 Hz, 2H), 1.61–1.53 (m, 2H), 1.41–1.19 (m, 6H); ^13^C NMR (126 MHz, CDCl_3_) *δ* 142.5, 139.1, 133.6, 129.2, 128.3, 128.2, 128.0, 125.6, 56.2, 35.8, 31.2, 28.8, 28.8, 28.1, 22.5; HRMS calcd for C_19_H_25_O_2_S [M + H]^+^: 317.1575, found 317.1574.

#### 3g

Isolated as a colorless oil in 75% yield; ^1^H NMR (500 MHz, CDCl_3_) *δ* 6.82 (s, 4H), 3.89 (t, *J* = 6.6 Hz, 2H), 3.76 (s, 3H), 1.88 (ddt, *J* = 16.2, 9.6, 6.8 Hz, 2H), 1.75 (p, *J* = 6.8 Hz, 1H), 1.46 (dtd, *J* = 7.8, 5.0, 2.4 Hz, 2H), 1.28–1.20 (m, 13H), 0.80 (t, *J* = 7.4 Hz, 2H); ^13^C NMR (126 MHz, CDCl_3_) *δ* 153.6, 153.3, 115.4, 114.6, 82.9, 68.6, 55.7, 29.7, 29.1, 24.8, 23.8, one signal was obscured; HRMS calcd for C_18_H_30_BO_4_ [M + H]^+^: 321.2237, found 321.2235.

#### 3h

Isolated as a colorless oil in 81% yield; ^1^H NMR (500 MHz, CDCl_3_) *δ* 7.44–7.35 (m, 2H), 7.35–7.30 (m, 1H), 7.30–7.15 (m, 5H), 7.14–7.05 (m, 2H), 2.67–2.60 (m, 2H), 2.57 (t, *J* = 7.5 Hz, 2H), 1.77 (t, *J* = 7.3 Hz, 2H), 1.70–1.60 (m, 2H), 1.48–1.34 (m, 6H); ^13^C NMR (126 MHz, CDCl_3_) *δ* 172.3, 150.7, 142.7, 129.4, 128.4, 128.2, 125.7, 125.6, 121.5, 35.9, 34.3, 31.4, 29.1, 29.1, 29.0, 24.9; HRMS calcd for C_20_H_24_O_2_ [M + H]^+^: 297.1855, found 297.1850.

#### 3i

Isolated as a colorless oil in 77% yield; ^1^H NMR (500 MHz, CDCl_3_) *δ* 7.41–7.34 (m, 2H), 7.25–7.19 (m, 1H), 7.11–7.05 (m, 2H), 2.56 (t, *J* = 7.5 Hz, 2H), 1.82–1.66 (m, 2H), 1.53 (dt, *J* = 13.3, 6.6 Hz, 1H), 1.49–1.03 (m, 11H), 0.87 (d, *J* = 6.6 Hz, 9H); ^13^C NMR (126 MHz, CDCl_3_) *δ* 172.3, 150.7, 129.4, 125.7, 121.6, 39.3, 37.2, 36.6, 34.4, 32.6, 28.0, 26.6, 25.3, 24.8, 22.7, 22.6, 19.6; HRMS calcd for C_19_H_30_O_2_ [M + H]^+^: 291.2324, found 291.2326.

#### 3j

Isolated as a colorless oil in 85% yield; ^1^H NMR (500 MHz, CDCl_3_) *δ* 7.42–7.35 (m, 2H), 7.28–7.20 (m, 1H), 7.13–7.05 (m, 2H), 6.85 (s, 3H), 3.95 (t, *J* = 6.4 Hz, 2H), 3.77 (s, 3H), 2.61 (t, *J* = 7.5 Hz, 2H), 1.84 (ddd, *J* = 13.7, 8.8, 6.7 Hz, 5H), 1.61 (ddt, *J* = 9.5, 7.0, 2.0 Hz, 2H); ^13^C NMR (126 MHz, CDCl_3_) *δ* 172.1, 153.7, 153.1, 150.7, 129.4, 125.7, 121.5, 115.4, 114.6, 68.2, 55.7, 34.2, 29.0, 25.6, 24.6; HRMS calcd for C_19_H_22_O_4_ [M + H]^+^: 315.1596, found 315.1597.

#### 3k

Isolated as a colorless oil in 79% yield; ^1^H NMR (500 MHz, CDCl_3_) *δ* 7.42–7.34 (m, 2H), 7.25–7.20 (m, 3H), 7.11–7.05 (m, 2H), 6.86–6.79 (m, 2H), 3.95 (t, *J* = 6.4 Hz, 2H), 2.61 (t, *J* = 7.4 Hz, 2H), 1.89–1.79 (m, 4H), 1.66–1.56 (m, 2H); ^13^C NMR (126 MHz, CDCl_3_) *δ* 172.0, 157.6, 150.6, 129.4, 129.2, 125.7, 125.3, 121.5, 115.7, 67.8, 34.2, 28.8, 25.5, 24.6.; HRMS calcd for C_18_H_20_ClO_3_ [M + H]^+^: 319.1101, found 319.1099.

#### 3l

Isolated as a colorless oil in 65% yield; ^1^H NMR (500 MHz, CDCl_3_) *δ* 7.41–7.34 (m, 2H), 7.22 (ddt, *J* = 7.7, 7.0, 1.1 Hz, 1H), 7.11–7.04 (m, 2H), 2.58 (dd, *J* = 7.9, 7.1 Hz, 2H), 2.23 (tt, *J* = 7.0, 2.4 Hz, 2H), 2.15 (tt, *J* = 7.1, 2.4 Hz, 2H), 1.91–1.82 (m, 2H), 1.66–1.57 (m, 2H), 1.51–1.34 (m, 4H), 0.90 (t, *J* = 7.2 Hz, 3H); ^13^C NMR (126 MHz, CDCl_3_) *δ* 172.0, 150.7, 129.4, 125.7, 121.5, 80.8, 79.3, 33.9, 31.2, 28.4, 24.1, 21.9, 18.5, 18.4, 13.6; HRMS calcd for C_17_H_23_O_2_ [M + H]^+^: 259.1698, found 259.1696.

#### 3m

Isolated as a colorless oil in 72% yield; ^1^H NMR (500 MHz, CDCl_3_) *δ* 7.38 (t, *J* = 8.0 Hz, 2H), 7.22 (t, *J* = 7.4 Hz, 1H), 7.07 (d, *J* = 7.6 Hz, 2H), 2.58 (t, *J* = 7.5 Hz, 2H), 2.23 (td, *J* = 6.8, 2.6 Hz, 2H), 1.96 (t, *J* = 2.6 Hz, 1H), 1.78 (p, *J* = 7.4 Hz, 2H), 1.65–1.49 (m, 4H); ^13^C NMR (126 MHz, CDCl_3_) *δ* 172.1, 150.7, 129.4, 125.7, 121.6, 84.3, 68.4, 34.2, 28.1, 28.1, 24.4, 18.3; HRMS calcd for C_14_H_17_O_2_ [M + H]^+^: 217.1229, found 217.1227.

#### 3n

Isolated as *cis*-rich stereoisomers (*cis*/*trans* = 95 : 5, colorless oil) in 60% yield; ^1^H NMR (500 MHz, CDCl_3_) *δ* 7.41–7.33 (m, 2H), 7.28–7.15 (m, 1H), 7.11–7.04 (m, 2H), 5.40 (dtt, *J* = 10.8, 6.8, 1.4 Hz, 1H), 5.36 (dtt, *J* = 10.8, 6.9, 1.4 Hz, 1H), 2.57 (t, *J* = 7.4 Hz, 2H), 2.16–1.99 (m, 3H), 1.83–1.73 (m, 2H), 1.57 (s, 1H), 1.53–1.43 (m, 2H), 1.38–1.24 (m, 4H), 0.90 (t, *J* = 7.1 Hz, 3H); ^13^C NMR (126 MHz, CDCl_3_) *δ* 172.2, 150.7, 130.6, 129.4, 129.0, 125.7, 121.6, 34.3, 31.9, 29.1, 26.9, 26.8, 24.6, 22.3, 14.0; HRMS calcd for C_17_H_25_O_2_ [M + H]^+^: 261.1855, found 261.1859.

#### 3o

Isolated as a colorless oil in 78% yield; ^1^H NMR (500 MHz, CDCl_3_) *δ* 7.41–7.33 (m, 2H), 7.22 (ddt, *J* = 7.1, 6.5, 1.1 Hz, 1H), 7.10–7.03 (m, 2H), 4.12 (q, *J* = 7.1 Hz, 2H), 2.55 (t, *J* = 7.5 Hz, 2H), 2.30 (t, *J* = 7.5 Hz, 2H), 1.79–1.71 (m, 2H), 1.68–1.57 (m, 2H), 1.47–1.30 (m, 6H), 1.25 (t, *J* = 7.1 Hz, 3H); ^13^C NMR (126 MHz, CDCl_3_) *δ* 173.8, 172.2, 150.7, 129.4, 125.7, 121.5, 60.2, 34.3, 34.3, 28.9, 28.9, 24.9, 24.8, 14.2; HRMS calcd for C_17_H_24_O_4_ [M + H]^+^: 293.1753, found 293.1755.

#### 3p

Isolated as a colorless oil in 82% yield; ^1^H NMR (500 MHz, CDCl_3_) *δ* 7.37 (dd, *J* = 8.5, 7.4 Hz, 2H), 7.27–7.18 (m, 1H), 7.08 (dd, *J* = 8.7, 1.2 Hz, 2H), 3.61 (t, *J* = 6.6 Hz, 2H), 2.56 (t, *J* = 7.5 Hz, 2H), 1.76 (p, *J* = 7.5 Hz, 2H), 1.58–1.49 (m, 2H), 1.41–1.31 (m, 6H), 0.90 (s, 9H), 0.06 (s, 6H); ^13^C NMR (126 MHz, CDCl_3_) *δ* 172.3, 150.7, 129.4, 125.7, 121.5, 63.2, 34.4, 32.8, 29.1, 29.0, 26.0, 25.6, 24.9, 18.4, −5.3.; HRMS calcd for C_20_H_35_O_3_Si [M + H]^+^: 351.2355, found 351.2359.

#### 3q

Isolated as a yellow-viscous oil in 75% yield; ^1^H NMR (500 MHz, CDCl_3_) *δ* 7.41–7.34 (m, 2H), 7.22 (td, *J* = 7.3, 1.1 Hz, 1H), 7.11–7.04 (m, 2H), 4.15 (t, *J* = 6.5 Hz, 2H), 3.61 (s, 2H), 2.58 (t, *J* = 7.5 Hz, 2H), 1.80 (p, *J* = 7.5 Hz, 1H), 1.72 (dq, *J* = 8.2, 6.6 Hz, 0H), 1.56–1.46 (m, 2H), 1.29 (s, 6H); ^13^C NMR (126 MHz, CDCl_3_) *δ* 175.0, 172.0, 150.7, 129.4, 125.8, 121.5, 64.7, 52.1, 44.6, 34.2, 28.3, 25.5, 24.5, 23.2; HRMS calcd for C_17_H_24_ClO_4_ [M + H]^+^: 327.1363, found 327.1365.

#### 3r

Isolated as a colorless oil in 85% yield; ^1^H NMR (500 MHz, CDCl_3_) *δ* 7.38 (t, *J* = 7.8 Hz, 2H), 7.22 (t, *J* = 7.4 Hz, 1H), 7.08 (t, *J* = 6.6 Hz, 2H), 3.69 (s, 3H), 2.64 (ddt, *J* = 7.1, 4.4, 2.3 Hz, 1H), 2.60–2.54 (m, 1H), 2.50 (p, *J* = 6.9 Hz, 1H), 1.93–1.86 (m, 1H), 1.84–1.68 (m, 2H), 1.61–1.49 (m, 1H), 1.20 (d, *J* = 7.0 Hz, 3H); ^13^C NMR (126 MHz, CDCl_3_) *δ* 176.8, 171.8, 150.6, 129.4, 125.7, 121.5, 51.6, 39.2, 34.1, 33.0, 24.3, 22.6, 17.1; HRMS calcd for C_14_H_19_O_4_ [M + H]^+^: 251.1283, found 251.1286.

#### 3s

Isolated as a yellow-viscous oil in 70% yield; ^1^H NMR (500 MHz, CDCl_3_) *δ* 3.67 (s, 3H), 2.28 (t, *J* = 7.6 Hz, 2H), 1.64–1.54 (m, 3H), 1.34–1.24 (m, 12H), 1.28–1.19 (m, 6H), 0.88 (td, *J* = 7.0, 2.3 Hz, 6H); ^13^C NMR (126 MHz, CDCl_3_) *δ* 174.3, 51.4, 37.2, 34.5, 33.5, 33.2, 33.1, 31.9, 29.8, 28.9, 26.6, 23.1, 22.7, 22.2, 14.2, 14.1; HRMS calcd for C_16_H_33_O_2_ [M + H]^+^: 257.2481, found 257.2492.

#### 3t

Isolated as a red oil in 78% yield; ^1^H NMR (400 MHz, CDCl_3_) *δ* 7.51 (d, *J* = 7.9 Hz, 2H), 7.36 (s, 1H), 7.31 (t, *J* = 7.9 Hz, 2H), 7.10 (t, *J* = 7.4 Hz, 1H), 4.43 (d, *J* = 5.6 Hz, 2H), 4.35 (d, *J* = 5.6 Hz, 2H), 2.41–2.33 (m, 2H), 1.76–1.64 (m, 4H), 1.30 (s, 3H); ^13^C NMR (126 MHz, CDCl_3_) *δ* 170.8, 137.8, 129.0, 124.2, 119.8, 82.7, 39.1, 38.5, 37.7, 23.1, 20.5; HRMS calcd for C_14_H_20_NO_2_ [M + H]^+^: 234.1494, found 234.1491.

#### 3u

Isolated as a colorless oil in 80% yield; ^1^H NMR (500 MHz, CDCl_3_) *δ* 3.60–3.45 (m, 3H), 3.38 (dq, *J* = 10.9, 5.0 Hz, 3H), 3.24 (dtd, *J* = 16.5, 10.0, 7.0 Hz, 1H), 2.96–2.81 (m, 1H), 2.41–2.26 (m, 2H), 2.23–2.08 (m, 1H), 2.06–1.93 (m, 1H), 1.84–1.60 (m, 5H), 1.60–1.49 (m, 4H), 1.45 (s, 9H); ^13^C NMR (126 MHz, CDCl_3_) *δ* 170.6, 154.6, 79.0, 51.5, 50.9, 46.6, 45.7, 45.4, 42.7, 38.8, 37.9, 31.8, 31.8, 31.0, 29.7, 28.6, 28.5, 26.5, 25.5, 24.5; HRMS calcd for C_17_H_31_N_2_O_3_ [M + H]^+^: 311.2335, found 311.2339.

#### 3v

Isolated as a colorless oil in 75% yield; ^1^H NMR (500 MHz, CDCl_3_) *δ* 7.41–7.35 (m, 2H), 7.22 (ddt, *J* = 7.8, 7.0, 1.1 Hz, 1H), 7.10–7.05 (m, 2H), 4.20 (q, *J* = 5.9 Hz, 1H), 2.56 (t, *J* = 7.5 Hz, 2H), 2.06–1.98 (m, 1H), 1.84–1.65 (m, 5H), 1.62–1.52 (m, 1H), 1.47–1.31 (m, 5H), 1.17 (dddd, *J* = 12.8, 8.9, 6.2, 0.8 Hz, 1H), 0.89 (s, 9H), 0.04 (s, 6H); ^13^C NMR (126 MHz, CDCl_3_) *δ* 172.3, 150.7, 129.4, 125.7, 121.6, 74.1, 42.6, 37.5, 36.4, 35.3, 34.4, 29.9, 28.0, 25.9, 25.1, 18.1, −4.7, −4.7; HRMS calcd for C_22_H_37_O_3_Si [M + H]^+^: 377.2512, found 77.2510.

#### 3w

Isolated as a *cis*/*trans*-mixture (colorless oil) in 75% [66 : 34] yield; ^1^H NMR (500 MHz, CDCl_3_) *δ* 7.43–7.34 (m, 2H), 7.22 (ddd, *J* = 8.0, 7.0, 1.1 Hz, 1H), 7.08 (ddt, *J* = 7.6, 2.0, 1.0 Hz, 2H), 3.92 (q, *J* = 3.5, 3.1 Hz, 0.66H), 3.56–3.46 (m, 0.34H), 2.56 (tdd, *J* = 7.6, 3.1, 0.9 Hz, 2H), 1.88–1.81 (m, 1H), 1.79–1.68 (m, 3H), 1.66–1.56 (m, 2H), 1.40 (p, *J* = 4.0, 3.5 Hz, 2H), 1.34–1.19 (m, 4H), 1.22 (s, 1H), 0.90 (d, *J* = 2.7 Hz, 9H), 0.06 (s, 1H); ^13^C NMR (126 MHz, CDCl_3_) *δ* 172.3, 172.2, 150.8, 129.5, 129.2, 125.8, 125.5, 121.7, 121.4, 72.0, 71.9, 67.4, 67.2, 36.4, 36.0, 34.6, 34.4, 34.4, 34.2, 33.3, 33.2, 31.5, 31.3, 27.1, 26.7, 26.5, 26.0, 26.0, 25.9, 25.9, 25.9, 25.8, 25.7, 25.2, 25.0, 18.2, 18.1, −4.5, −4.7, −4.7, −4.9; HRMS calcd for C_23_H_39_O_3_Si [M + H]^+^: 391.2668, found 391.2668.

#### 3x

Isolated as a yellow viscous oil in 90% yield; ^1^H NMR (500 MHz, CDCl_3_) *δ* 7.37 (t, *J* = 7.7 Hz, 2H), 7.22 (t, *J* = 7.5 Hz, 1H), 7.08 (d, *J* = 8.6 Hz, 2H), 3.58 (tt, *J* = 10.5, 4.6 Hz, 1H), 2.55 (t, *J* = 7.5 Hz, 2H), 1.96 (dt, *J* = 12.4, 3.2 Hz, 1H), 1.88–1.70 (m, 7H), 1.59–1.51 (m, 2H), 1.50–1.31 (m, 13H), 1.29–0.83 (m, 9H), 0.94–0.86 (m, 15H), 0.64 (s, 3H), 0.06 (s, 6H); ^13^C NMR (126 MHz, CDCl_3_) *δ* 172.3, 150.7, 129.3, 125.7, 121.6, 72.8, 56.4, 56.2, 42.7, 42.3, 40.2, 40.1, 36.9, 35.8, 35.7, 35.6, 34.6, 34.4, 31.0, 29.6, 28.3, 27.3, 26.4, 26.0, 25.7, 25.0, 24.2, 23.4, 20.8, 18.6, 18.3, 12.0, −4.6; HRMS calcd for C_39_H_65_O_3_Si [M + H]^+^: 609.4703, found 609.4701.

#### 3y

Isolated as a thin-yellow oil in 79% yield; ^1^H NMR (500 MHz, CDCl_3_) *δ* 7.41–7.33 (m, 2H), 7.25–7.18 (m, 1H), 7.10–7.03 (m, 2H), 3.86–3.76 (m, 1H), 2.56 (t, *J* = 7.5 Hz, 2H), 1.82–1.70 (m, 2H), 1.56–1.35 (m, 4H), 1.13 (d, *J* = 6.1 Hz, 3H), 0.89 (s, 9H), 0.05 (s, 3H), 0.05 (s, 3H); ^13^C NMR (126 MHz, CDCl_3_) *δ* 172.2, 150.7, 129.4, 125.7, 121.6, 68.4, 39.3, 34.4, 25.9, 25.3, 25.0, 23.8, 18.1, −4.4, −4.7; HRMS calcd for C_19_H_33_O_3_Si [M + H]^+^: 337.2199, found 337.2185.

#### 3z

Isolated as a colorless oil in 75% yield; ^1^H NMR (500 MHz, CDCl_3_) *δ* 7.41–7.34 (m, 2H), 7.25–7.18 (m, 1H), 7.10–7.05 (m, 2H), 3.87–3.78 (m, 1H), 2.58 (t, *J* = 7.5 Hz, 2H), 1.86–1.70 (m, 2H), 1.59–1.41 (m, 5H), 1.20 (d, *J* = 6.1 Hz, 3H); ^13^C NMR (126 MHz, CDCl_3_) *δ* 172.2, 150.7, 129.4, 125.7, 121.5, 67.8, 38.8, 34.3, 25.2, 24.8, 23.5; HRMS calcd for C_13_H_19_O_3_ [M + H]^+^: 223.1334, found 223.1339.

#### 3A

Isolated as a colorless oil in 71% yield; ^1^H NMR (500 MHz, CDCl_3_) *δ* 7.81–7.75 (m, 2H), 7.37–7.31 (m, 2H), 3.90–3.77 (m, 3H), 3.66 (s, 3H), 2.45 (s, 3H), 2.29 (t, *J* = 7.5 Hz, 2H), 1.64–1.54 (m, 3H), 1.45–1.33 (m, 1H), 1.32–1.18 (m, 6H), 0.83 (s, 9H), 0.01 (s, 3H), −0.00 (s, 3H); ^13^C NMR (126 MHz, CDCl_3_) *δ* 174.2, 144.7, 133.0, 129.8, 127.9, 73.1, 69.9, 51.4, 34.0, 33.9, 29.2, 28.9, 25.7, 24.8, 24.6, 21.6, 18.0, −4.6, −4.8; HRMS calcd for C_23_H_41_O_6_SSi [M + H]^+^: 473.2393, found 473.2389.

Isolated as a colorless mixture of 5 and 6 (5 : 6 = 65 : 35) in 53% yield; ^1^H NMR (500 MHz, CDCl_3_) *δ* 7.44–7.31 (m, 2H), 7.29–7.18 (m, 1H), 7.14–7.02 (m, 2H), 5.82 (ddt, *J* = 16.9, 10.2, 6.7 Hz, 0.53H), 5.01 (dq, *J* = 17.1, 1.7 Hz, 0.52H), 4.95 (ddt, *J* = 10.2, 2.3, 1.2 Hz, 0.53H), 2.56 (td, *J* = 7.5, 1.0 Hz, 2H), 2.07 (tdd, *J* = 6.7, 5.3, 1.5 Hz, 1H), 1.88–1.70 (m, 3H), 1.69–1.48 (m, 2H), 1.48–1.32 (m, 4H), 1.18–1.03 (m, 1H); ^13^C NMR (126 MHz, CDCl_3_) *δ* 172.3, 172.2, 150.7, 150.7, 138.9, 129.4, 125.7, 125.7, 121.6, 121.5, 114.3, 39.8, 35.6, 34.6, 34.3, 33.7, 32.6, 28.9, 28.7, 28.7, 25.1, 24.9, 24.2; HRMS calcd for C_15_H_21_O_2_ [M + H]^+^: 233.1542, found 233.1538.

#### 8 (ref. 23)

Isolated as a colorless oil in 30% yield; ^1^H NMR (500 MHz, CDCl_3_) *δ* 7.43–7.34 (m, 4H), 7.27–7.20 (m, 2H), 7.12–7.06 (m, 4H), 2.70–2.59 (m, 4H), 1.95–1.84 (m, 4H); ^13^C NMR (126 MHz, CDCl_3_) *δ* 171.7, 150.6, 129.4, 125.8, 121.5, 34.0, 24.3.

### Periodic light ON/OFF switching experiment in the cobalt-catalysed Giese reaction (Fig. 1)

In a Schlenk tube equipped with a stirring bar, Mn powder (41.2 mg, 0.75 mmol) was added and heated at 400 °C for 3 min under vacuum. After cooling and filling with argon, the tube was charged with Et_3_N·HCl (51.6 mg, 0.38 mmol) and VB_12_ (16.9 mg, 12.5 μmol), evacuated and filled with argon for three times. After the addition of dry DMF (1.5 mL), trimethylsilyl chloride (*ca.* 6 μL), and mesitylene (12.0 mg, 0.15 mmol) as an internal standard (IS), the reaction mixture was stirred for 10 min. The tube was attached to an argon-filled balloon and moved to a light-tight room. Then, dodecyl tosylate (1a, 85.1 mg, 0.25 mmol) and methyl acrylate (2a, 32.3 mg, 0.38 mmol) were added into the solution. The light ON/OFF experiment was conducted under a periodic photo-irradiation with the photo-reaction apparatus mentioned above at the ambient temperature for 14 h. The product yield was determined by GC measurement of an aliquot of the reaction mixture in each reaction time. The Giese reaction under dark conditions was carried out in the same procedure.

### Time-dependent UV-Vis absorption spectra of Me-Cbl under the blue-light irradiation (Fig. 2)

The UV-Vis absorption spectra methylcobalamin (Me-Cbl) in DMF (10^−4^ mol L^−1^) were recorded on the Shimadzu UV-3600 spectrometer under argon atmosphere in a light-tight room, as depicted in Fig. S4.† Usually, cobalamin(iii) and (ii) species exhibit red and orange colors in solution, respectively.^6^ Before the blue-light irradiation (0 min), the solution showed red color and a strong absorption peak at 522 nm (red line). As the solution was exposed to the blue-light, the above-mentioned peak decreased and a new peak at 475 nm appeared. After the blue-light irradiation for 190 min, the color of the solution turned to orange. The results would indicate that the blue-light irradiation of Me-Cbl affords a Co(ii) complex with methyl radical through the cobalt–carbon bond cleavage.

### Radical trapping reaction with γ-terpinene (eqn (2) in Scheme 2)

In a Schlenk tube equipped with a stirring bar, Mn powder (41.2 mg, 0.75 mmol) was added and heated at 400 °C for 3 min under vacuum. After cooling and filling with argon, the tube was added vitamin B_12_ (VB_12_; 16.9 mg, 12.5 μmol), evacuated and filled with argon three times. After adding dry DMF (1.5 mL) and trimethylsilyl chloride (*ca.* 6 μL), the mixture was stirred for 5 min. Then, dodecyl tosylate (1a, 85.1 mg, 0.25 mmol) was added to the solution. The reaction mixture was stirred at room temperature for 16 h under blue-light irradiation. The obtained mixture was diluted with ethyl acetate and quenched by saturated aqueous NH_4_Cl. The product 7 was characterised by an authentic sample. The yield was evaluated by GC measurement using mesitylene as an internal standard.

## Conflicts of interest

There are no conflicts to declare.

## Supplementary Material

RA-011-D0RA10739E-s001
