# Supplementary material for: Visible light-driven Giese reaction with alkyl tosylates catalysed by nucleophilic cobalt
Source: RSC Adv. 2021 Jan 18;11(6):3539–46. doi: 10.1039/d0ra10739e (PMC8693991; doi:10.1039/d0ra10739e)

Electronic Supplementary Material (ESI) for RSC Advances.
This journal is © The Royal Society of Chemistry 2021

Electronic Supplementary Information for

Visible light-driven Giese reaction with alkyl tosylates catalysed by nucleophilic cobalt

Kimihiro Komeyama,* Takuya Michiyuki, Yoshikazu Teshima, and Itaru Osaka

Department of Applied Chemistry, Graduate School of Advanced Science and Engineering, Hiroshima University
1-4-1 Kagamiyama, Higashi-Hiroshima City, Hiroshima 739-8527, Japan
Email: kkome@hiroshima-u.ac.jp

1. General information

All reactions were performed on oven-and flame-dried glassware under argon using standard Schlenk techniques. Flash-column chromatography was performed with silica-gel 60 (KANTO Chemical Co. Inc., 40–50 nm). TLC monitoring was carried out with silica-gel aluminum sheets (Merck, type 60 F_{254}). Gas chromatography (GC) monitoring was carried out on Shimadzu GC-2014. Nuclear magnetic resonance (NMR) spectra were recorded with Varian-400 (^{1}H NMR: 400 MHz; ^{13}C NMR: 101 MHz) spectrometer or Varian-500 (^{1}H NMR: 500 MHz; ^{13}C NMR: 126 MHz) spectrometers calibrated from residual deuterated chloroform as an internal standard at 7.26 ppm for ^{1}H NMR spectra and at 77.0 ppm for ^{13}C NMR spectra, respectively. High-resolution mass spectrum (HRMS) was performed by the Natural Science Centre for Basic Research and Development (N-BARD) of Hiroshima University using LTQ Orbitrap XL from Thermo Fisher Scientific.

2. Materials

Cobalt complexes and manganese power were purchased from Nacalai Tesque, Inc. [for VB_{12} and Mn] and Tokyo Chemical Industry Co., Ltd. [for Co(salen), Co(Pc), Co(TMPP), and methylcobalamin hydrate (Me-Cbl)], respectively and were used without purifications. $CoCl(dmgH)_2Py$ was prepared according to literature.[1] Alkyl tosylates[2,3] and activated olefins[4,5] were prepared on the basis of reported methods. DMF was dried over activated MS 4Å, distilled, and stored with activated MS 4Å under argon. Unless otherwise noted, commercially available reagents were used as received without further purification.

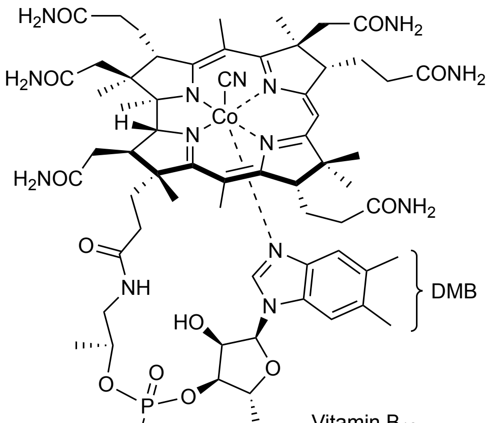

Vitamin B_{12} (VB_{12})

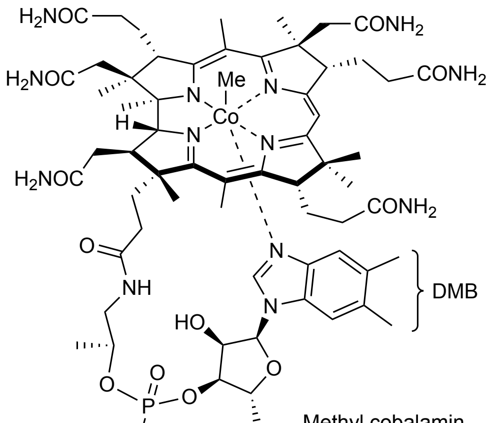

Methyl cobalamin (Me-Cbl)

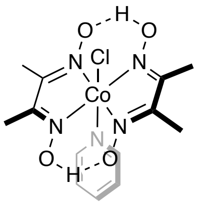

$CoCl(dmgH)_2Py$

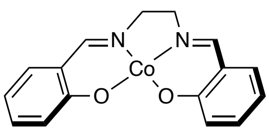

Co(salen)

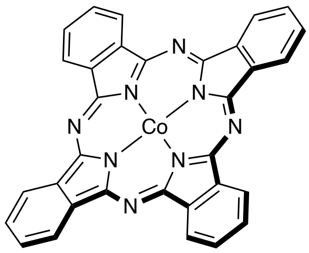

Co(Pc)

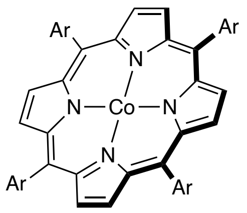

Co(TMPP)
Ar = 4-$MeOC_6H_4$

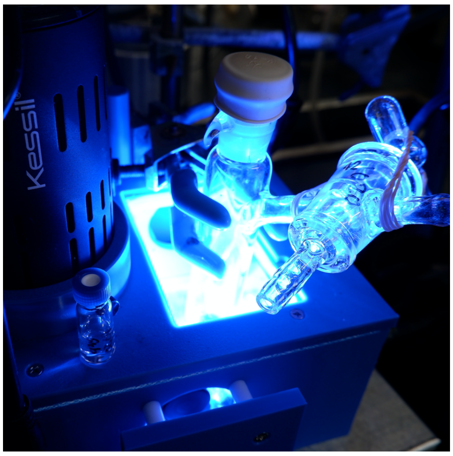

Figure S1. Reaction setup for the nucleophilic cobalt-catalysed Giese reaction under blue-light irradiation.

(a) (b) (c) (d)

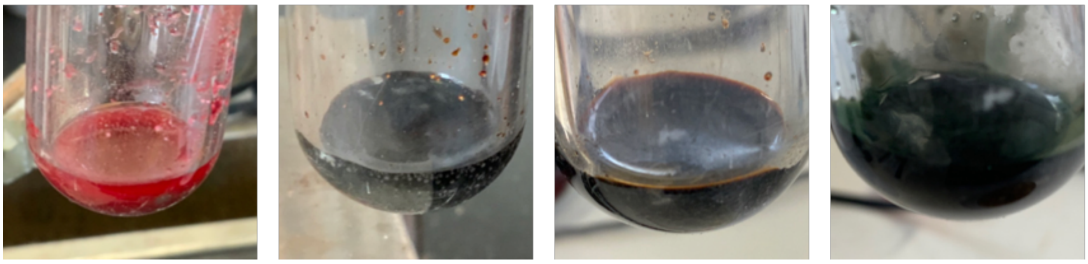

Figure S2. The color changes of the reaction mixture in each step: (a) Mn + Et_3N·HCl + VB_{12} + DMF; (b) Solution a + TMSCl (*ca.* 6 μL); (c) After stirring for 8 h with photo-irradiation; (d) After stirring for 16 h with photo-irradiation.

References

1 A. Panagiotopoulos, K. Ladomenou, D. Sun, V. Artero and A. G. Coutsolelos, *Dalton Trans.*, 2016, **45**, 6732–6738.

2 T. Michiyuki, I. Osaka and K. Komeyama, *Chem. Commun.*, 2020, **56**, 1247–1250.

3 K. Tani and B. M. Stoltz, *Nature*, 2006, **441**, 731–734.

4 S. Eggers, T. Eckert and V. Abetz, *J. Polym. Sci., Part A: Polym. Chem.*, 2018, **56**, 399–411.

5 S. M. Hell, C. F. Meyer, G. Laudadio, A. Misale, M. C. Willis, T. Noël, A. A. Trabanco and V. Gouverneur, *J. Am. Chem. Soc.*, 2020, **142**, 720–725.

863_1H NMR (CDCl3, 499.94 MHz)

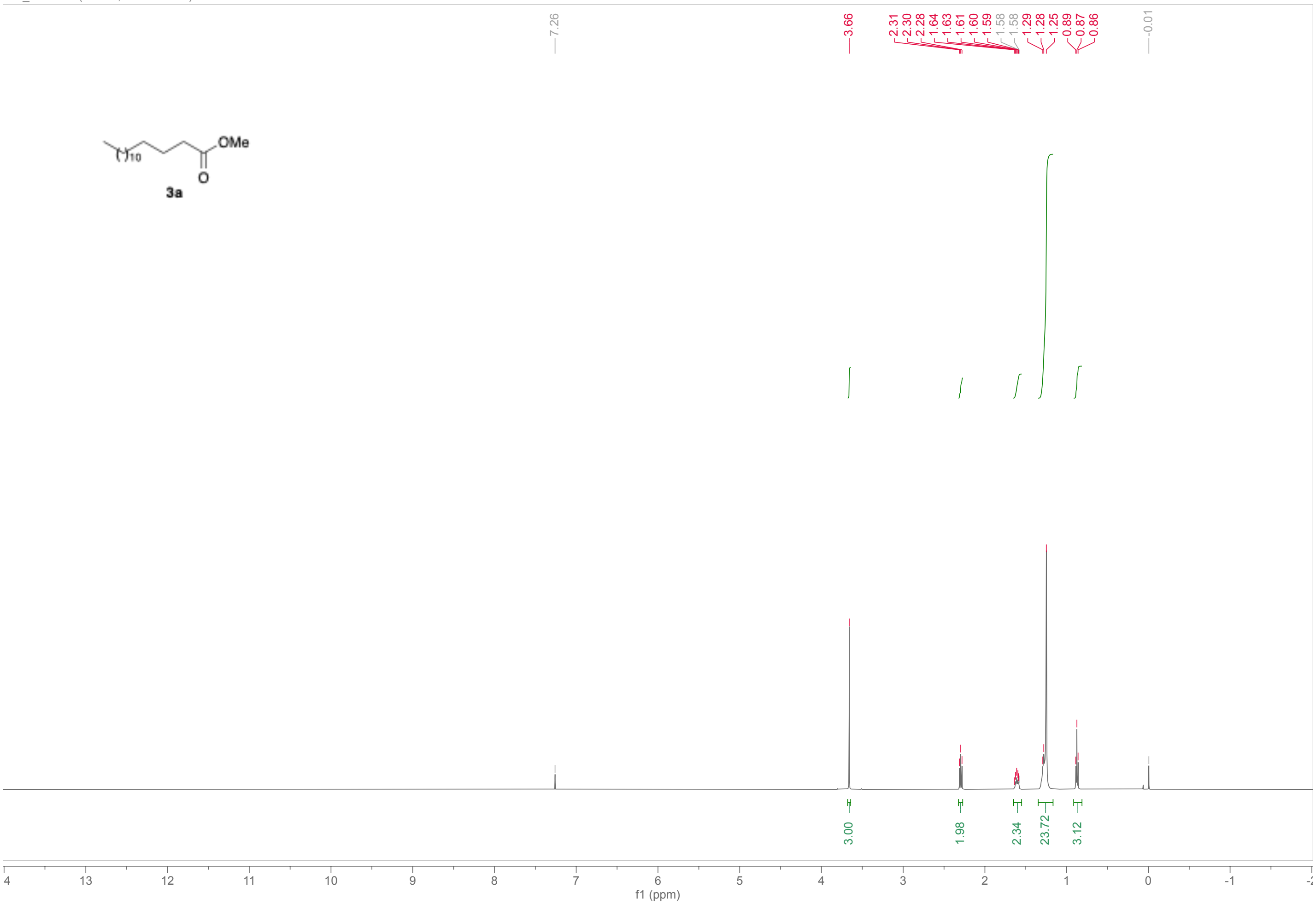

863_13C NMR (CDCl3, 125.72 MHz)

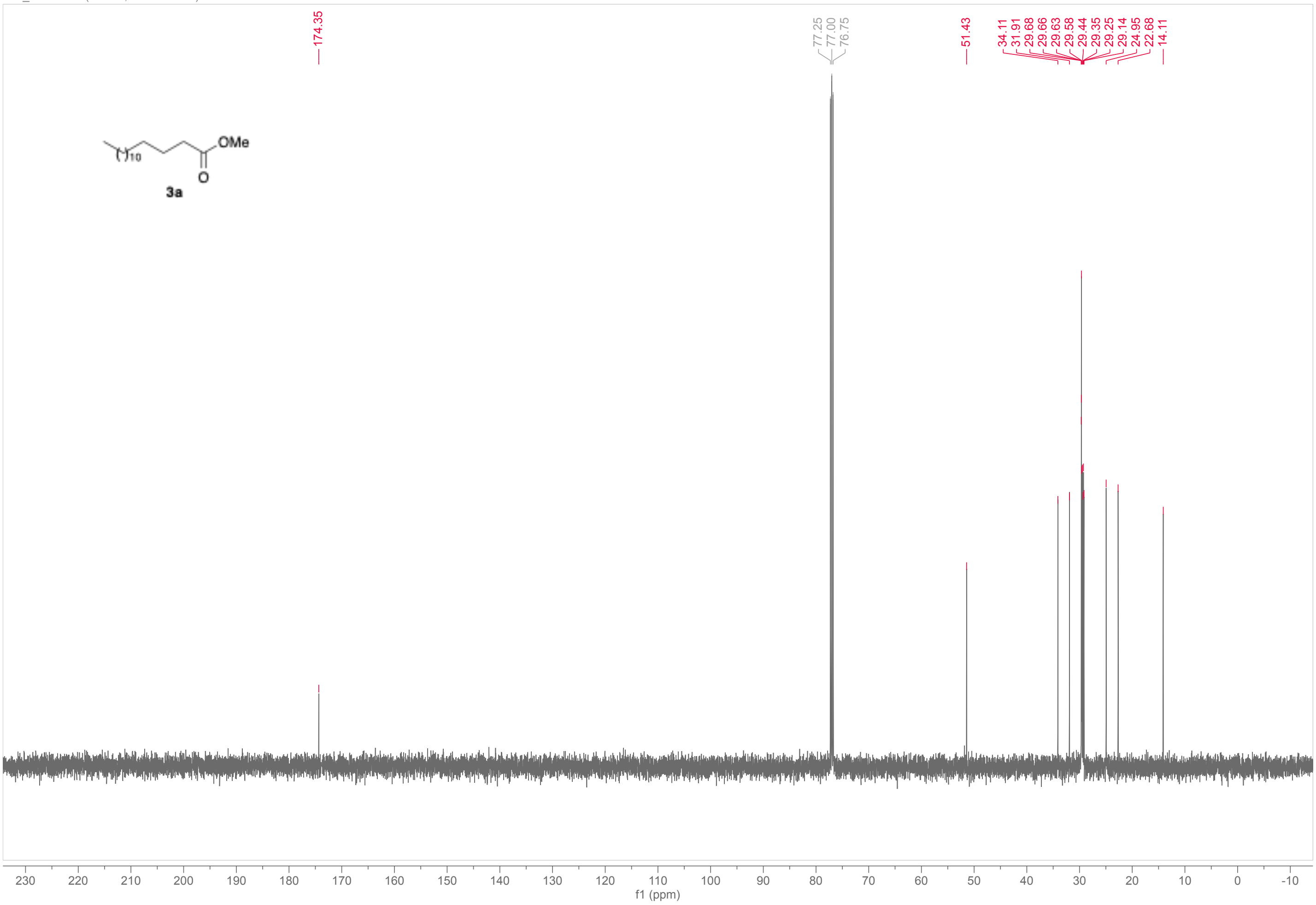

875_1H NMR (CDCl3, 499.94 MHz)

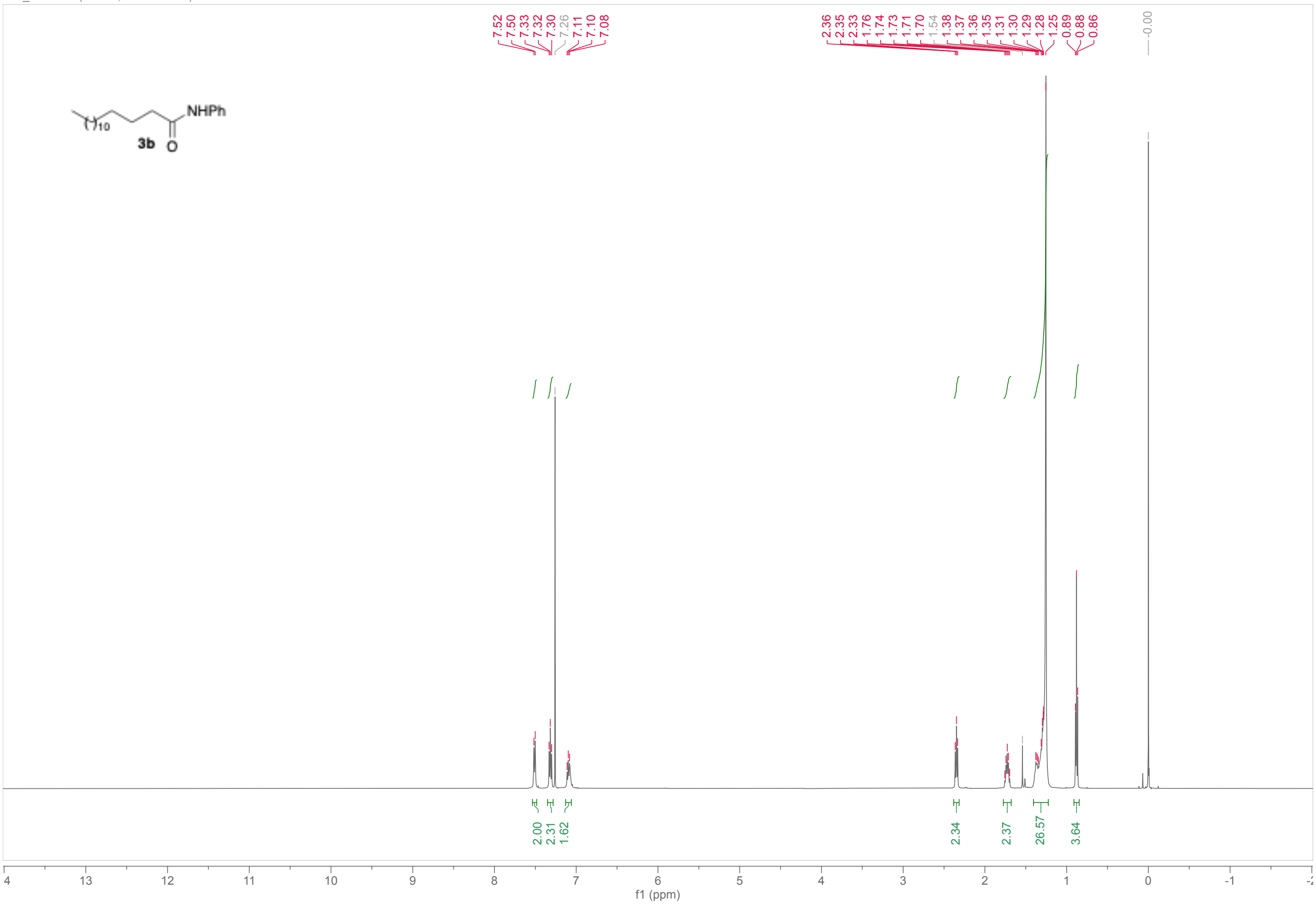

875_13C NMR (CDCl3, 125.72 MHz)

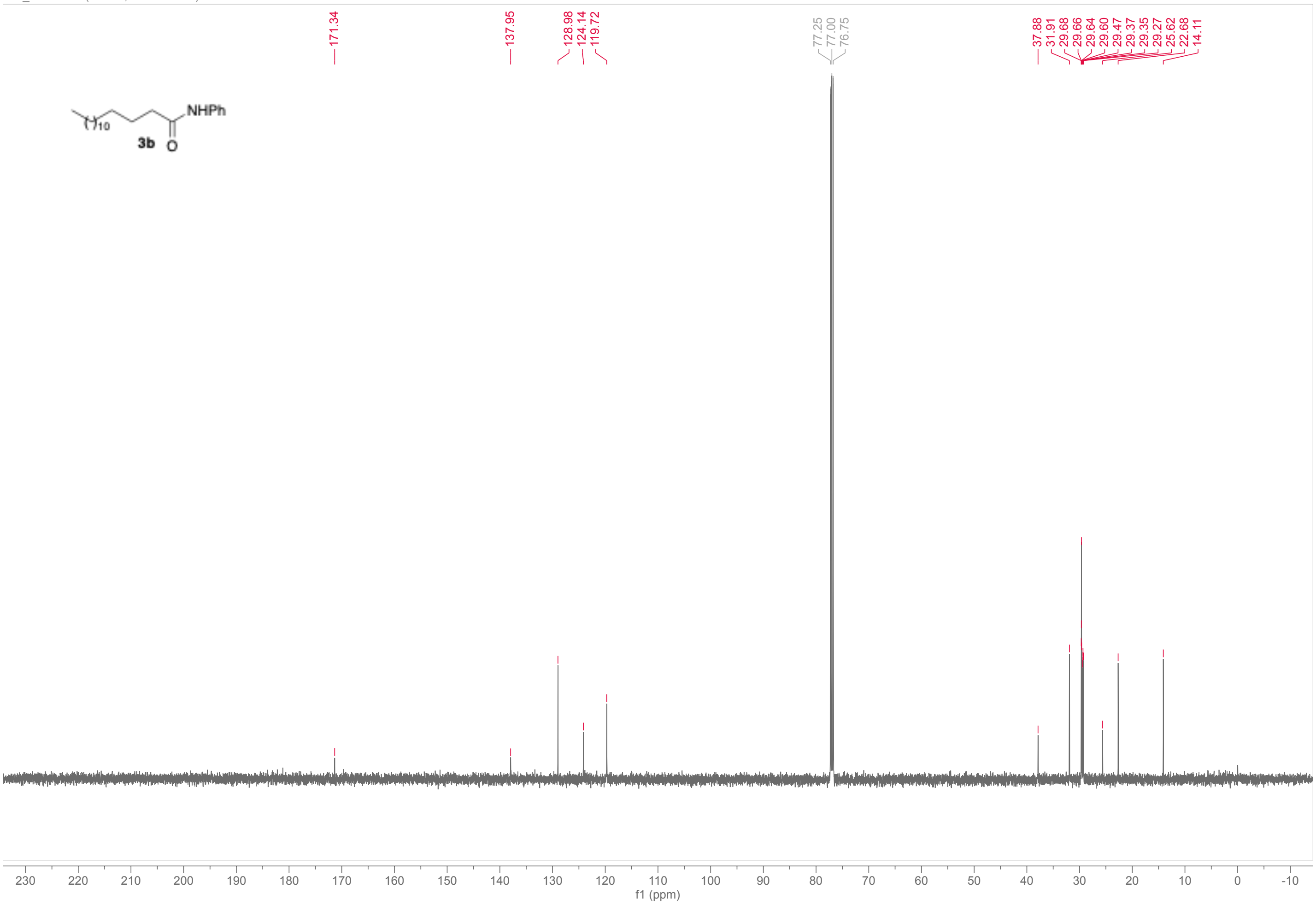

877_1H NMR (CDCl3, 500.00 MHz)

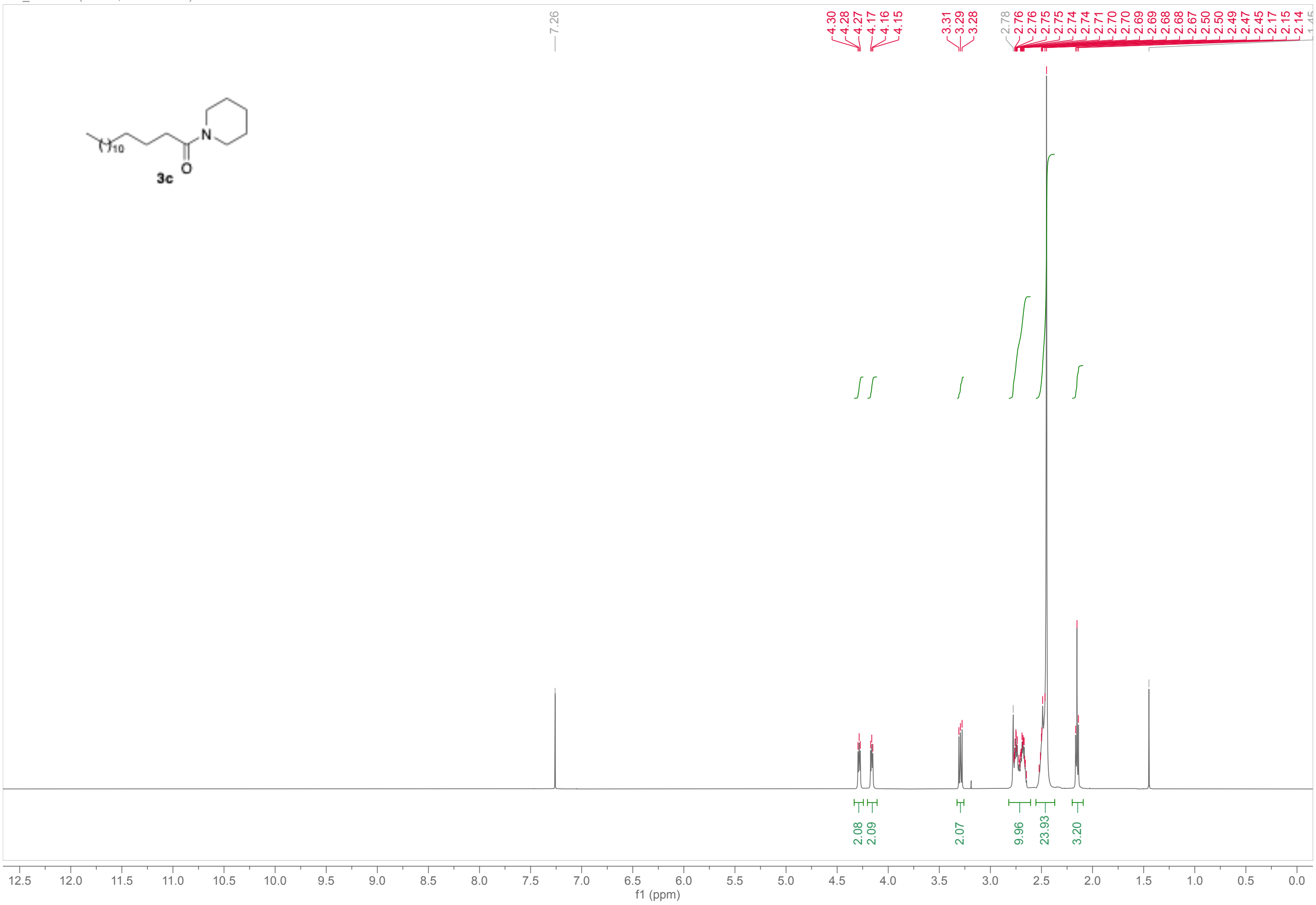

877_13C NMR (CDCl3, 125.72 MHz)

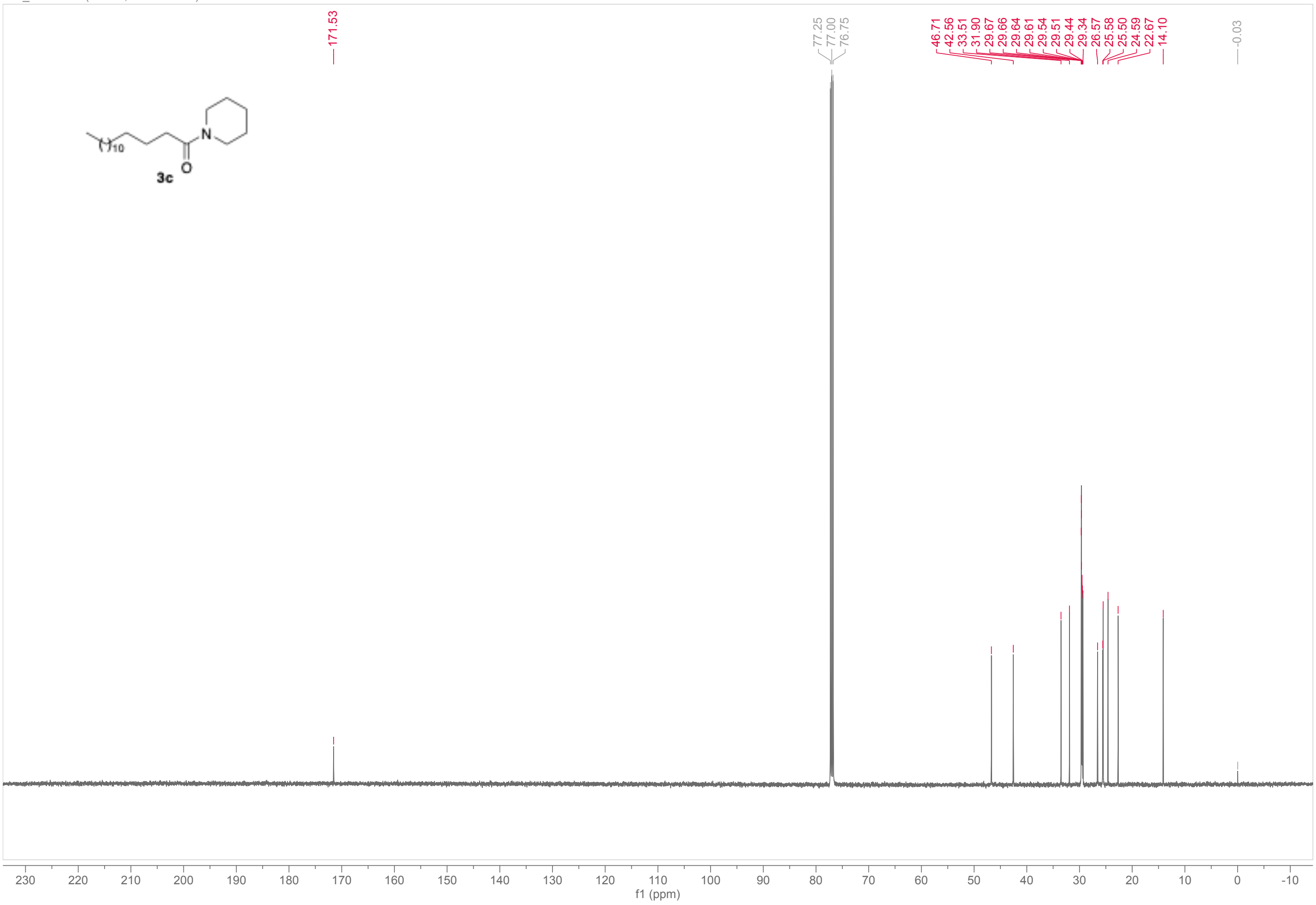

1011_1H NMR (CDCl3, 499.94 MHz)

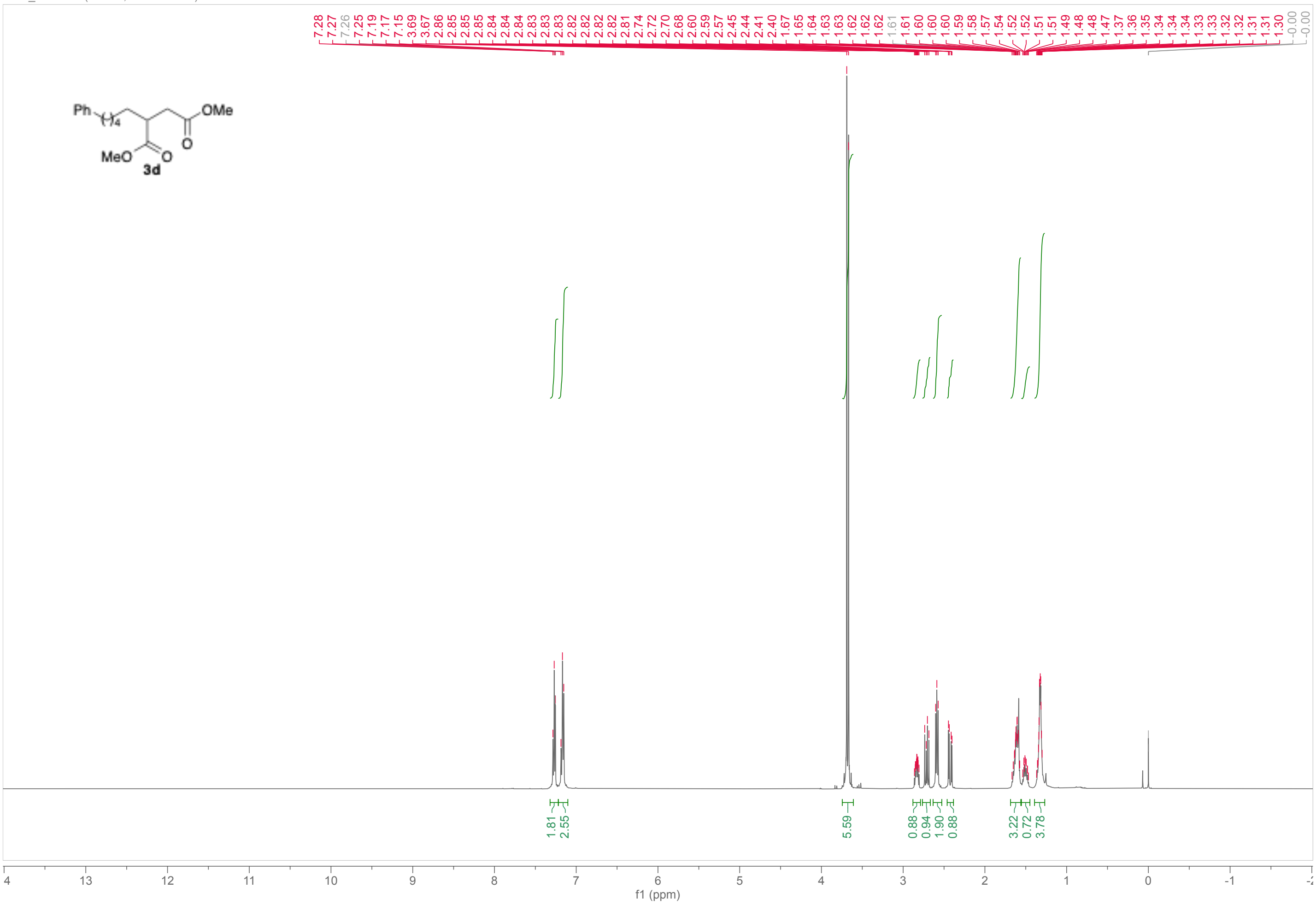

1011_13C NMR (CDCl3, 125.72 MHz)

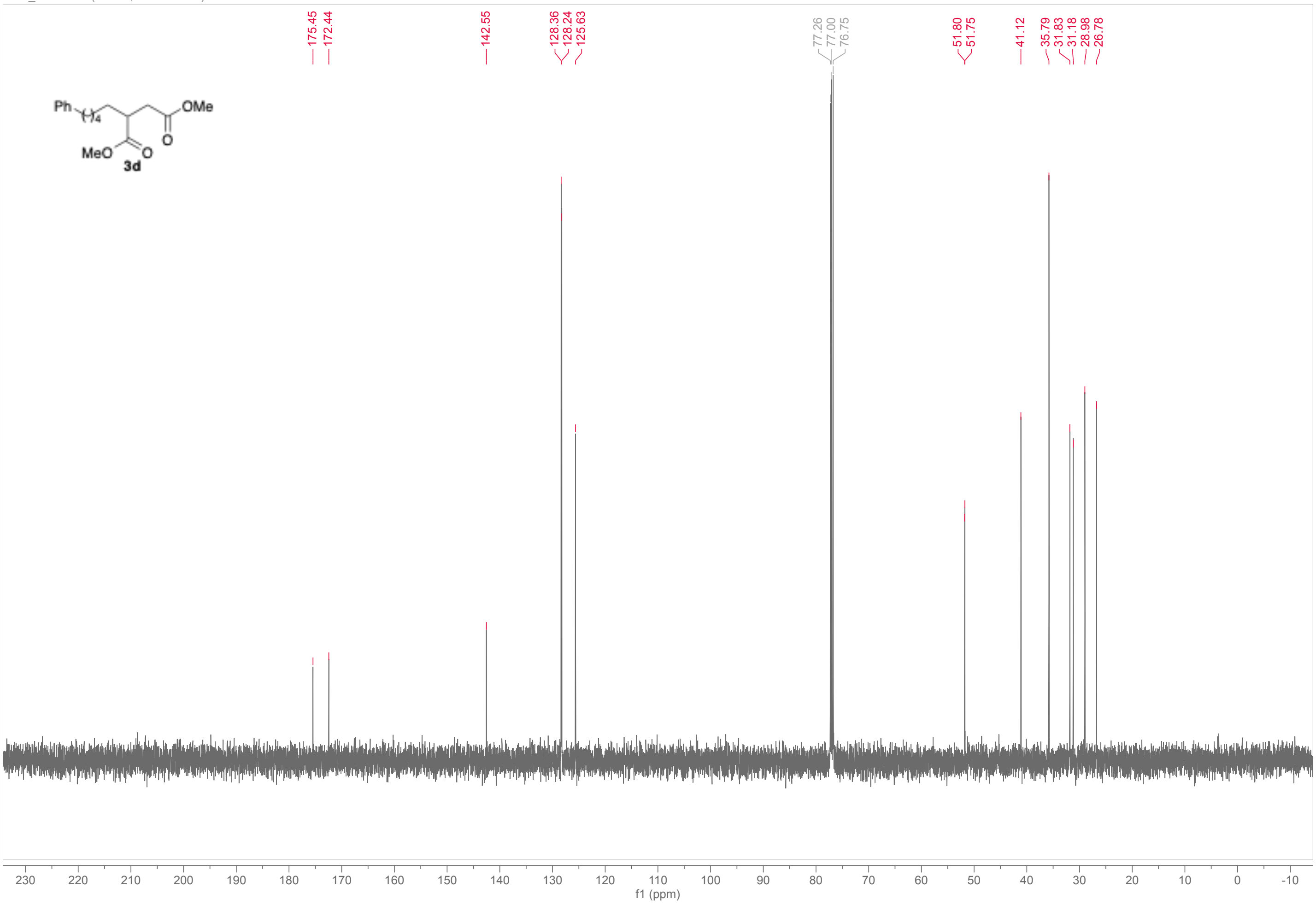

1018_1H NMR (CDCl3, 499.94 MHz)

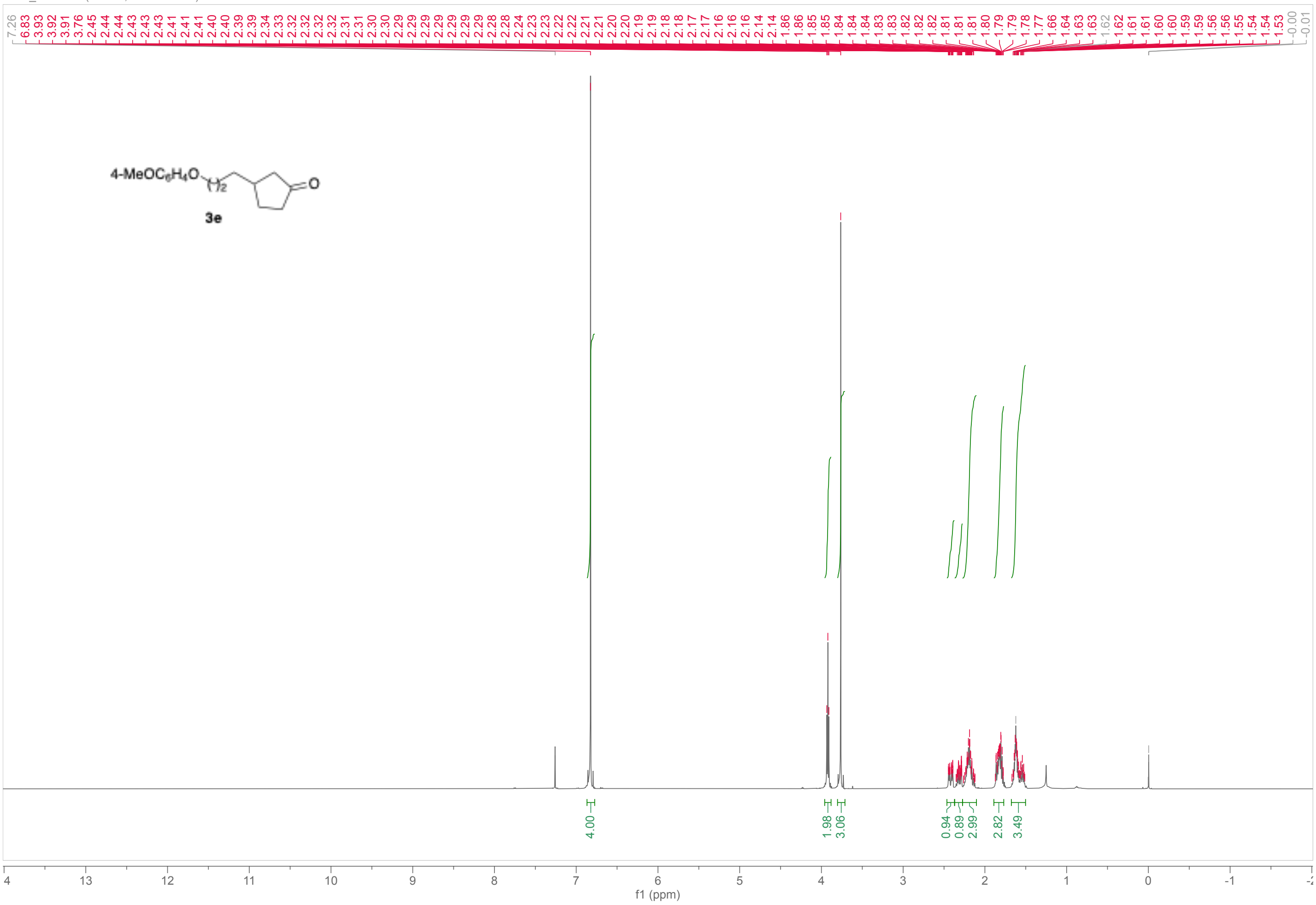

1018_13C NMR (CDCl3, 125.72 MHz)

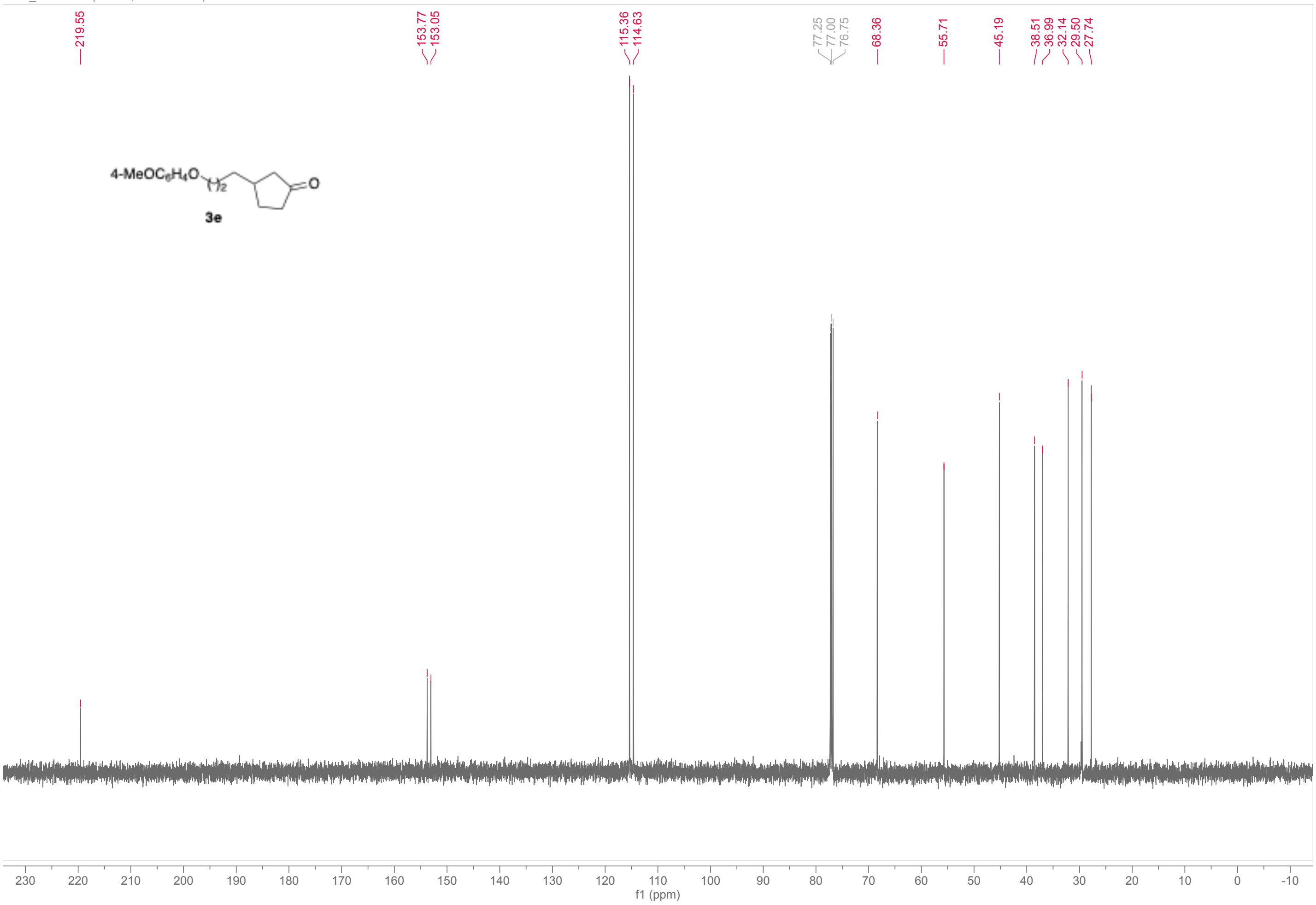

1007_1H NMR (CDCl3, 499.94 MHz)

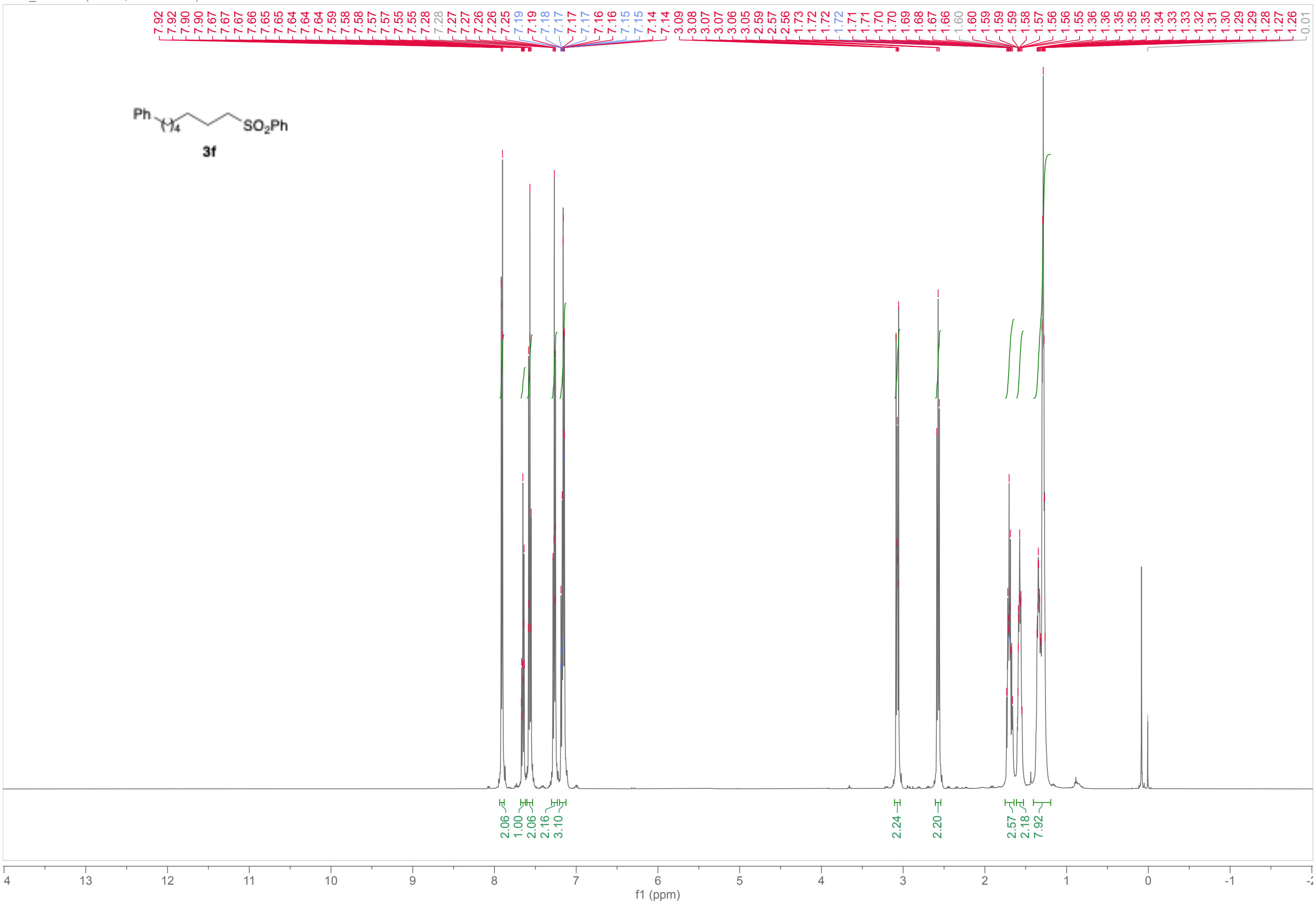

1007_13C NMR (CDCl3, 125.72 MHz)

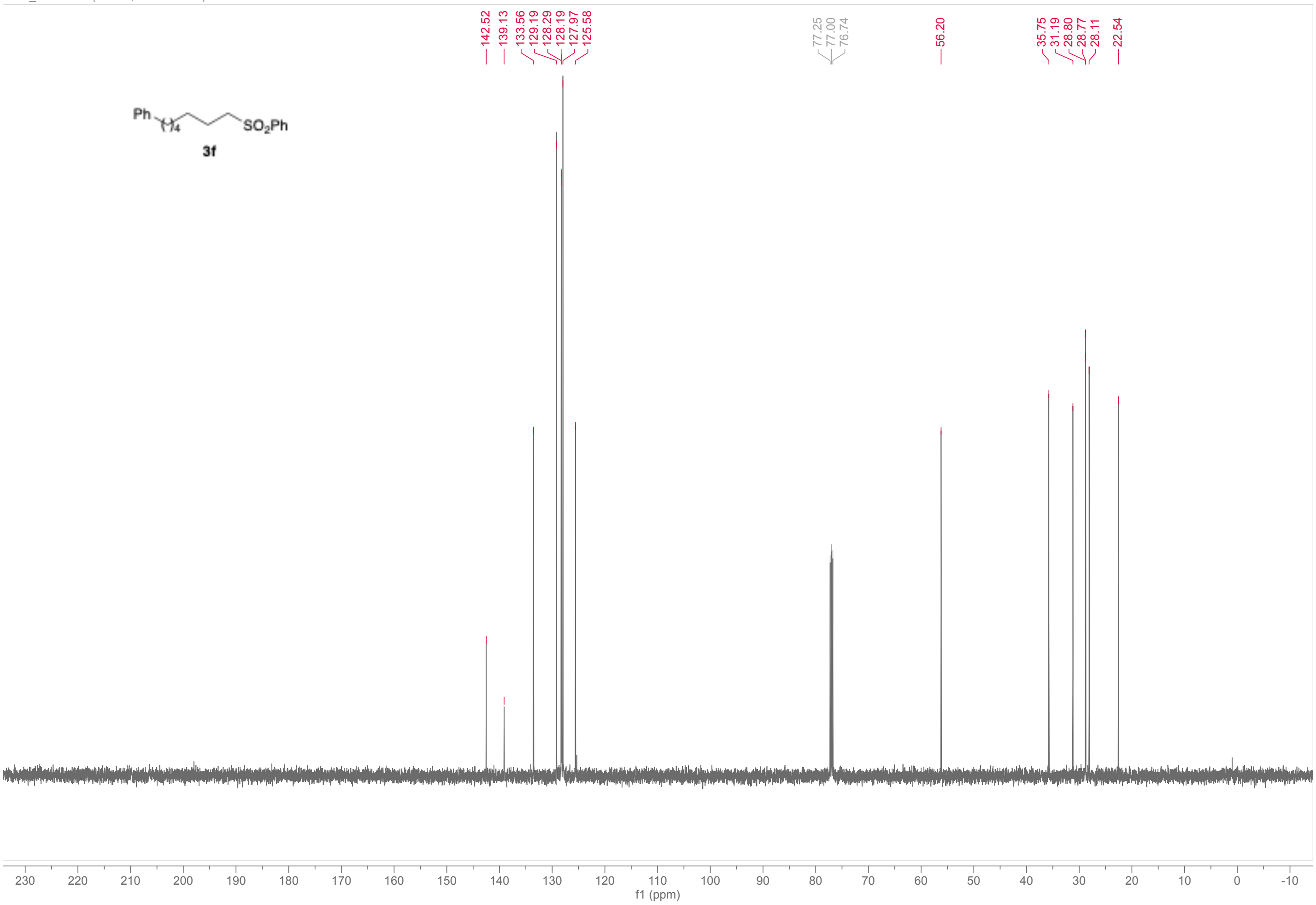

1022_1H NMR (CDCl3, 499.94 MHz)

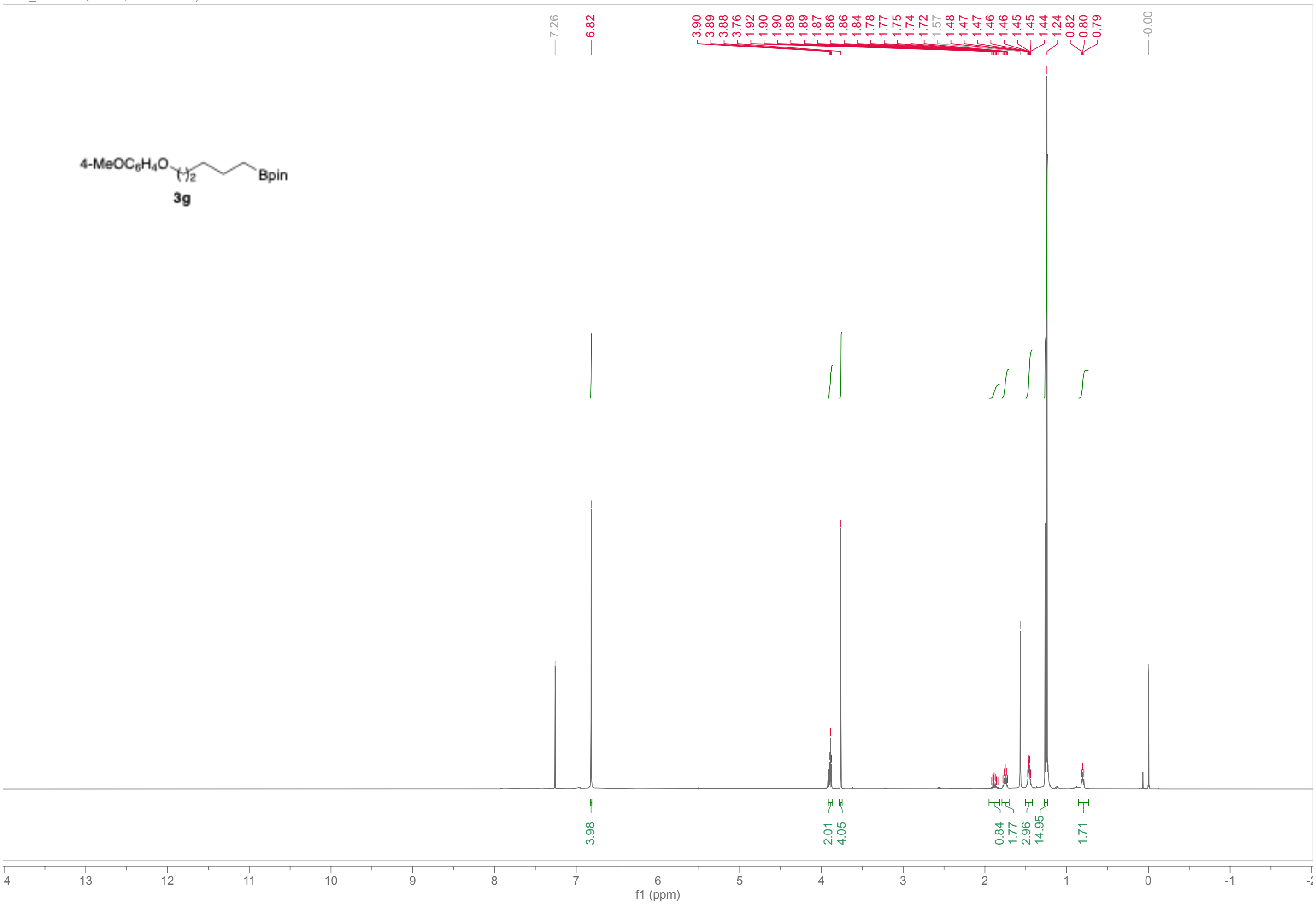

1022_13C NMR (CDCl3, 125.72 MHz)

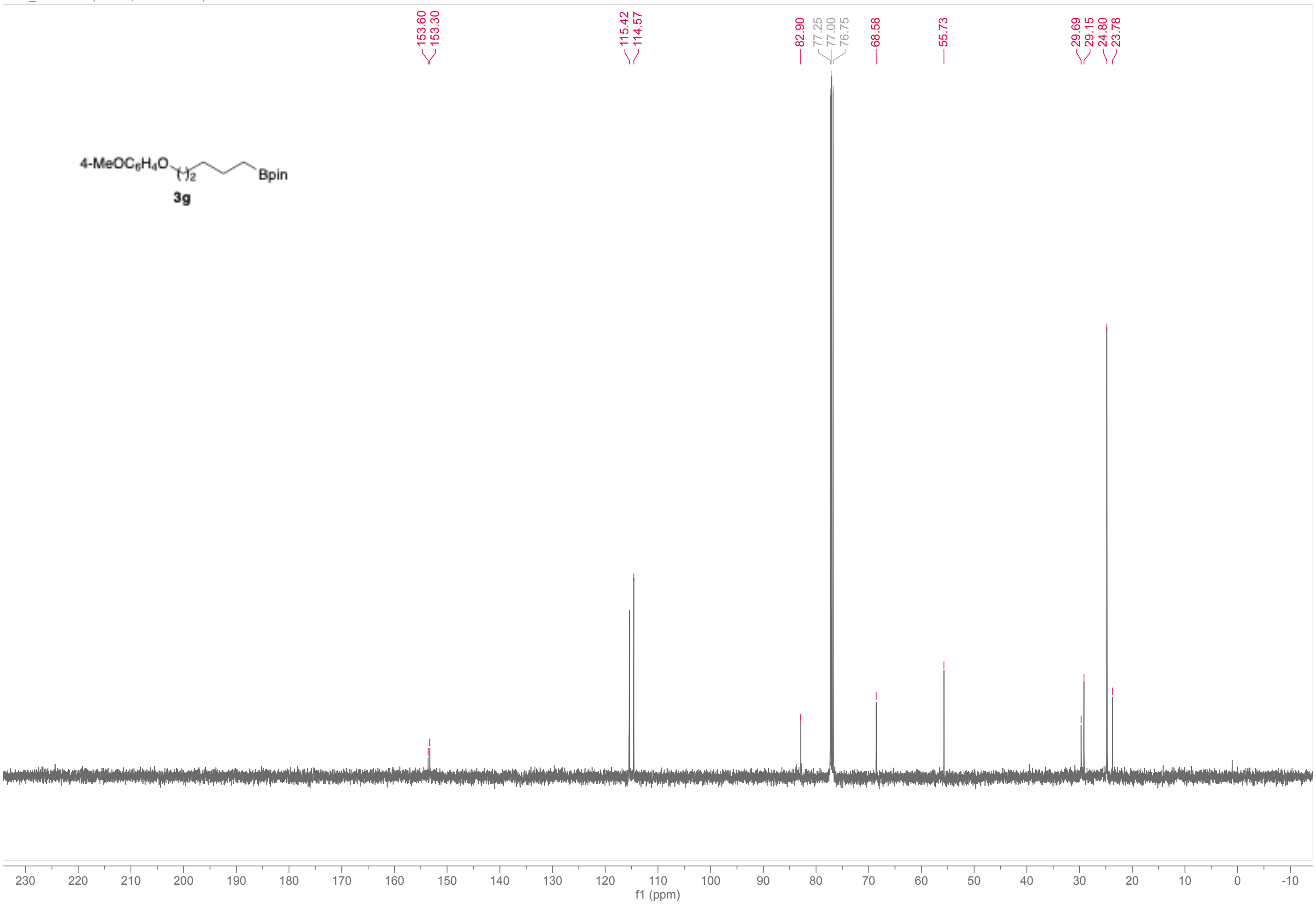

1006_1H NMR (CDCl3, 499.94 MHz at 35.0 °C)

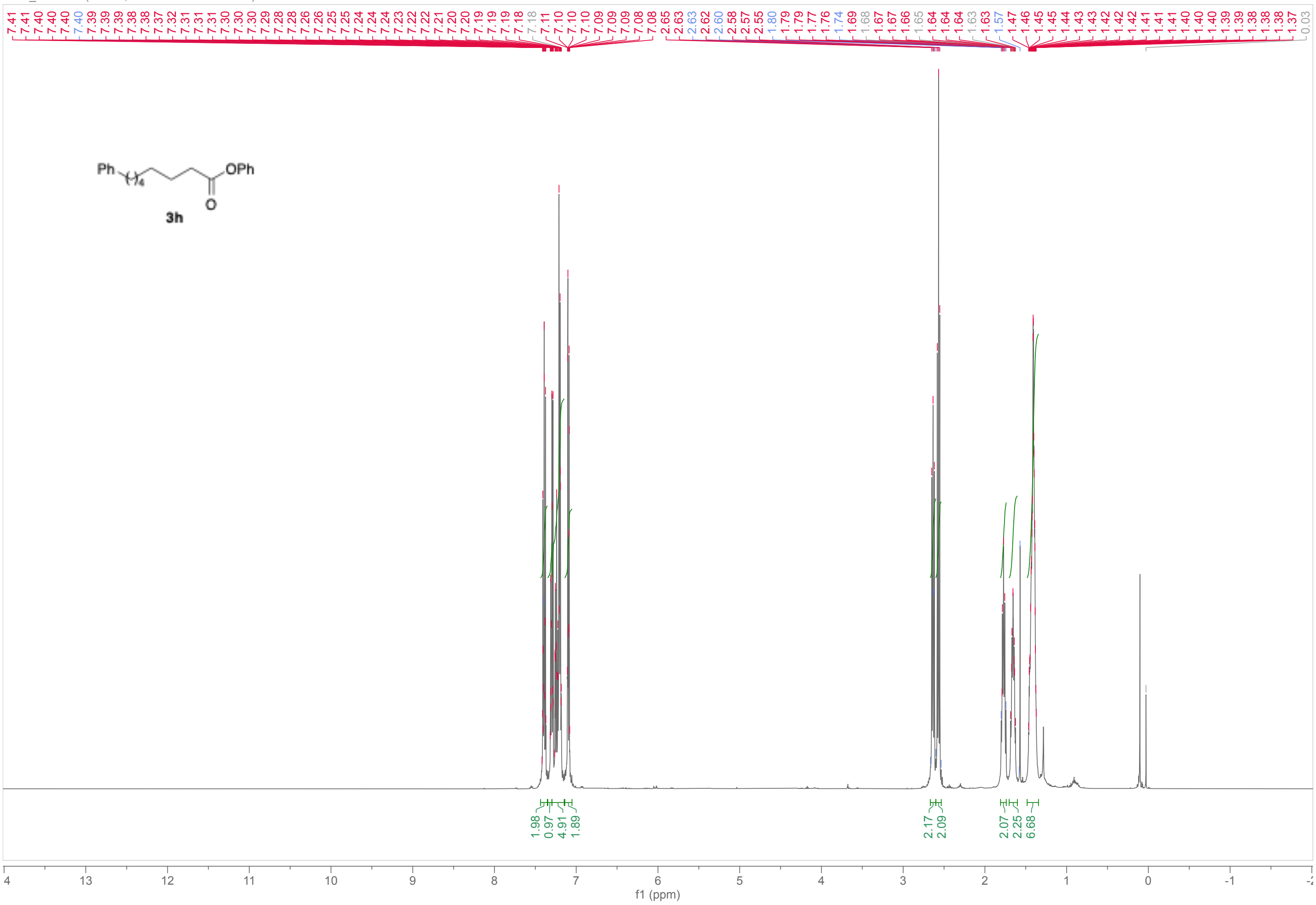

1006_13C NMR (CDCl3, 125.72 MHz)

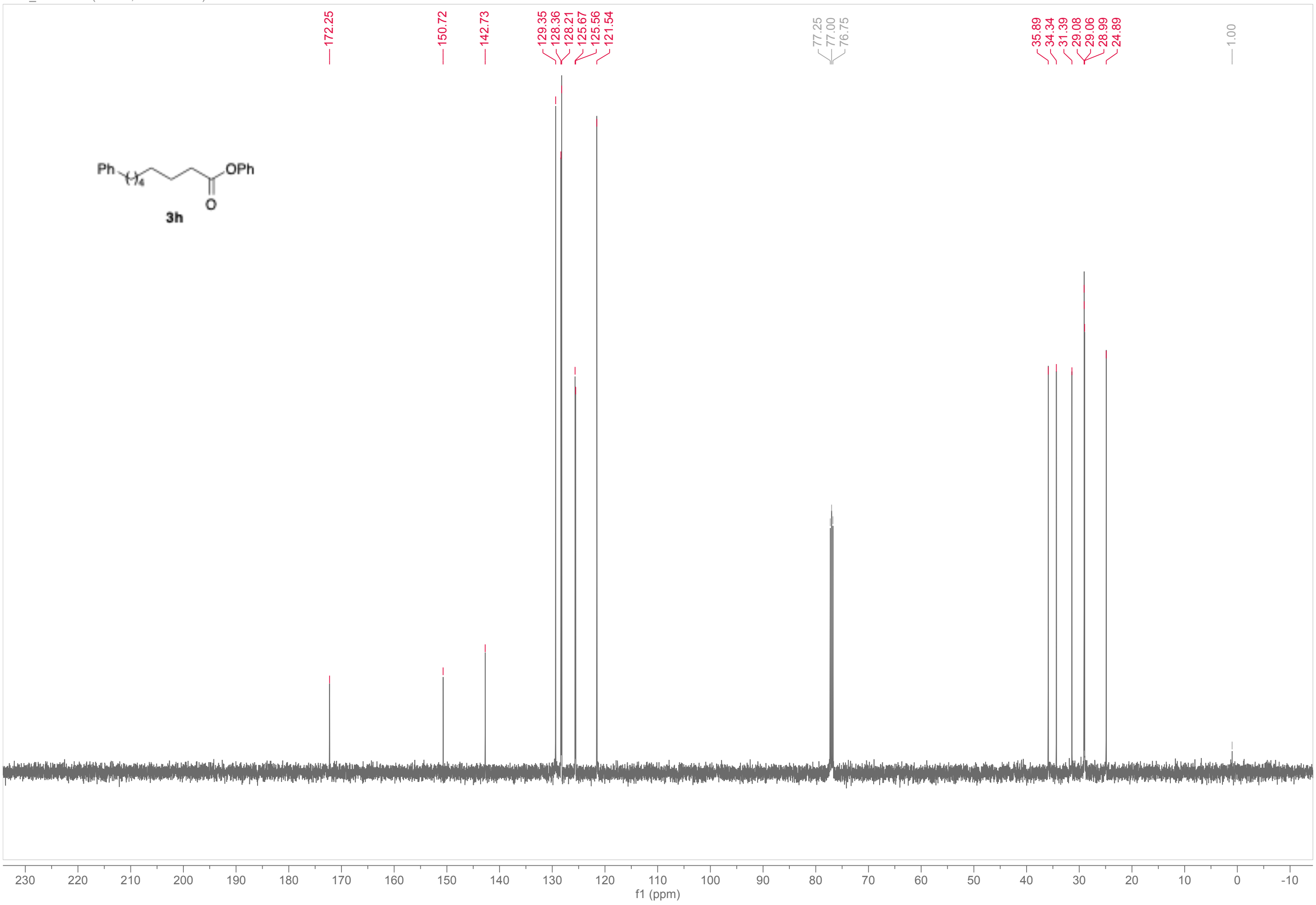

954_1H NMR (CDCl3, 499.94 MHz)

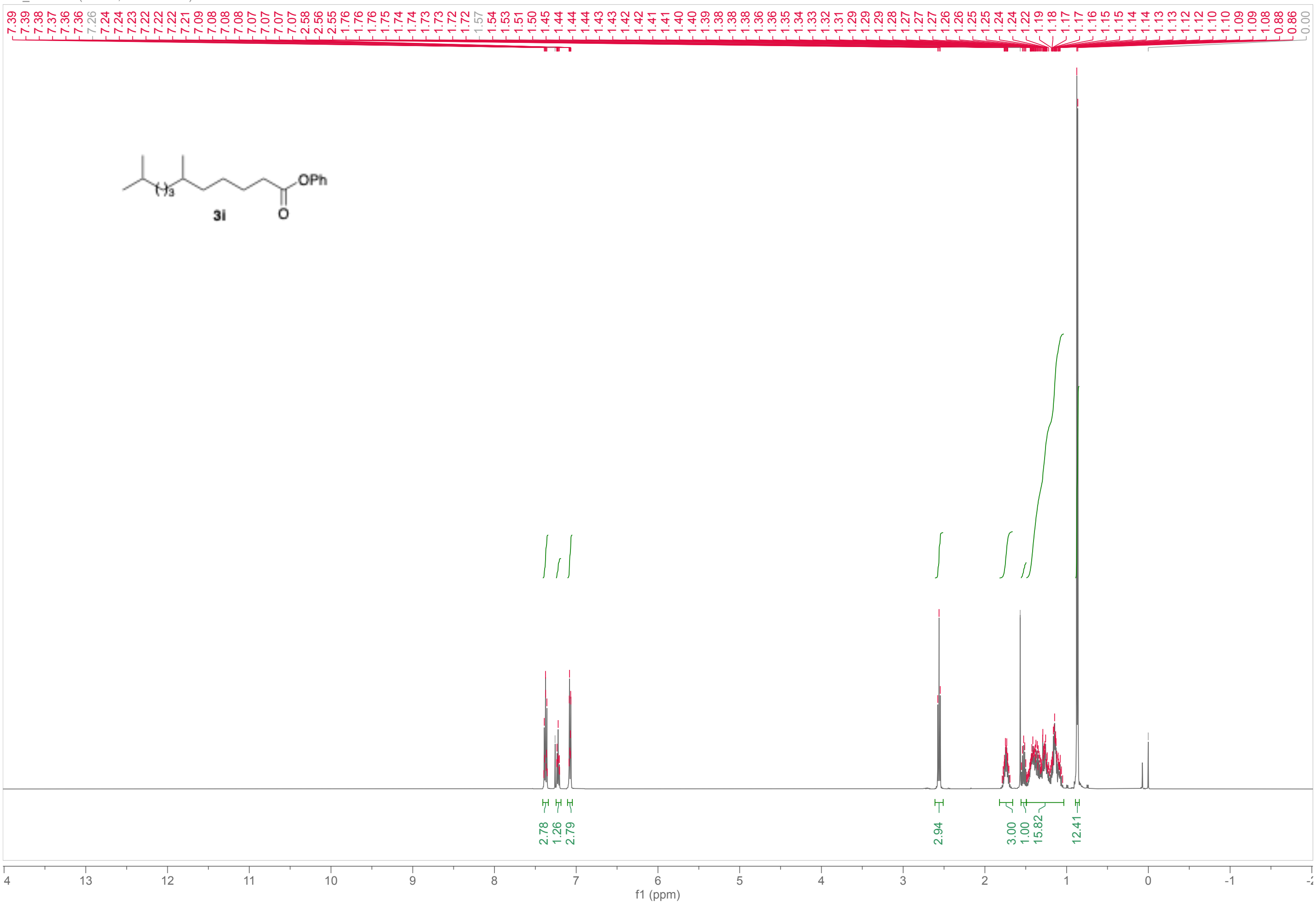

954_13C NMR (CDCl3, 125.72 MHz)

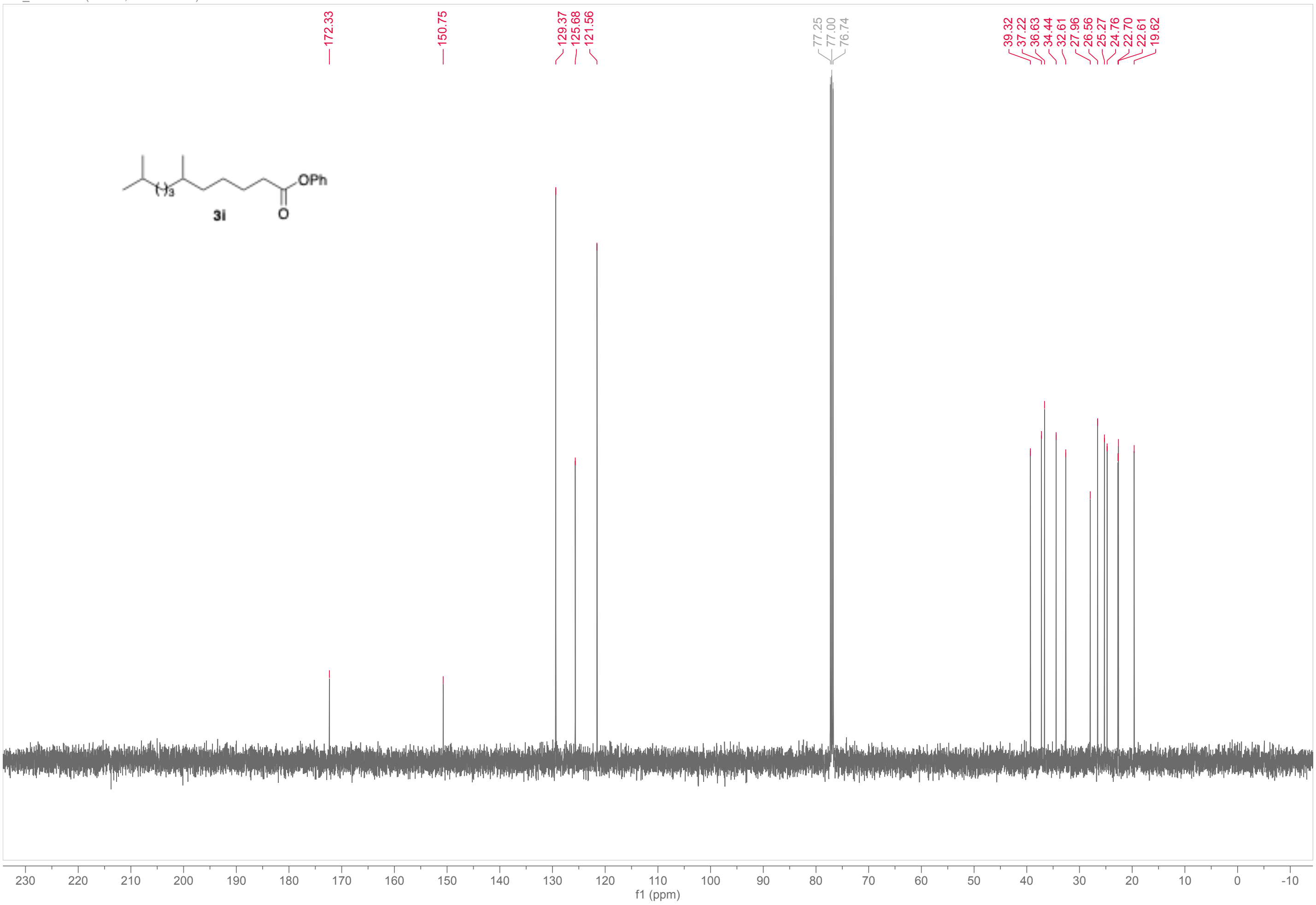

913_1H NMR (CDCl3, 499.94 MHz)

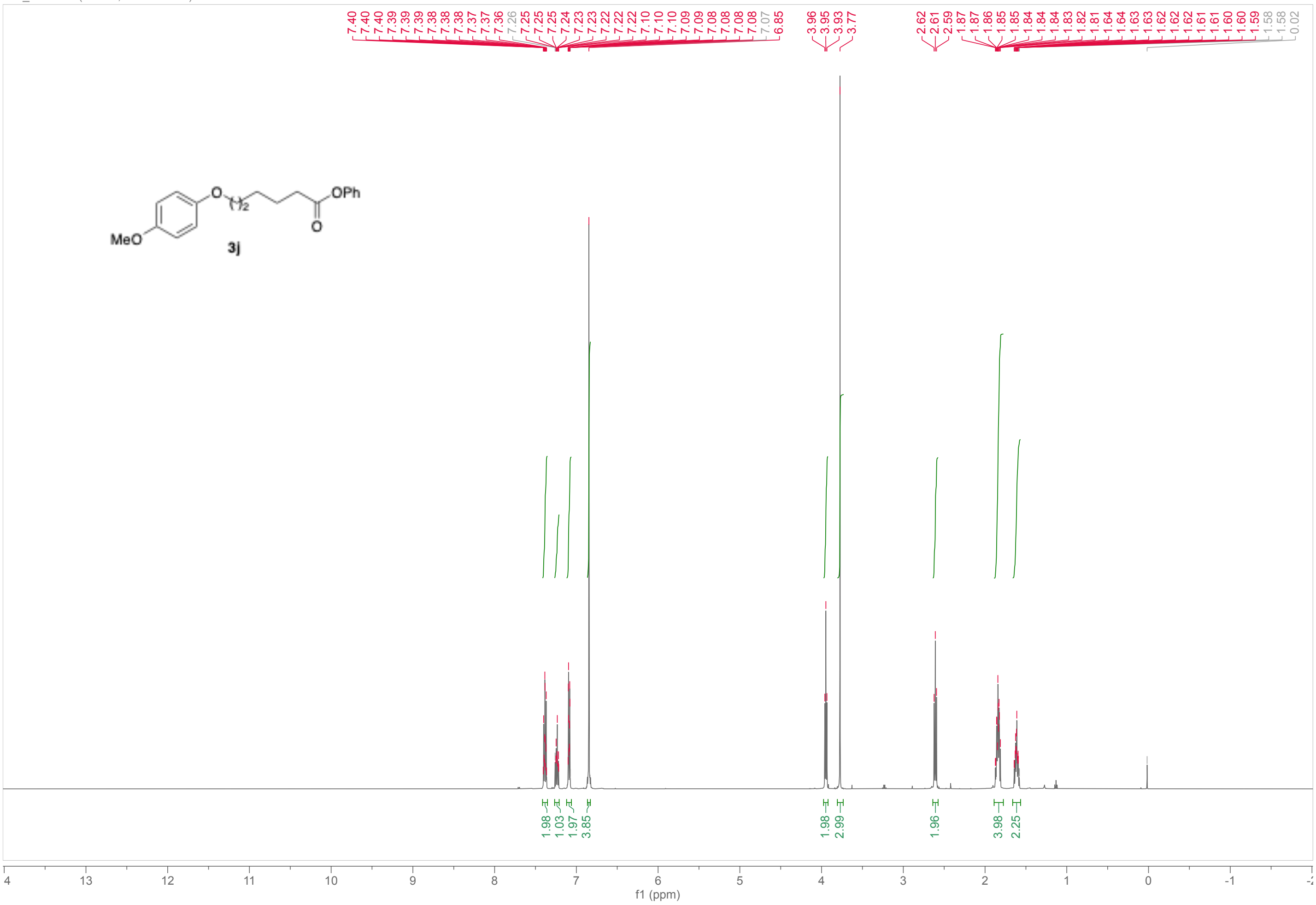

913_13C NMR (CDCl3, 125.72 MHz)

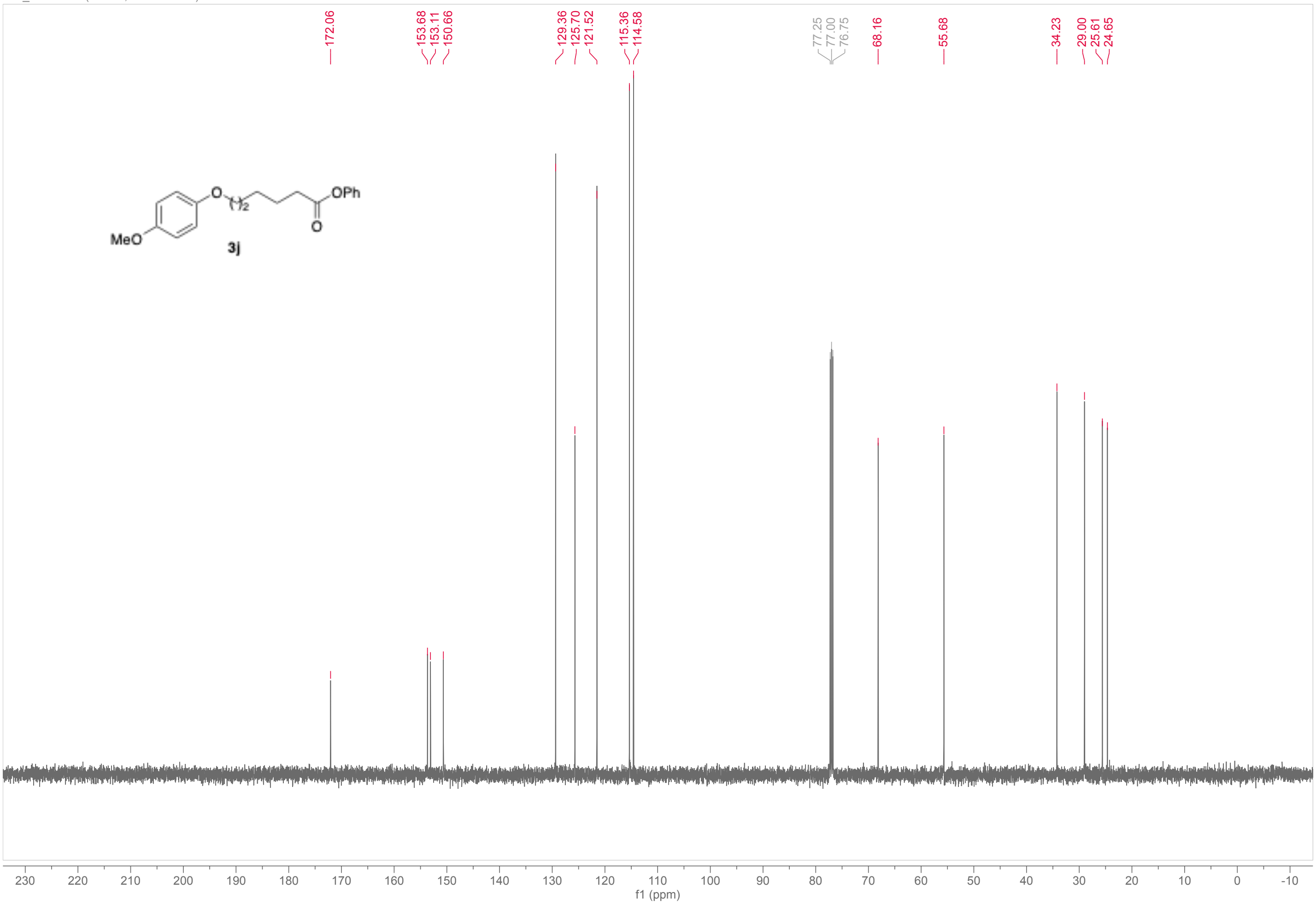

926_1H NMR (CDCl3, 499.94 MHz)

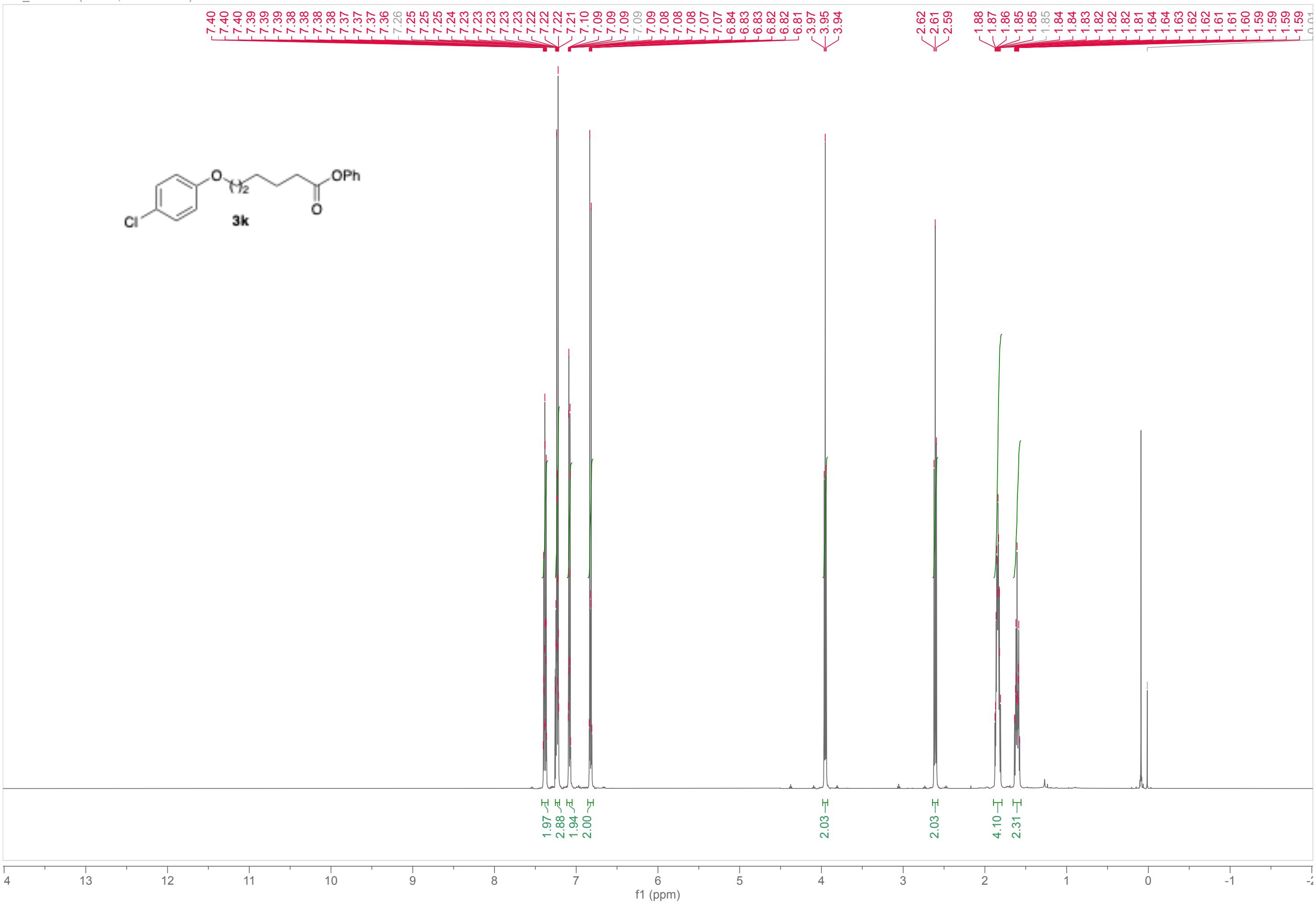

926_13C NMR (CDCl3, 125.72 MHz)

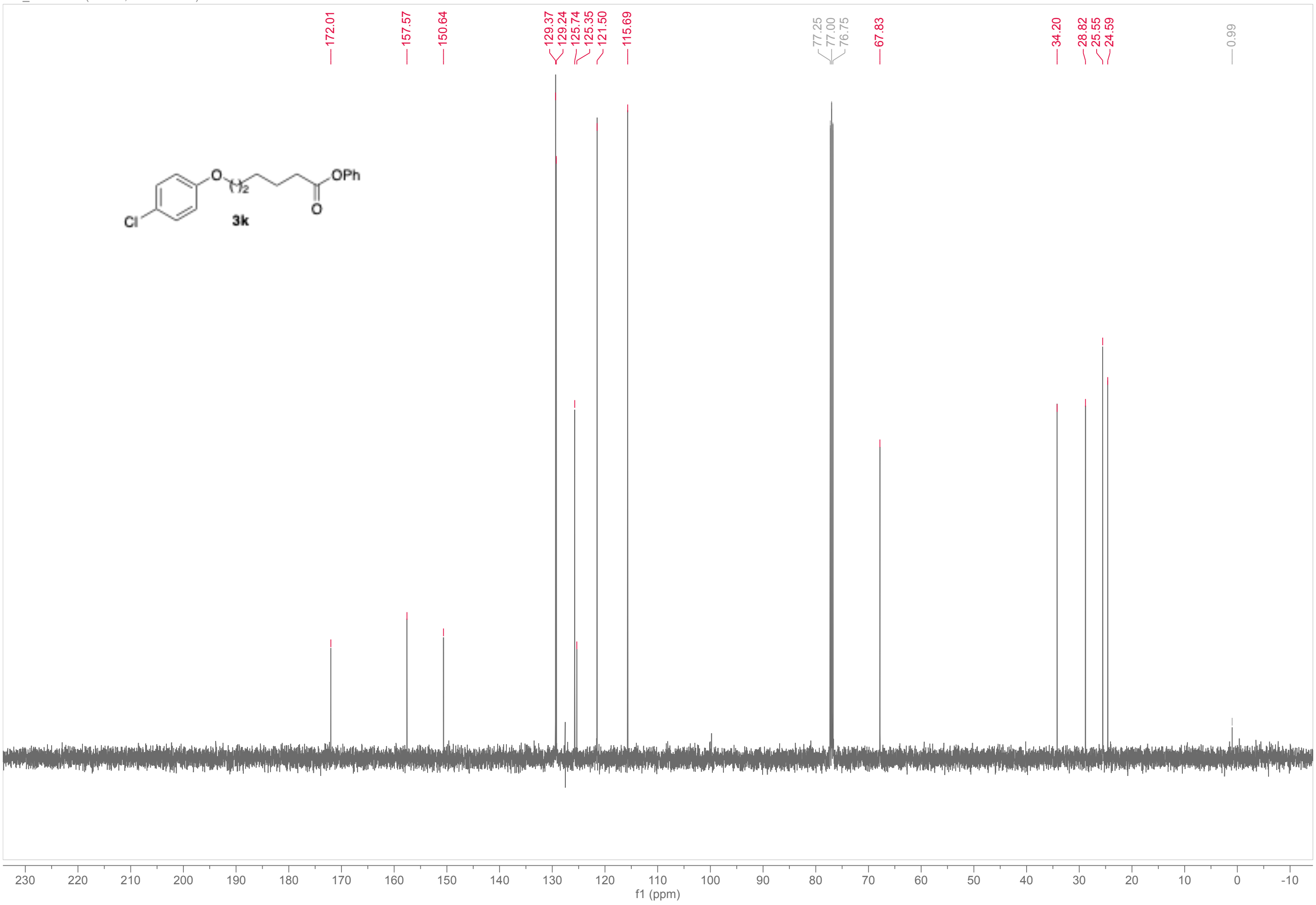

925_1H NMR (CDCl3, 499.94 MHz)

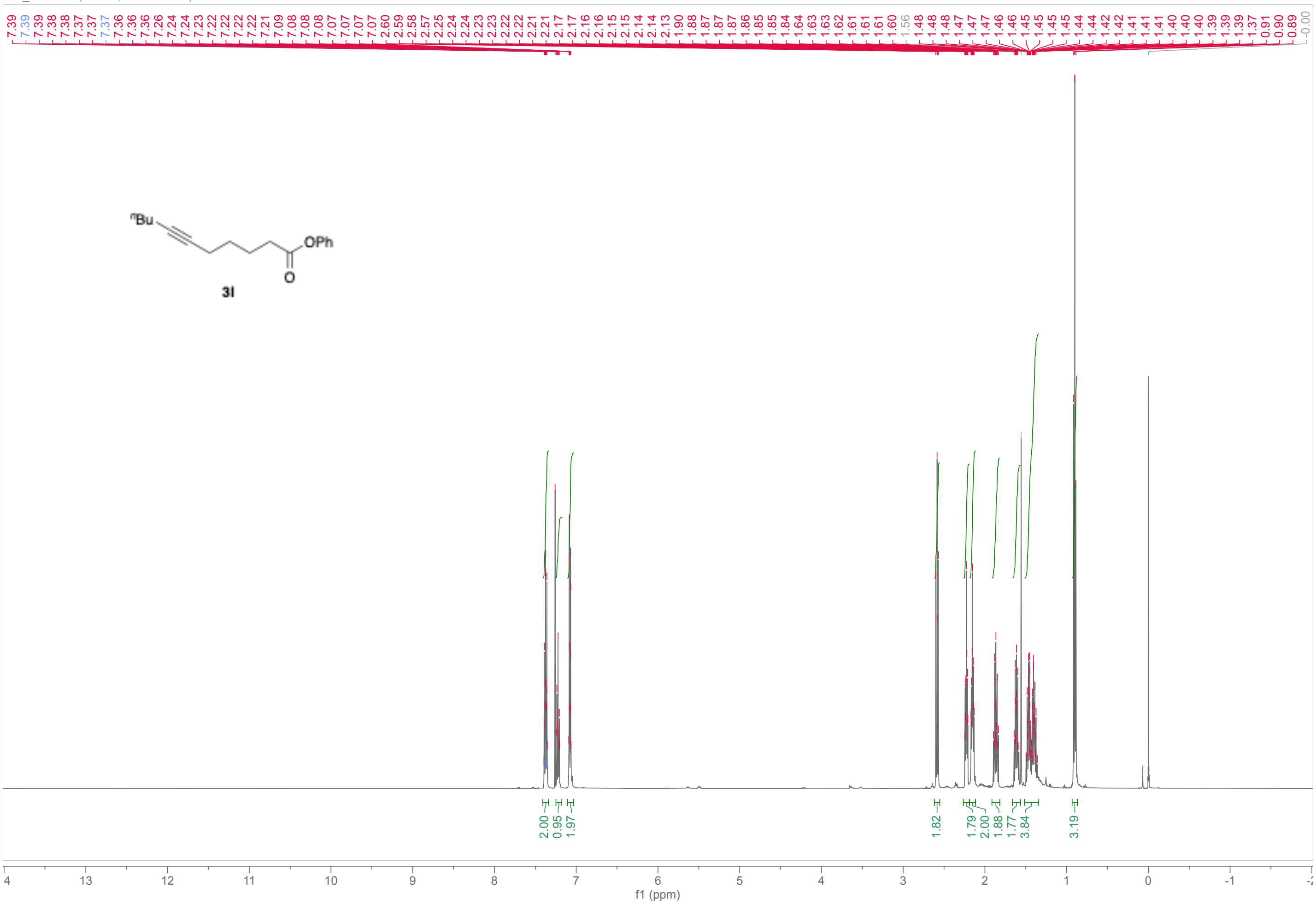

925_13C NMR (CDCl3, 125.72 MHz)

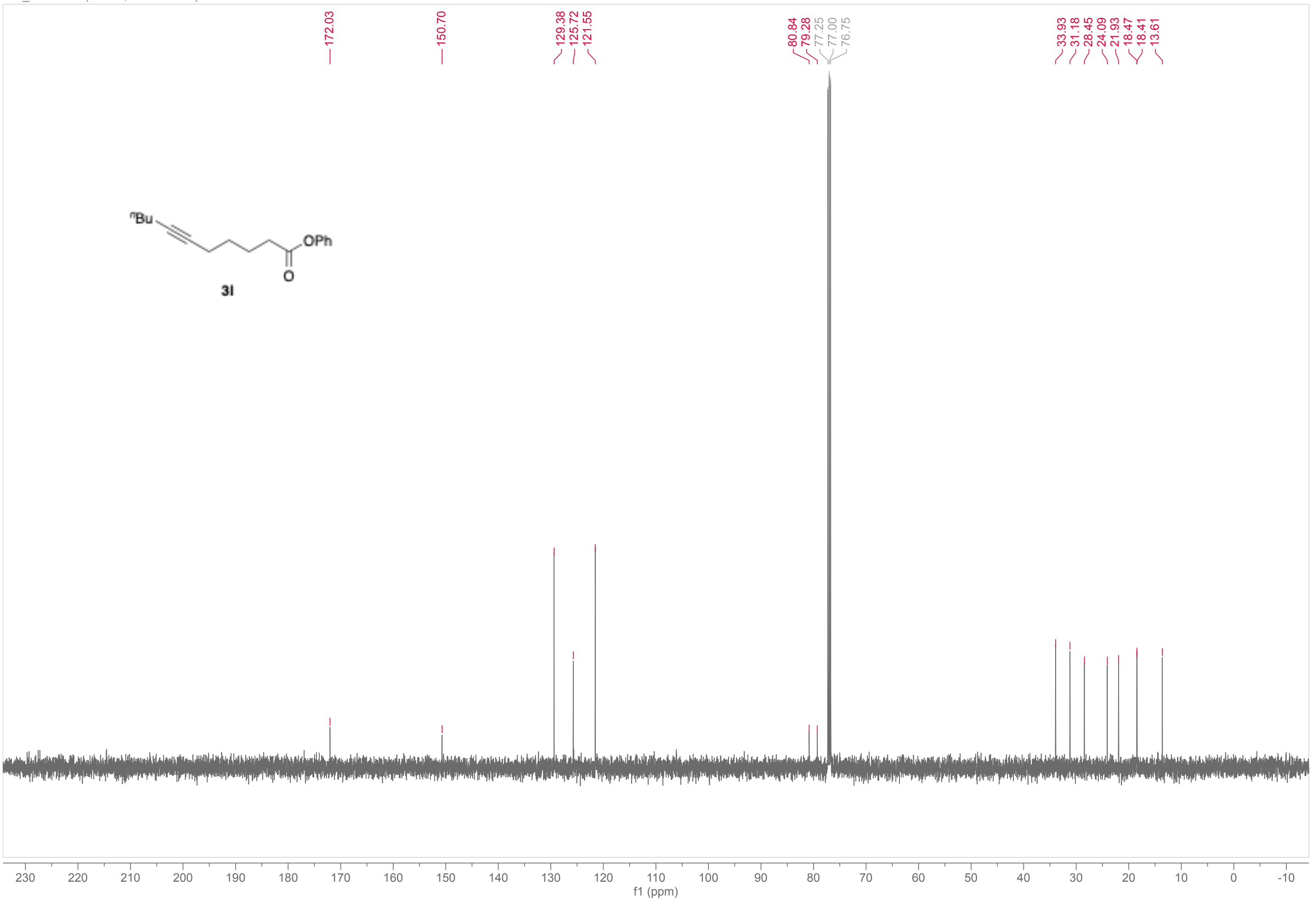

958_1H NMR (CDCl3, 499.94 MHz)

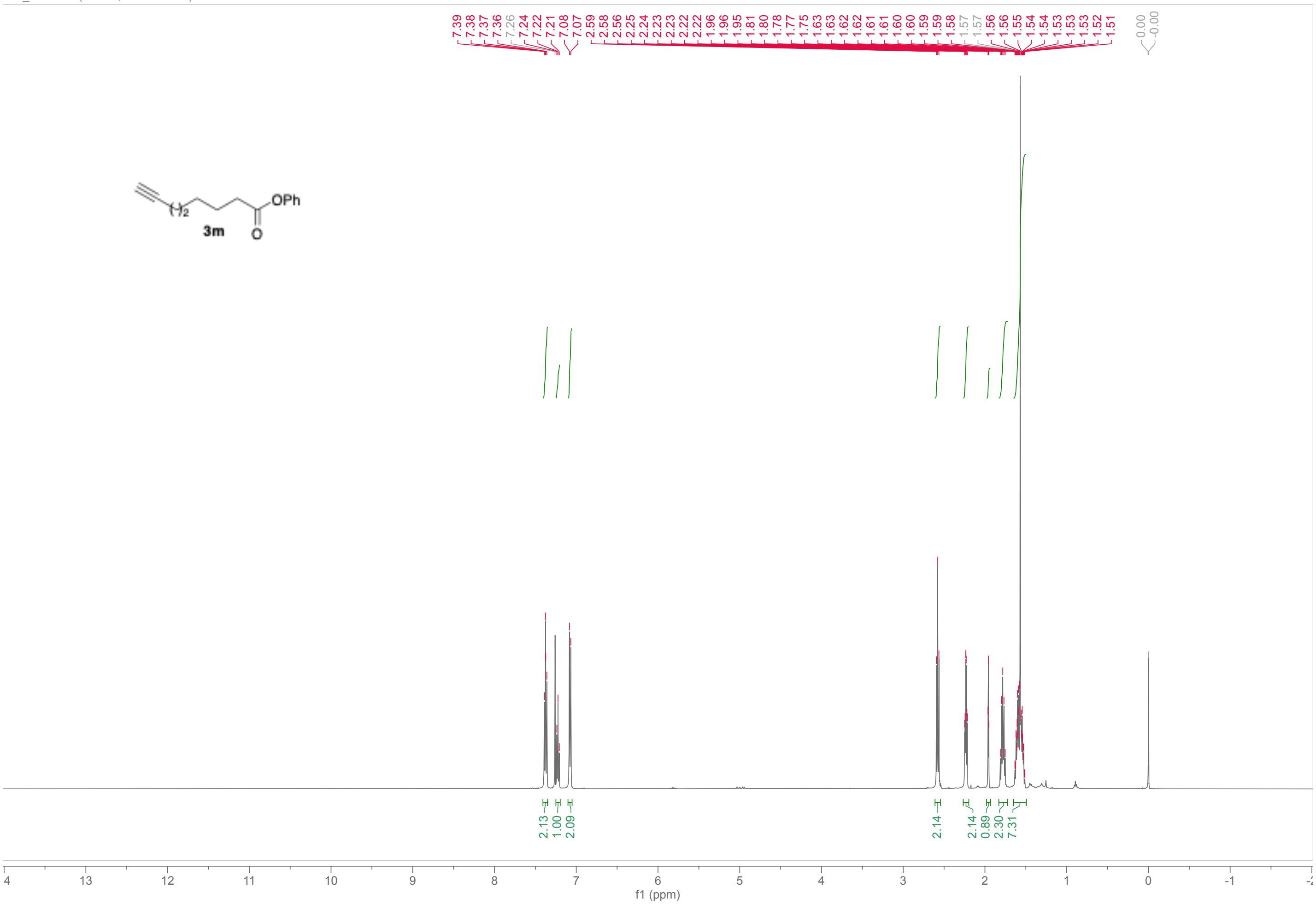

958_13C NMR (CDCl3, 125.72 MHz)

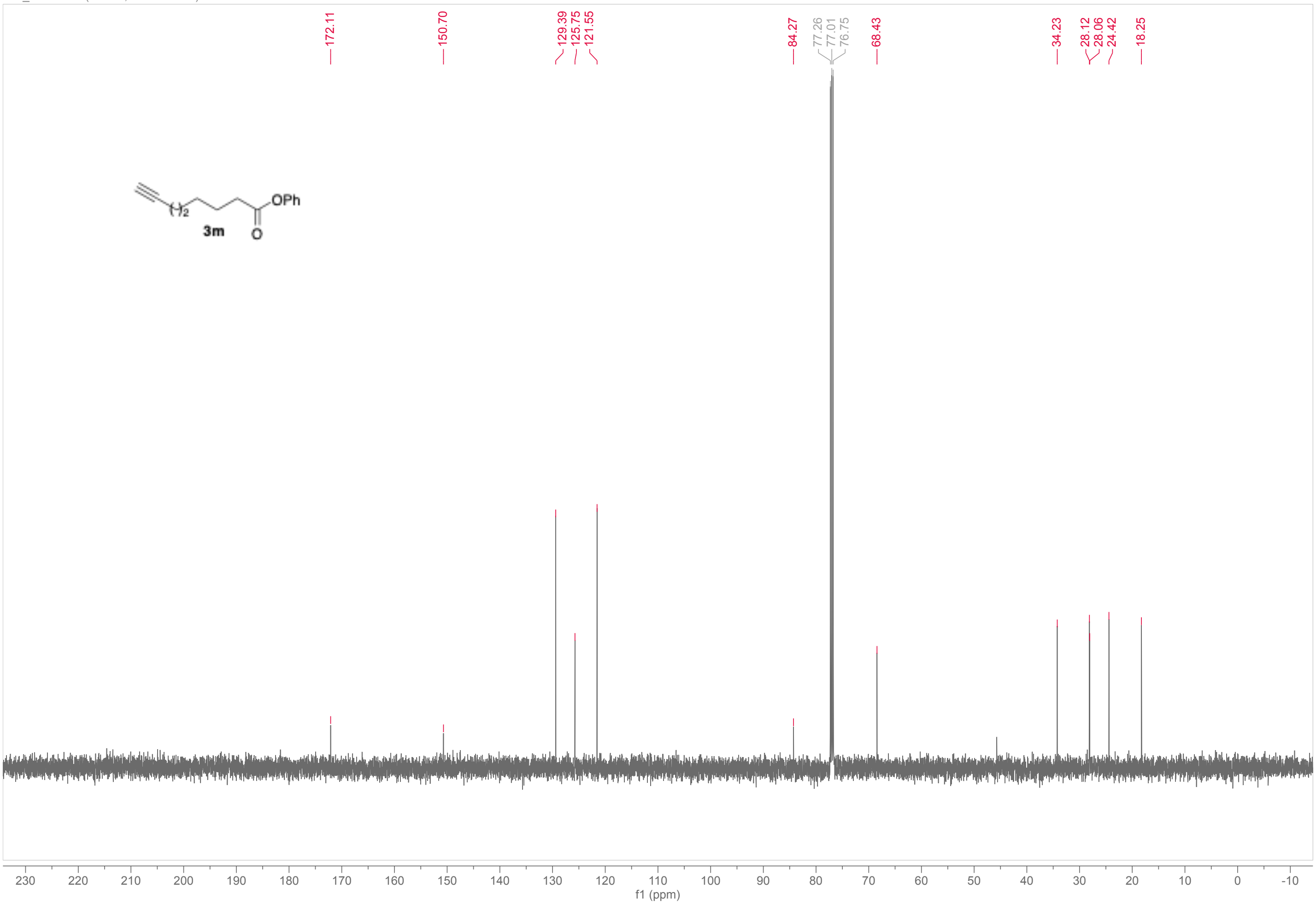

934_1H NMR (CDCl3, 499.94 MHz)

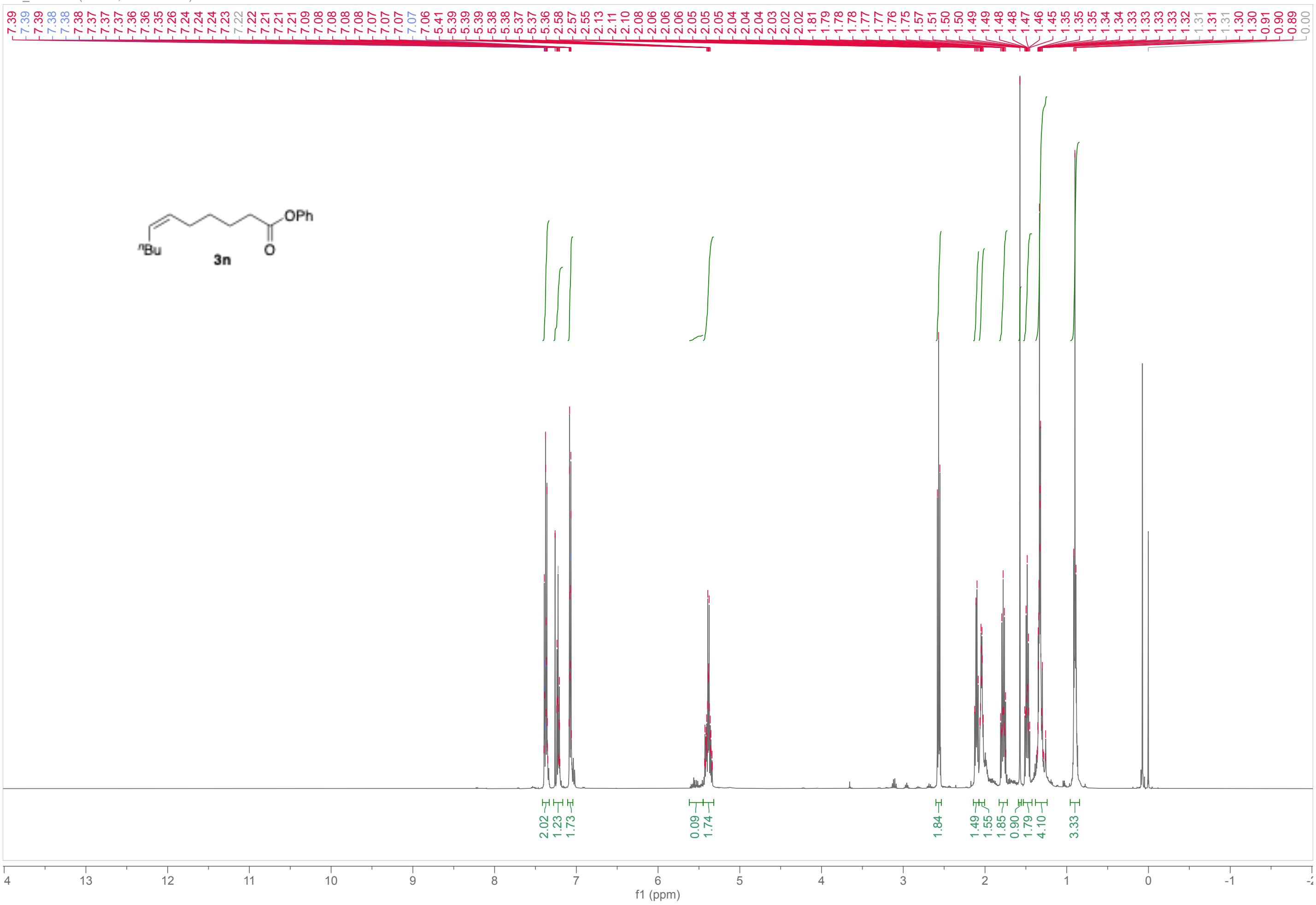

934_13C NMR (CDCl3, 125.72 MHz)

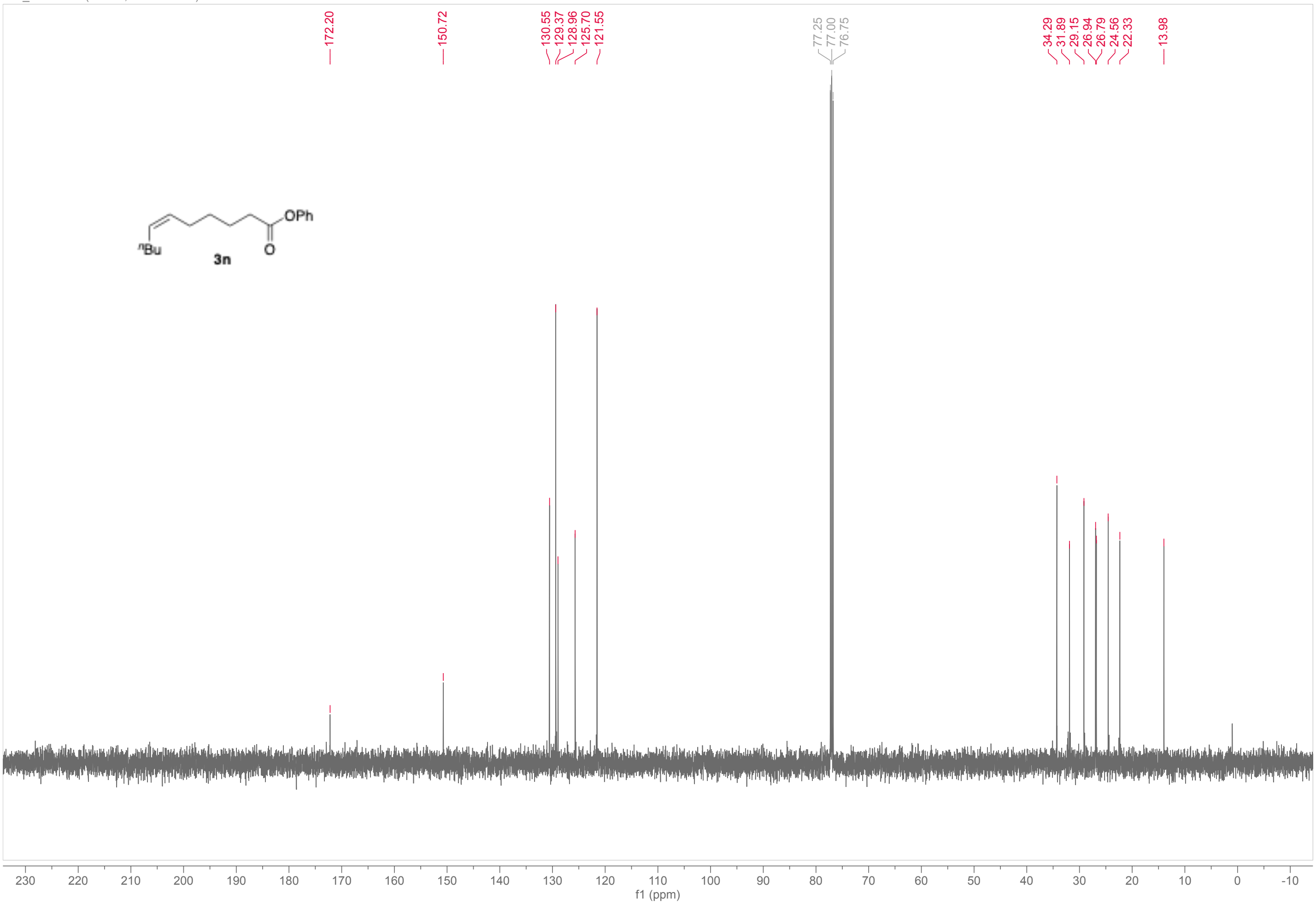

916_1H NMR (CDCl3, 499.94 MHz)

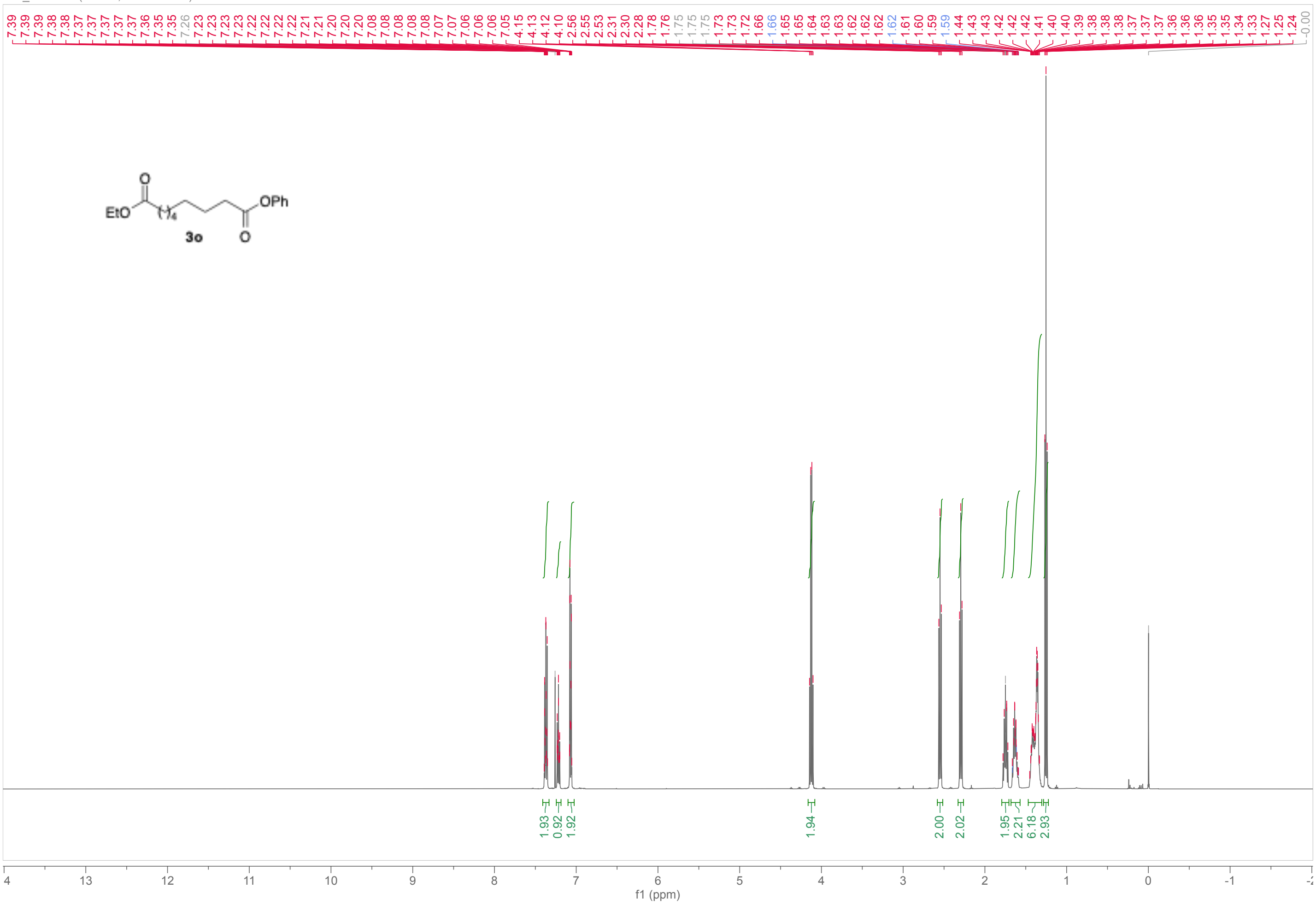

916_13C NMR (CDCl3, 125.72 MHz)

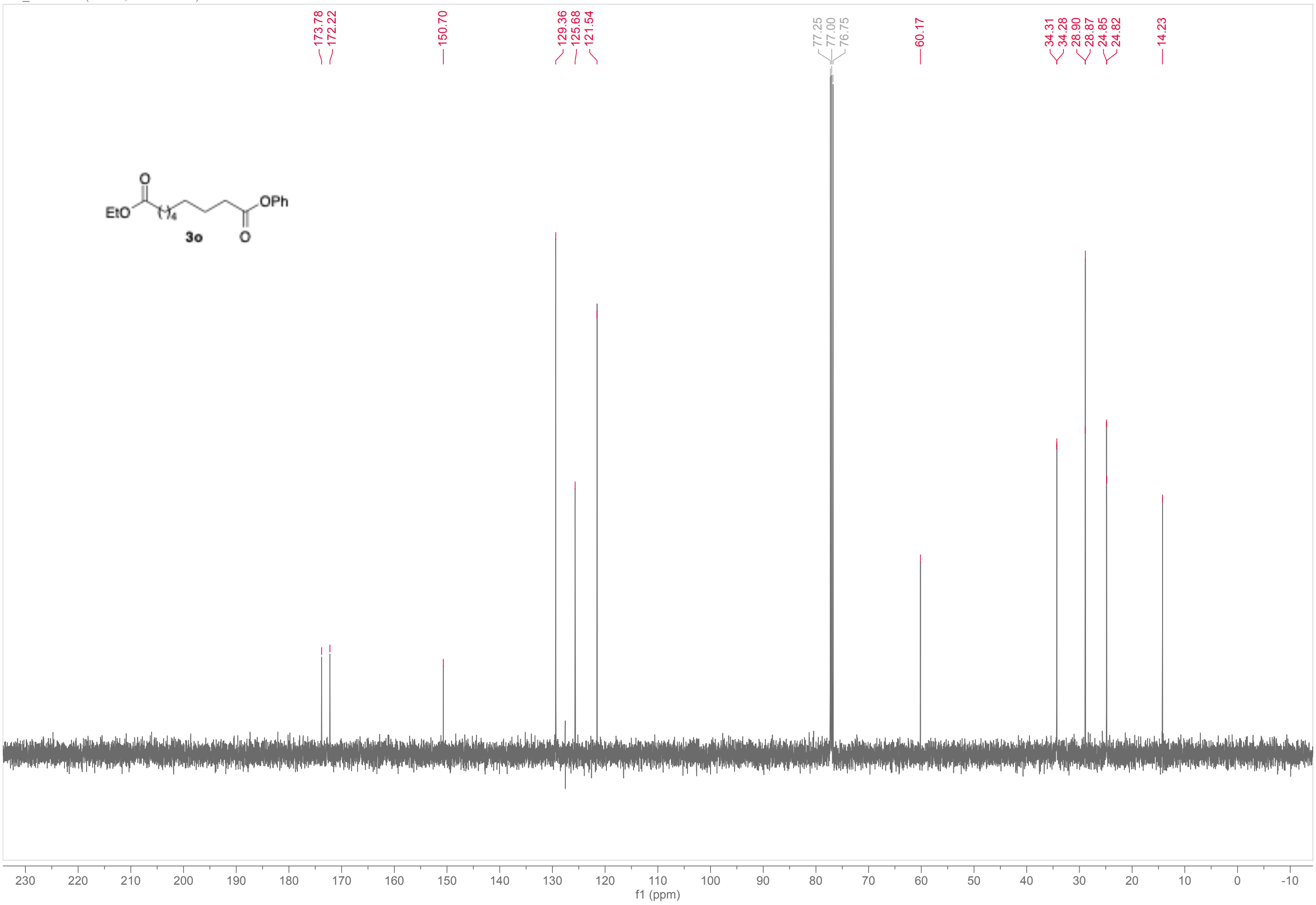

1008_1H NMR (CDCl3, 499.94 MHz)

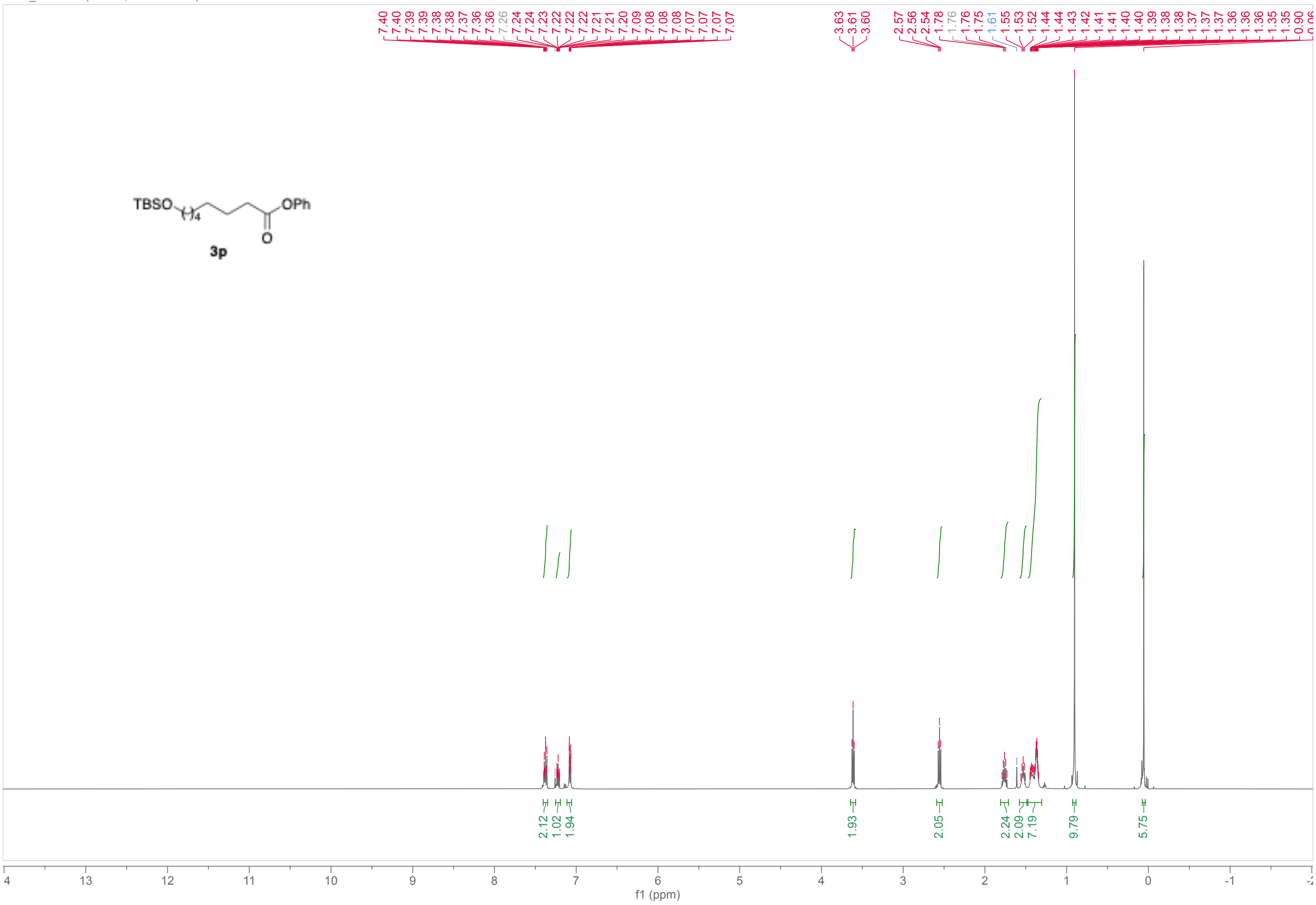

1008_13C NMR (CDCl3, 125.72 MHz)

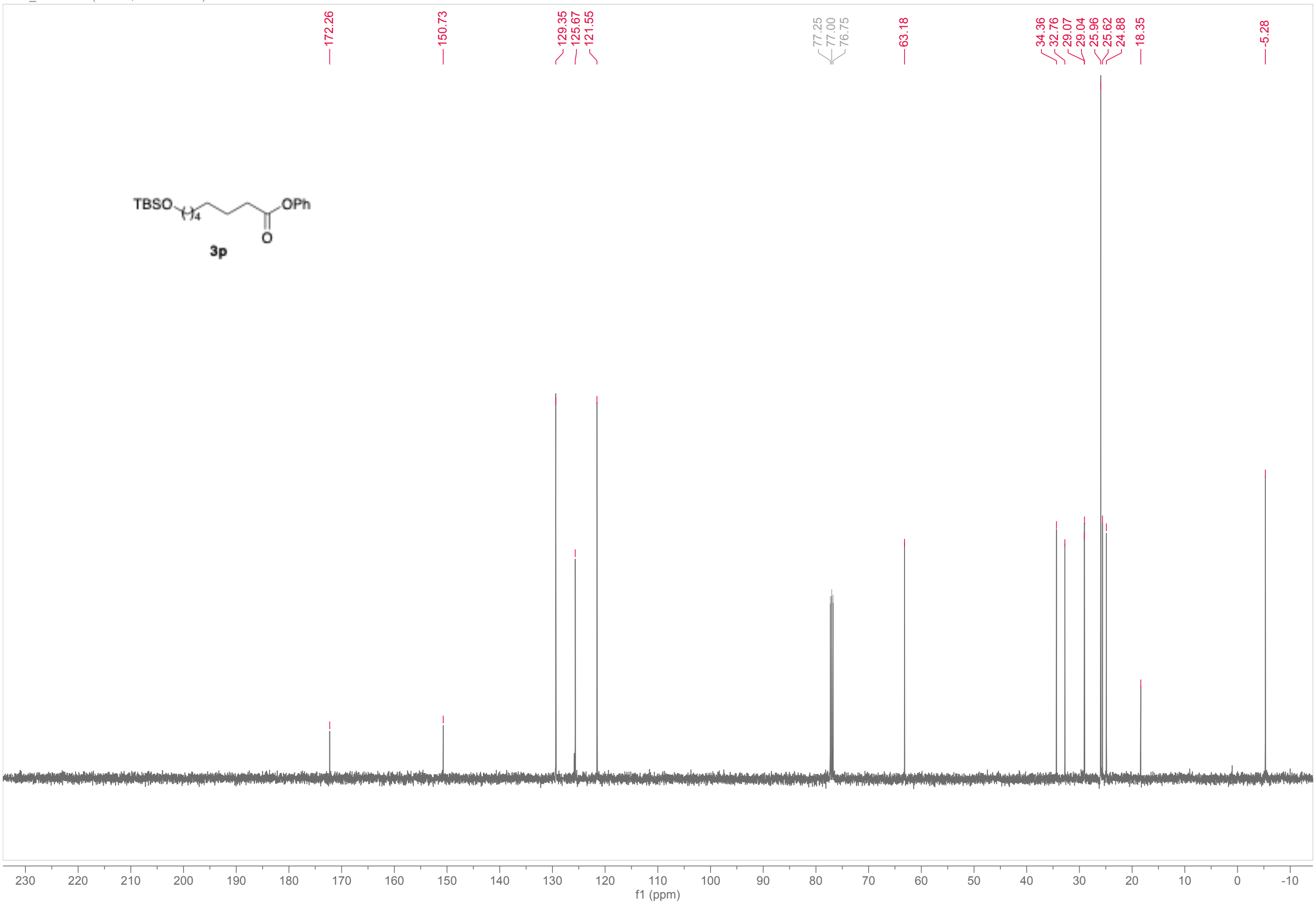

964_1H NMR (CDCl3, 499.94 MHz at 25.0 °C)

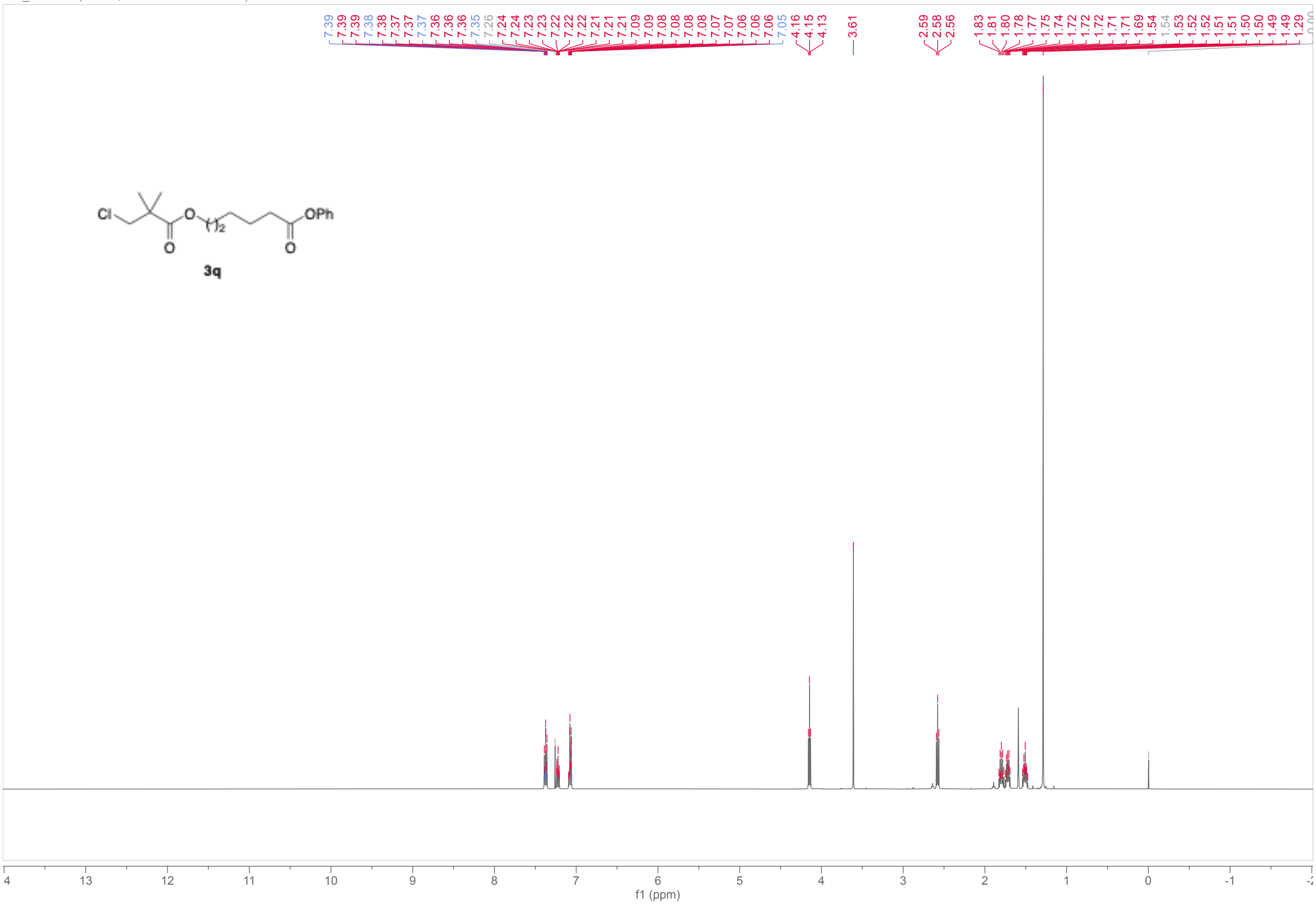

964_13C NMR (CDCl3, 125.72 MHz at 25.0 °C)

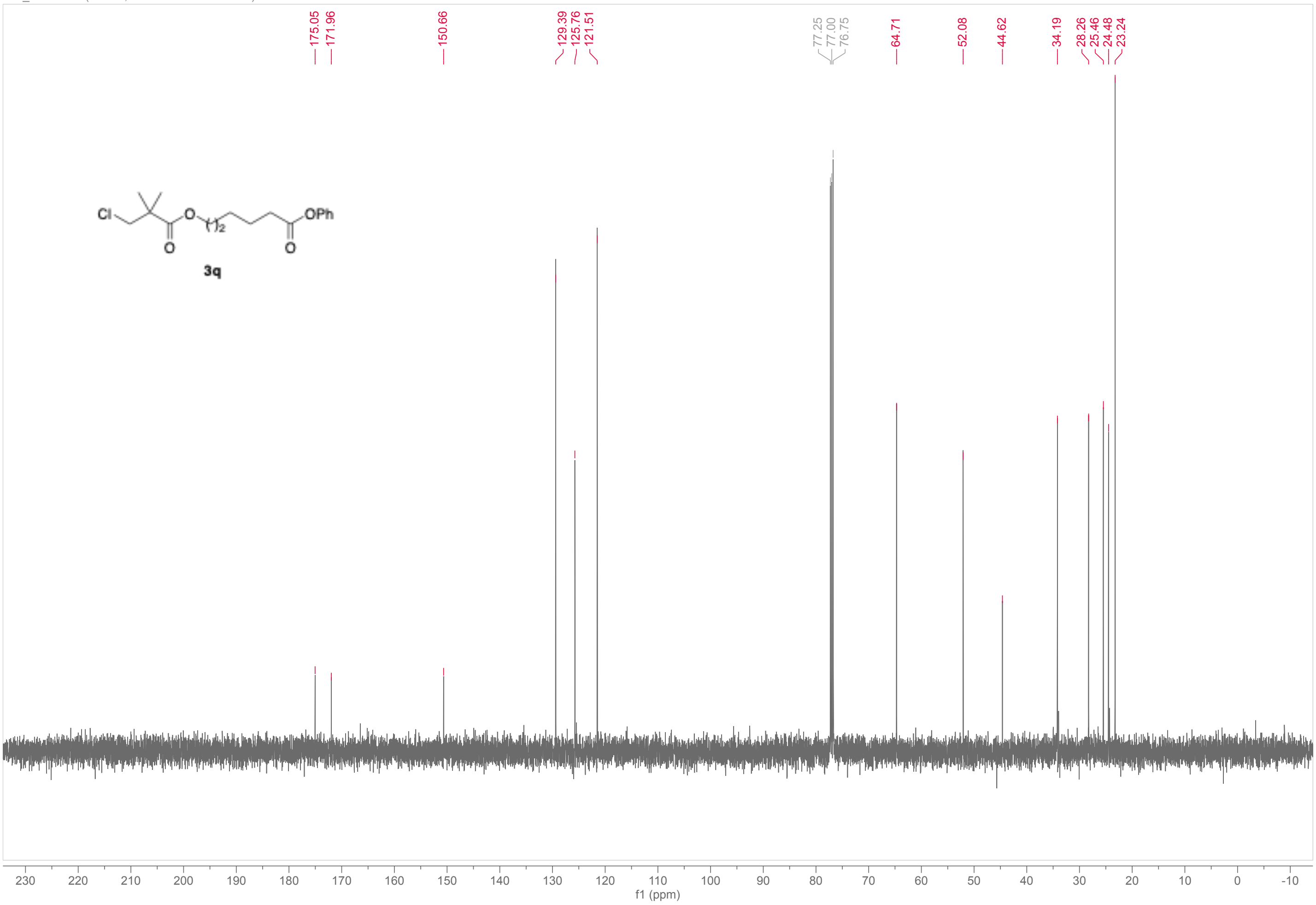

991_1H NMR (CDCl3, 499.94 MHz at 25.0 °C)

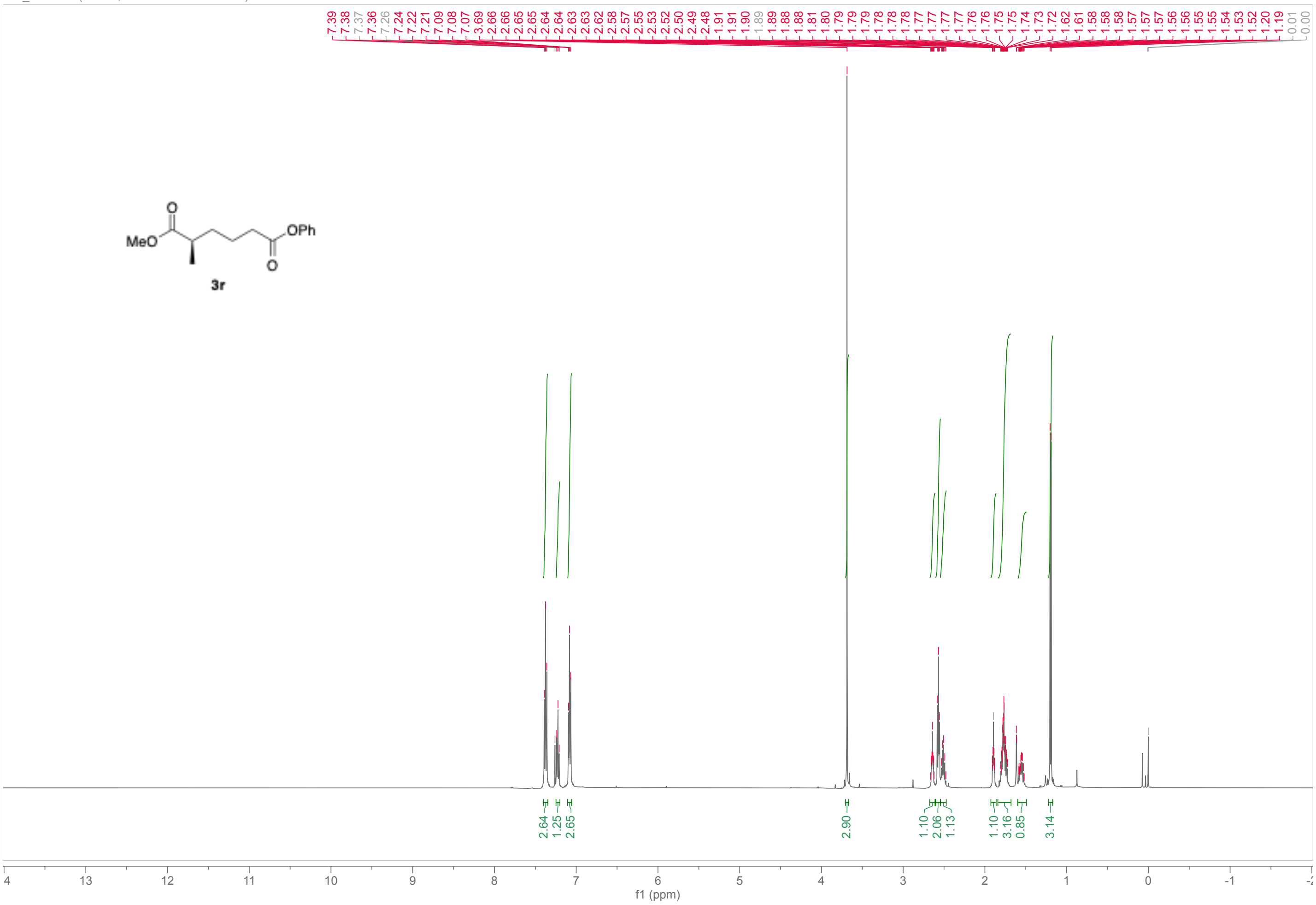

991_13C NMR (CDCl3, 125.72 MHz at 25.0 °C)

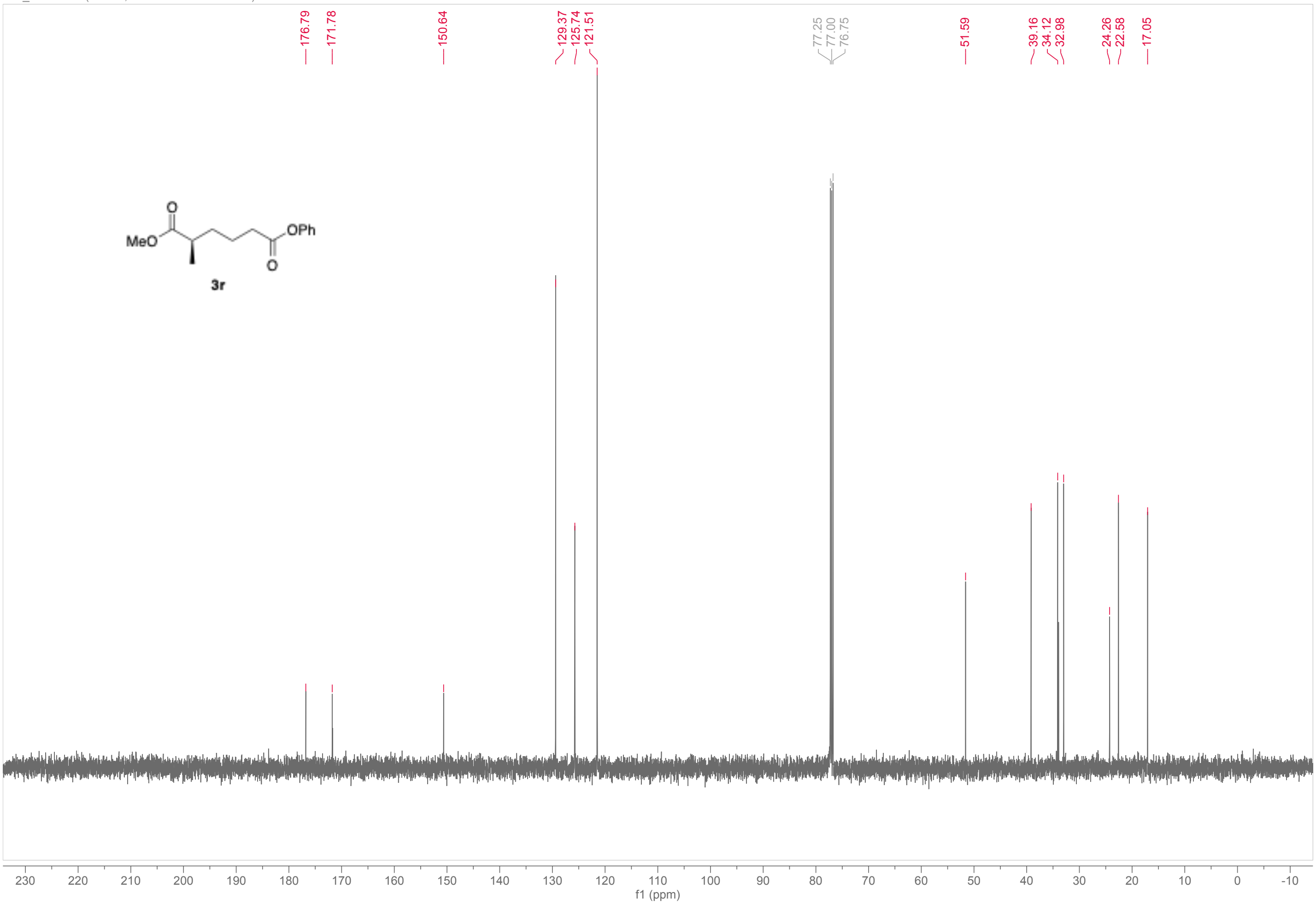

866_1H NMR (CDCl3, 499.94 MHz)

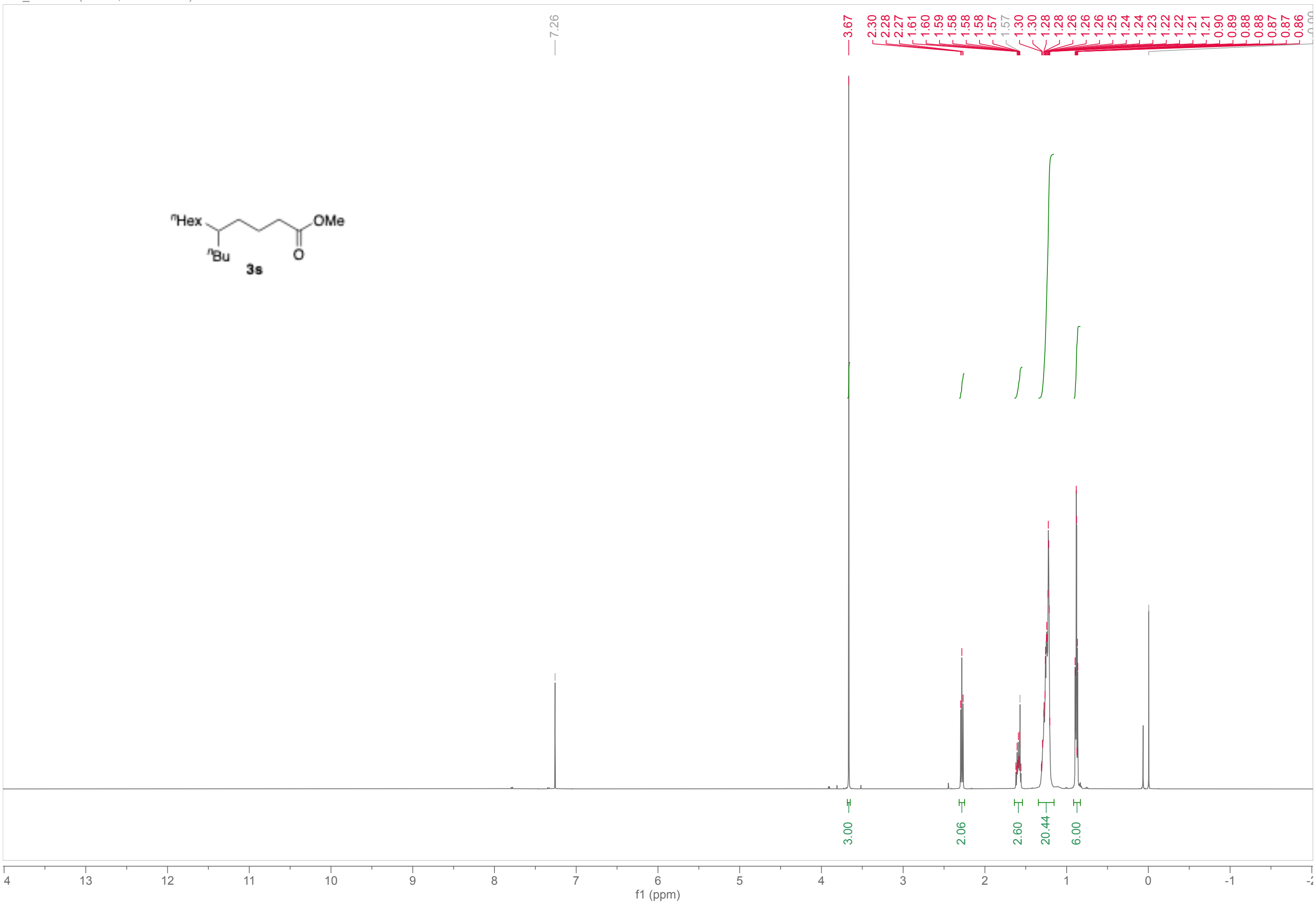

866_13C NMR (CDCl3, 125.72 MHz)

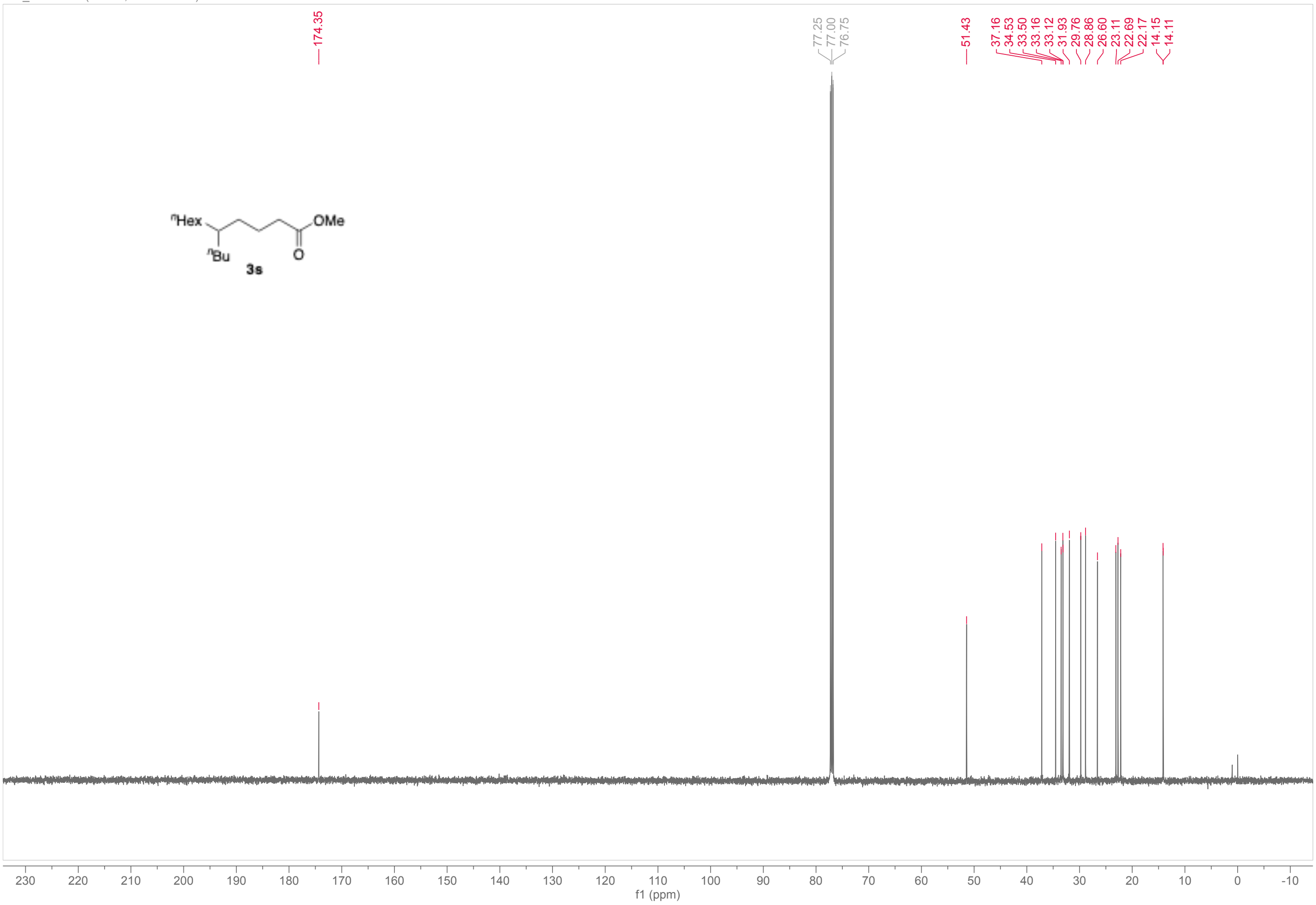

909_1H NMR (CDCl3, 399.89 MHz)

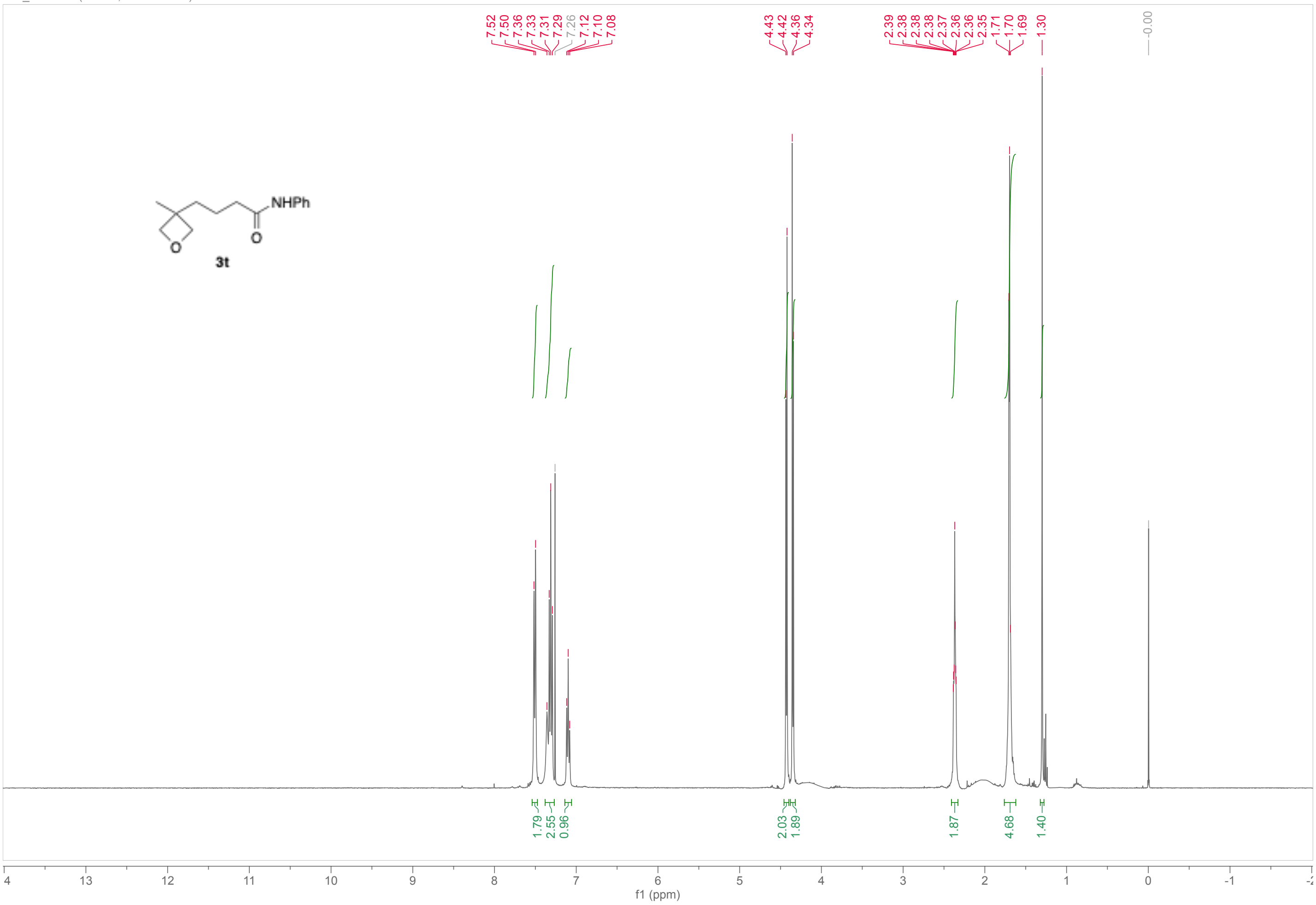

909_13C NMR (CDCl3, 125.72 MHz)

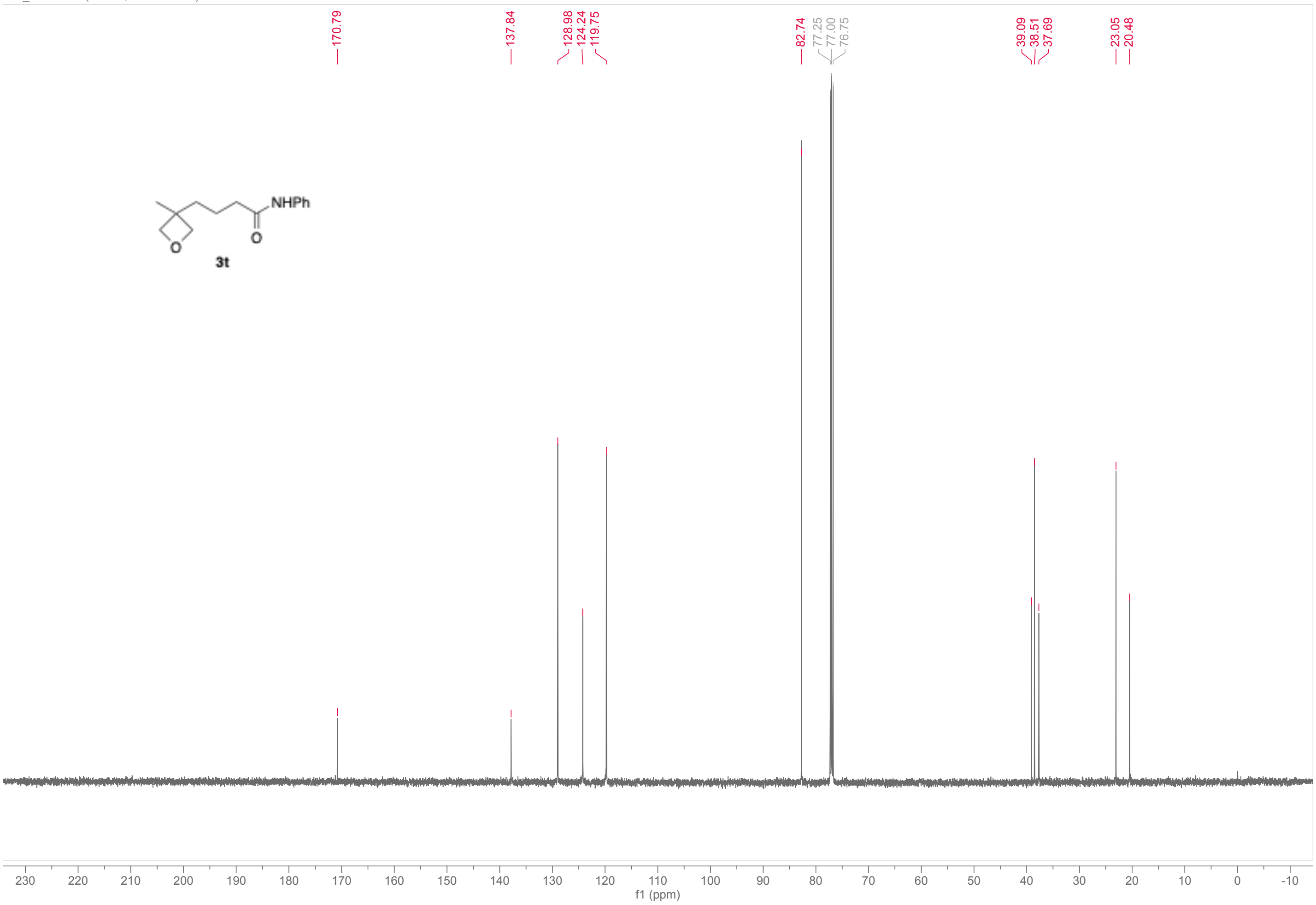

908_1H NMR (CDCl3, 499.94 MHz)

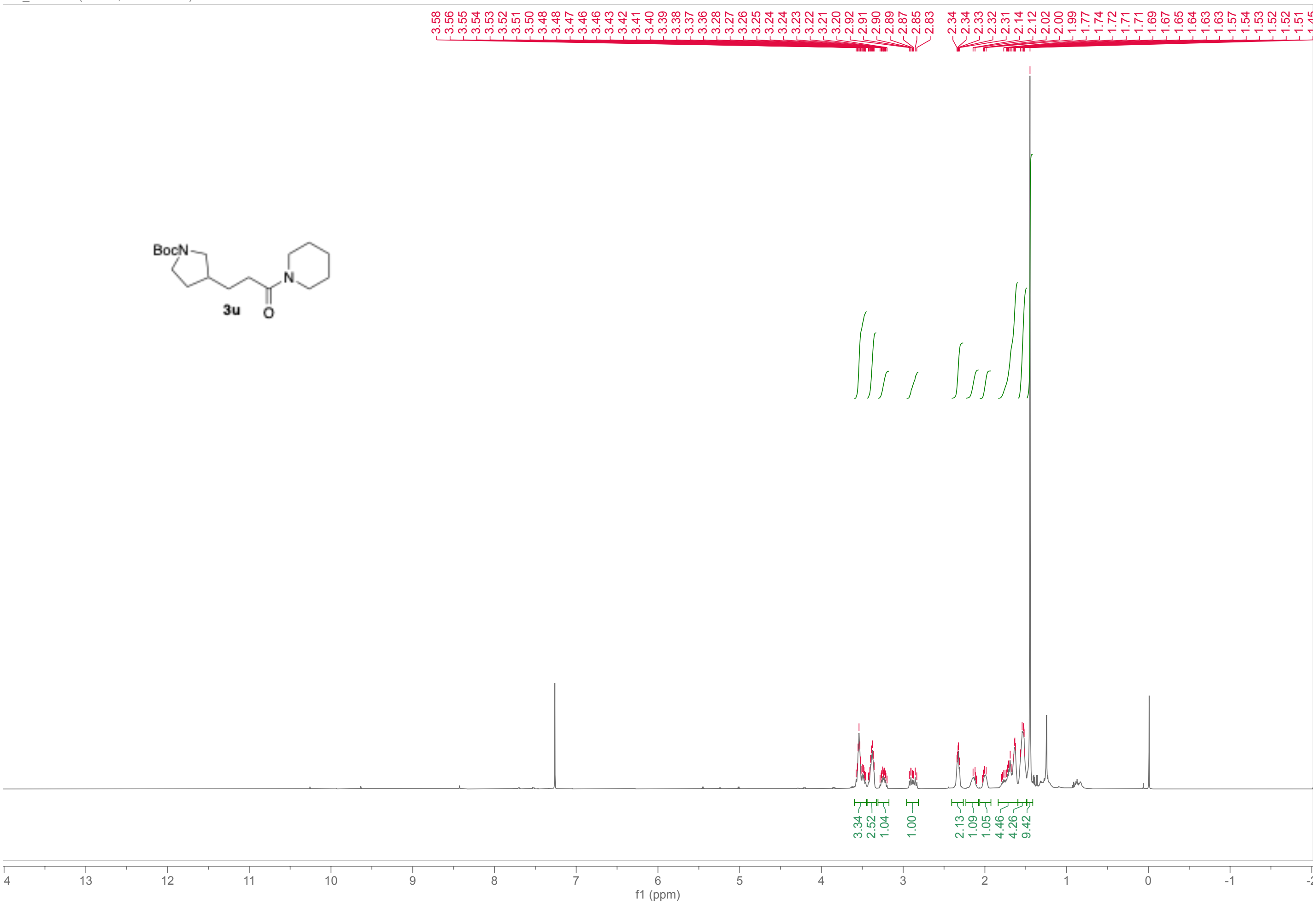

908_13C NMR (CDCl3, 125.72 MHz)

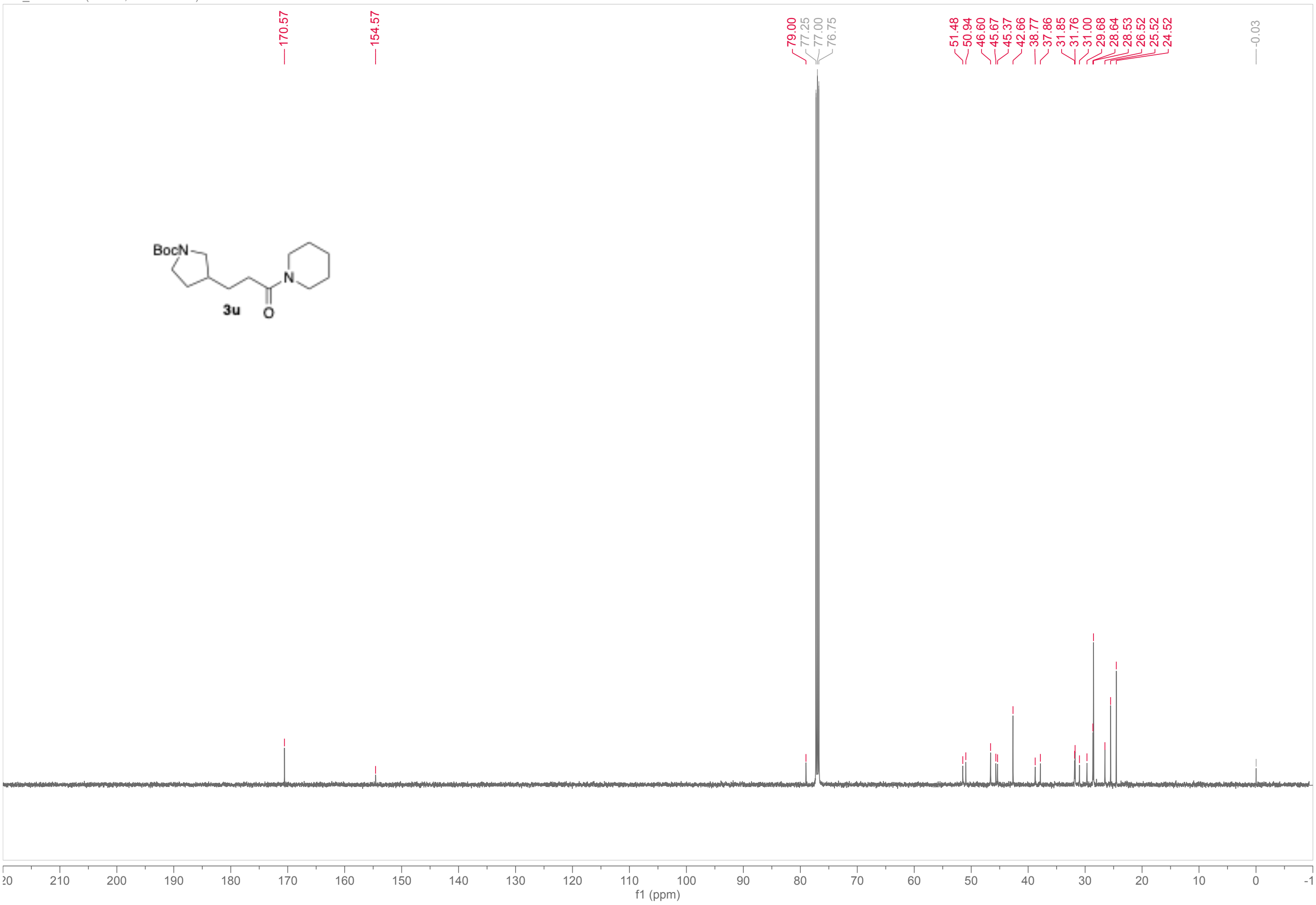

994_1H NMR (CDCl3, 499.94 MHz at 60.0 °C)

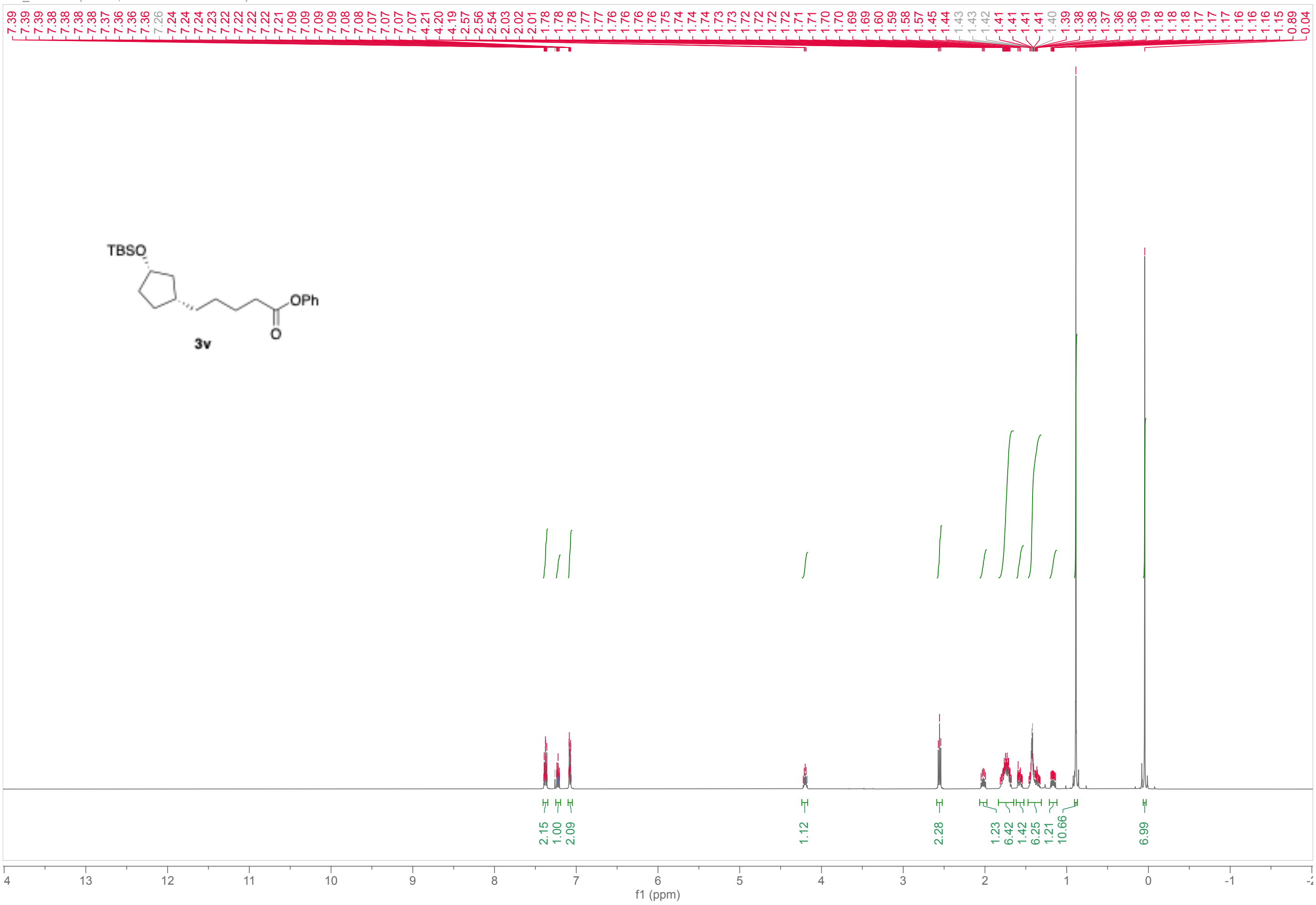

994_13C NMR (CDCl3, 125.72 MHz at 60.0 °C)

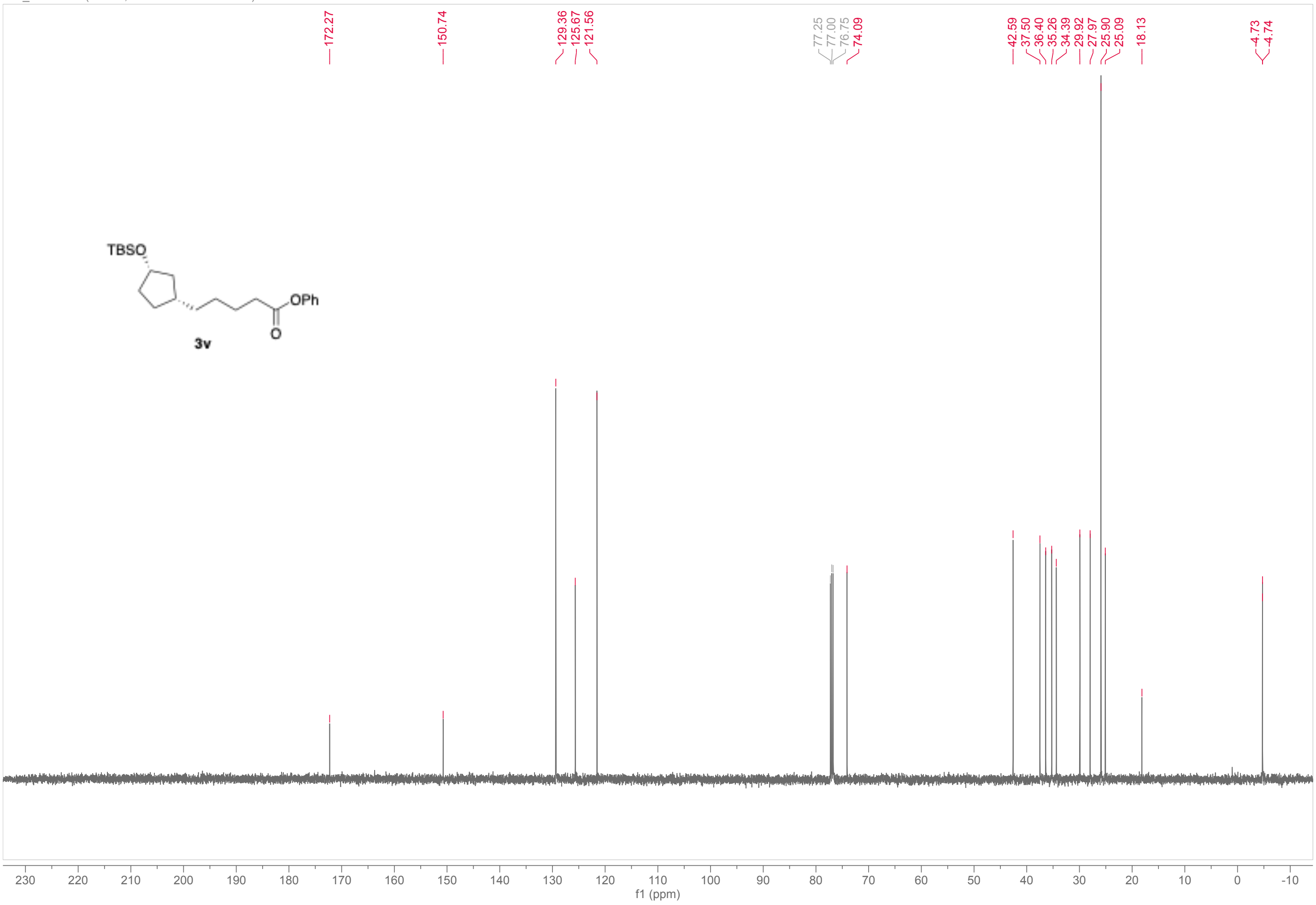

995_1H NMR (CDCl3, 499.94 MHz at 60.0 ºC)

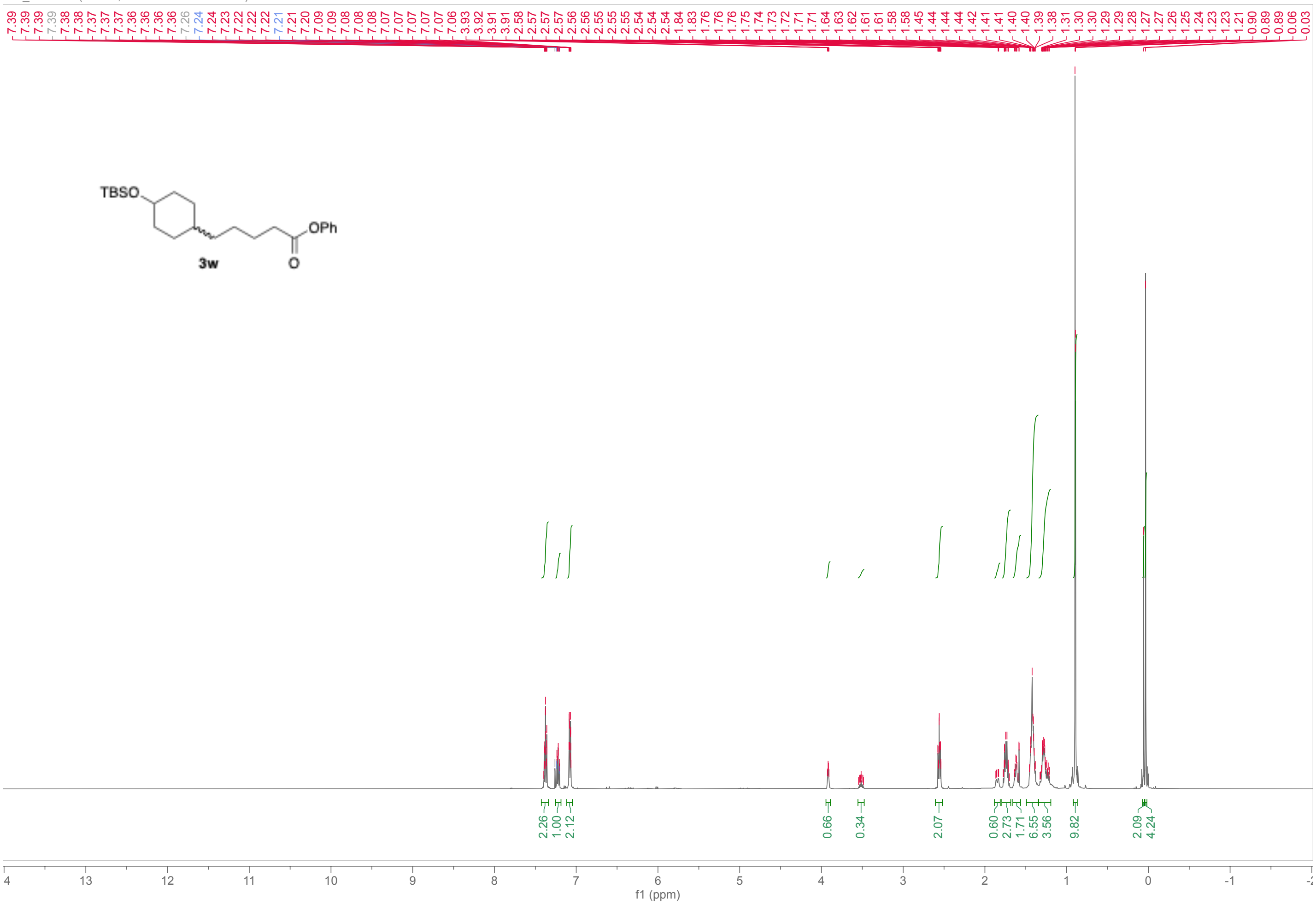

995_13C NMR (CDCl3, 125.72 MHz at 60.0 ºC)

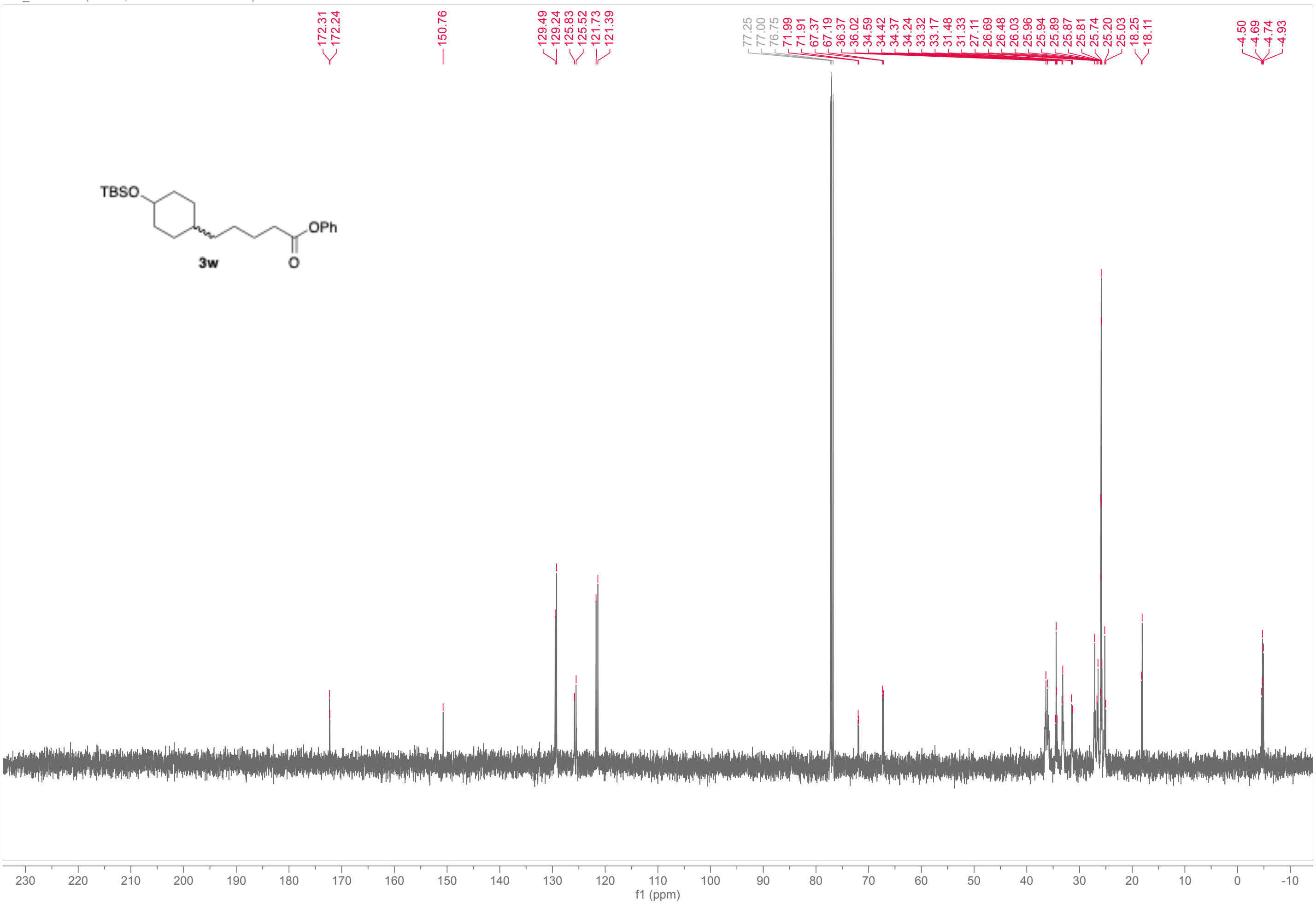

959_1H NMR (CDCl3, 499.94 MHz)

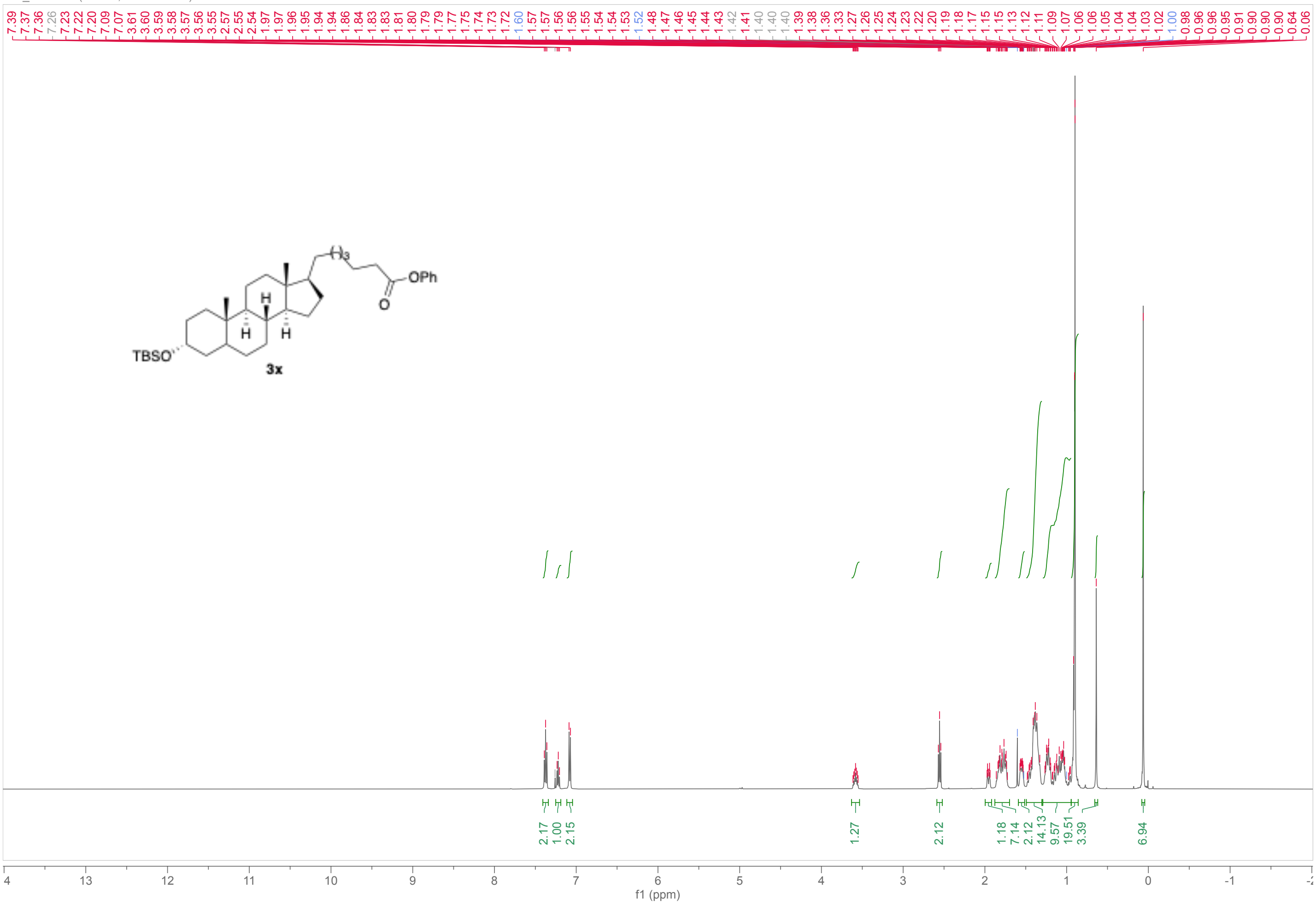

959_13C NMR (CDCl3, 125.72 MHz)

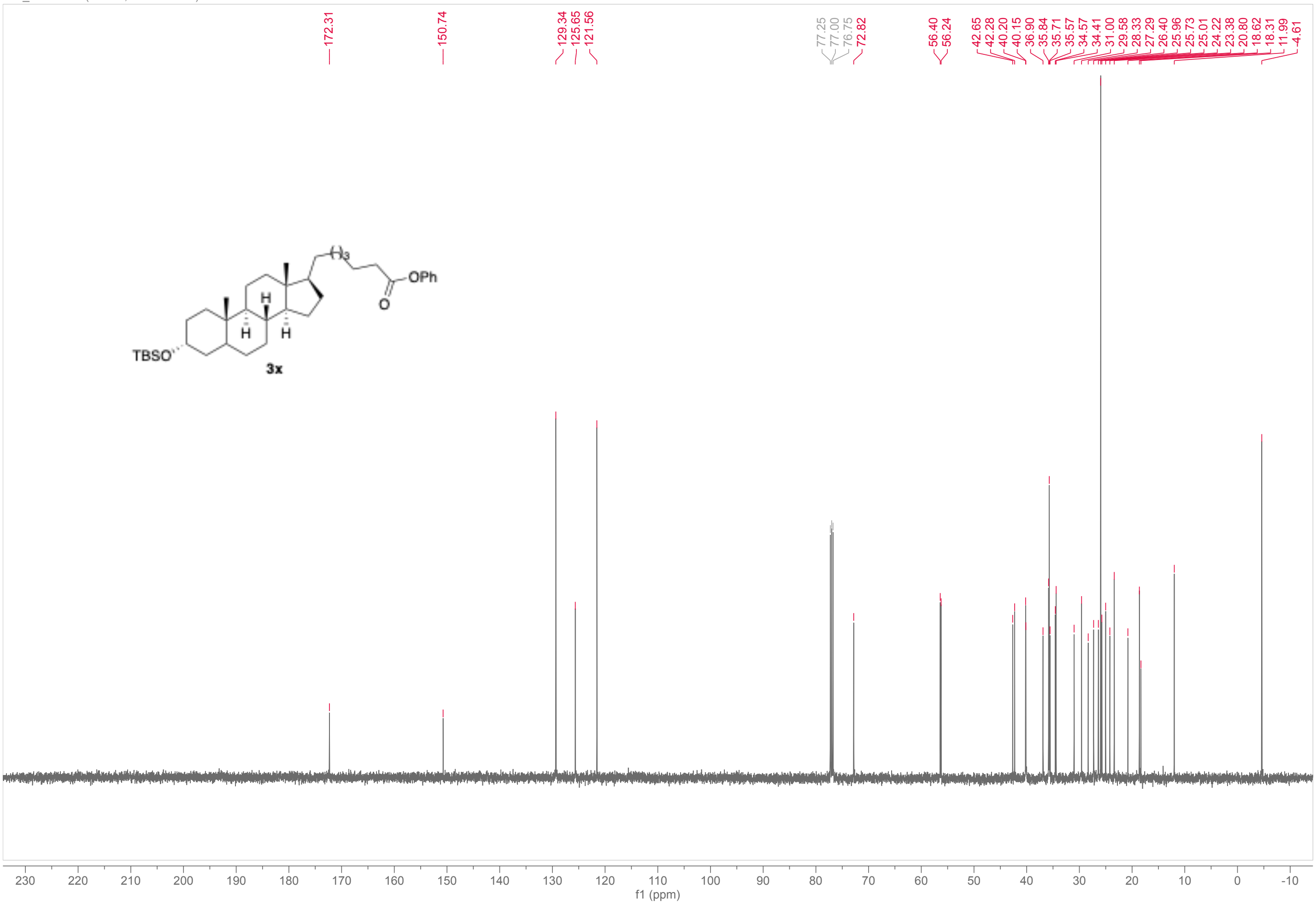

932_1H NMR (CDCl3, 499.94 MHz)

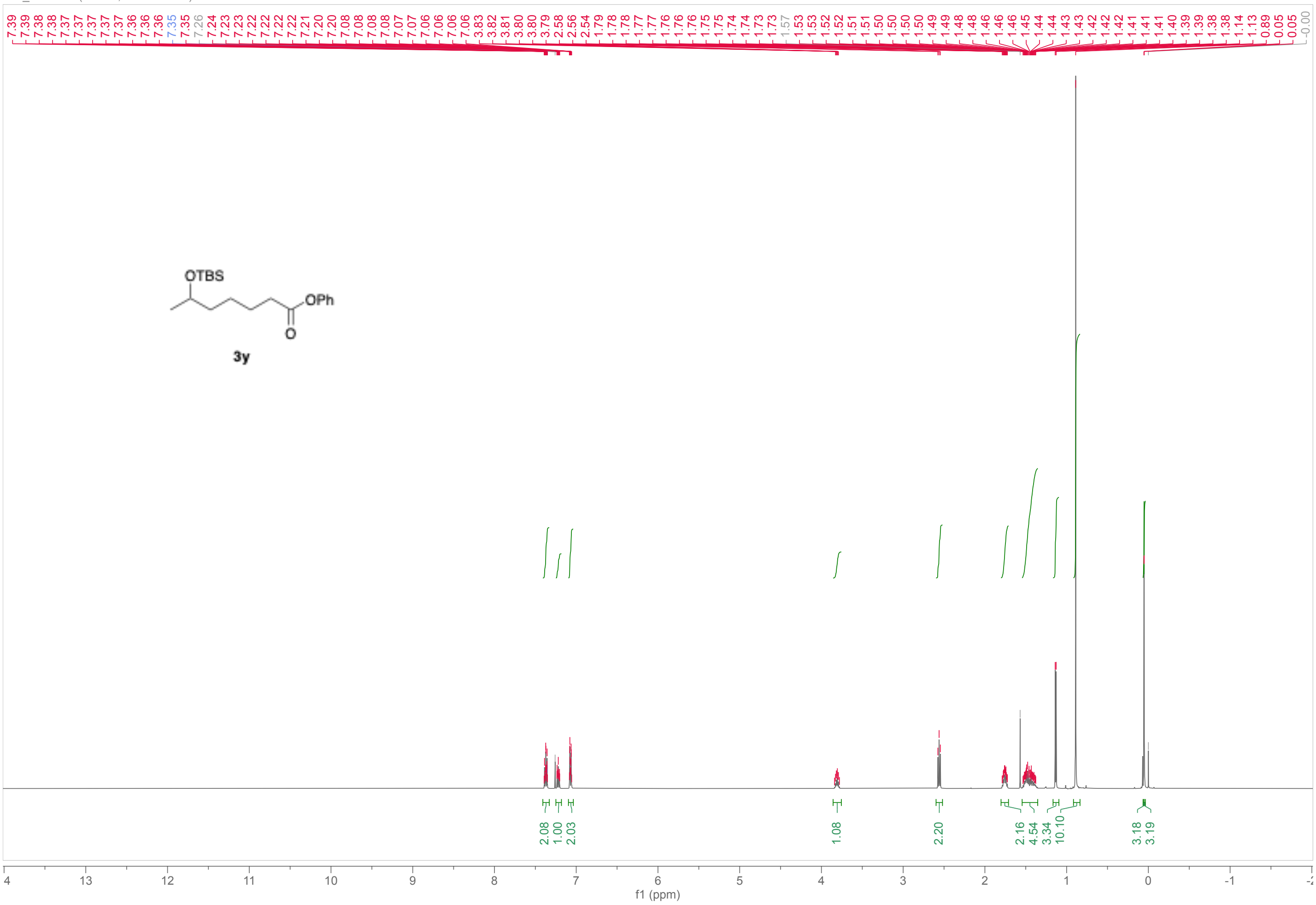

932_13C NMR (CDCl3, 125.72 MHz)

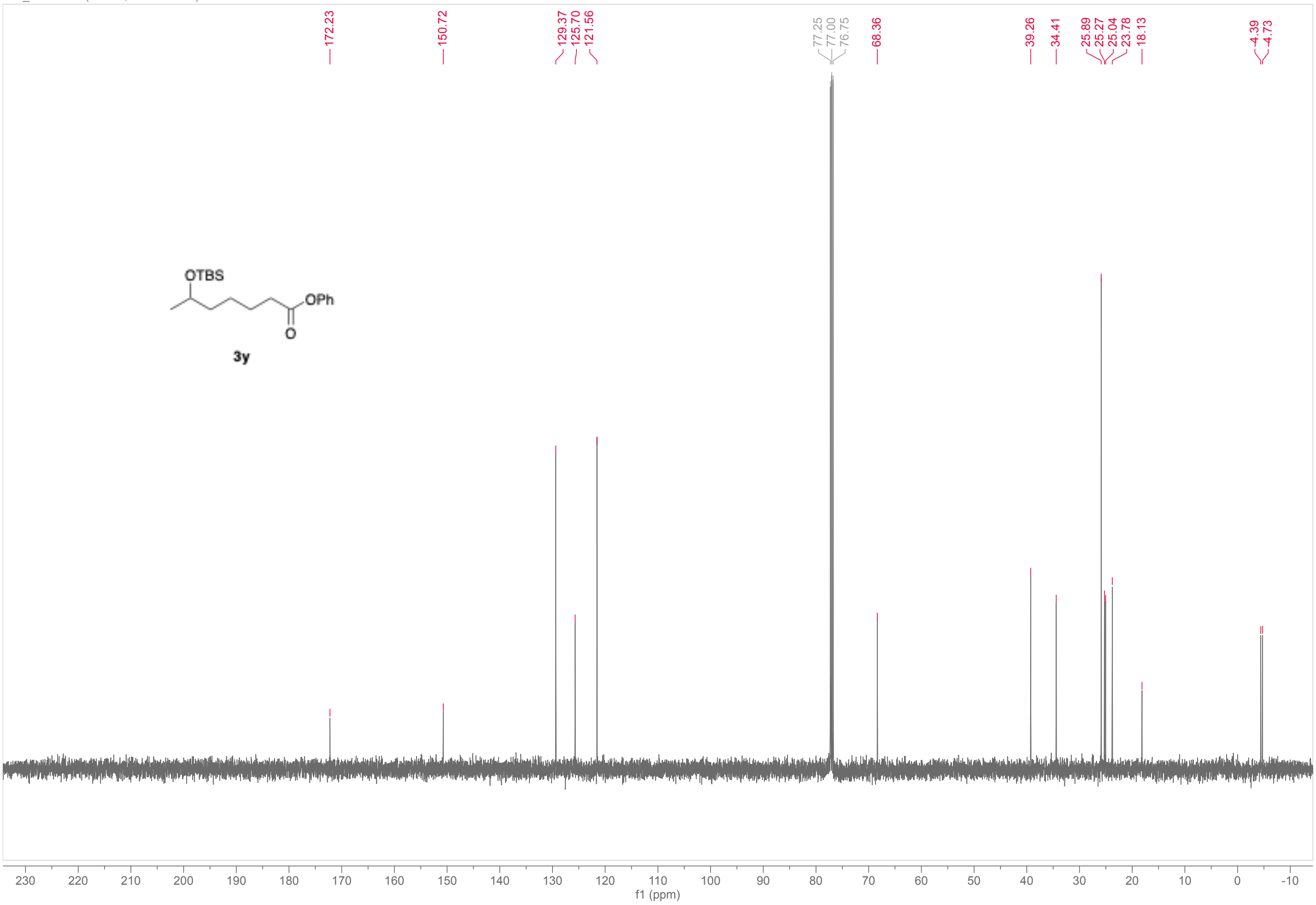

992_1H NMR (CDCl3, 499.94 MHz at 25.0 °C)

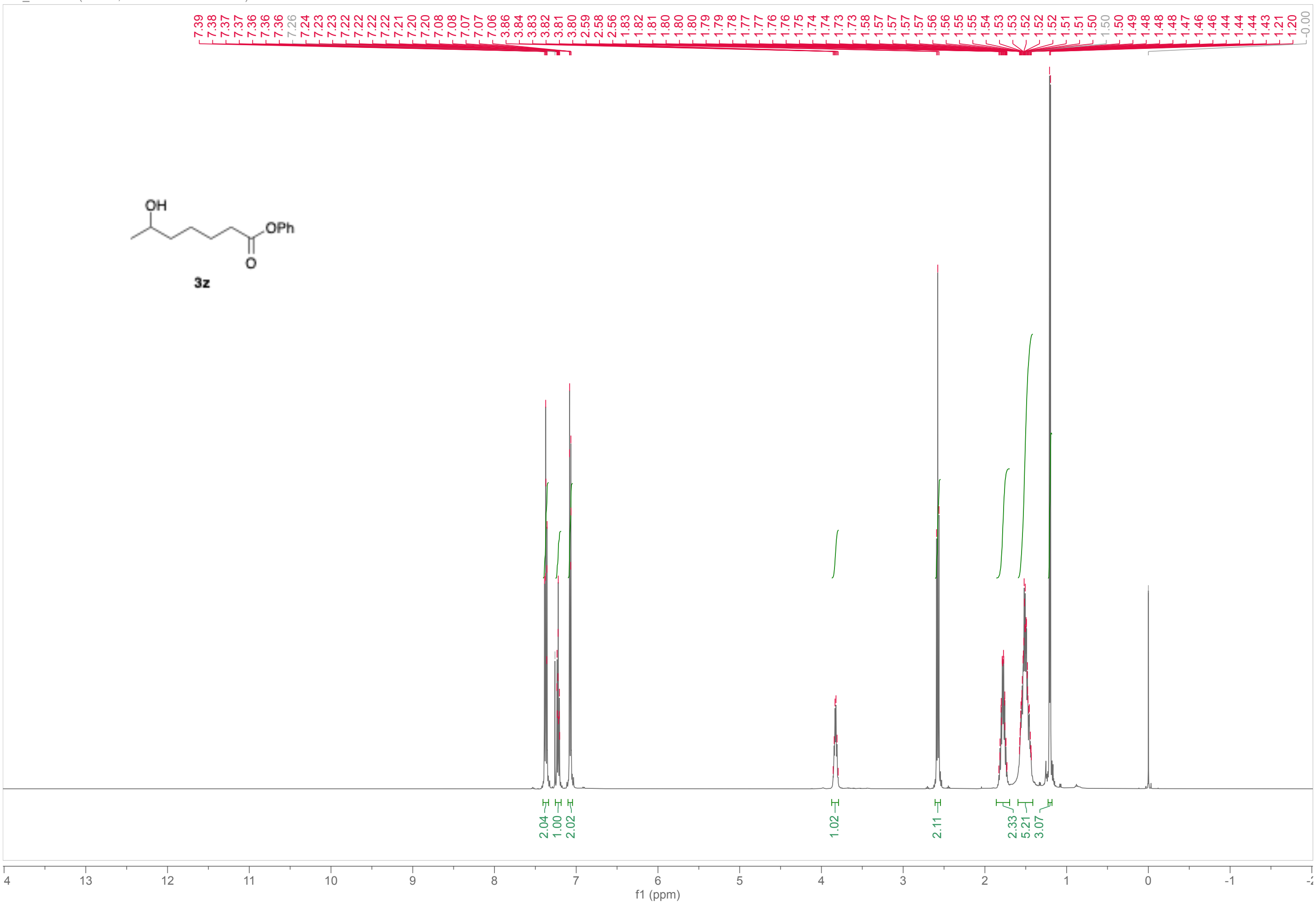

992_13C NMR (CDCl3, 125.72 MHz at 25.0 °C)

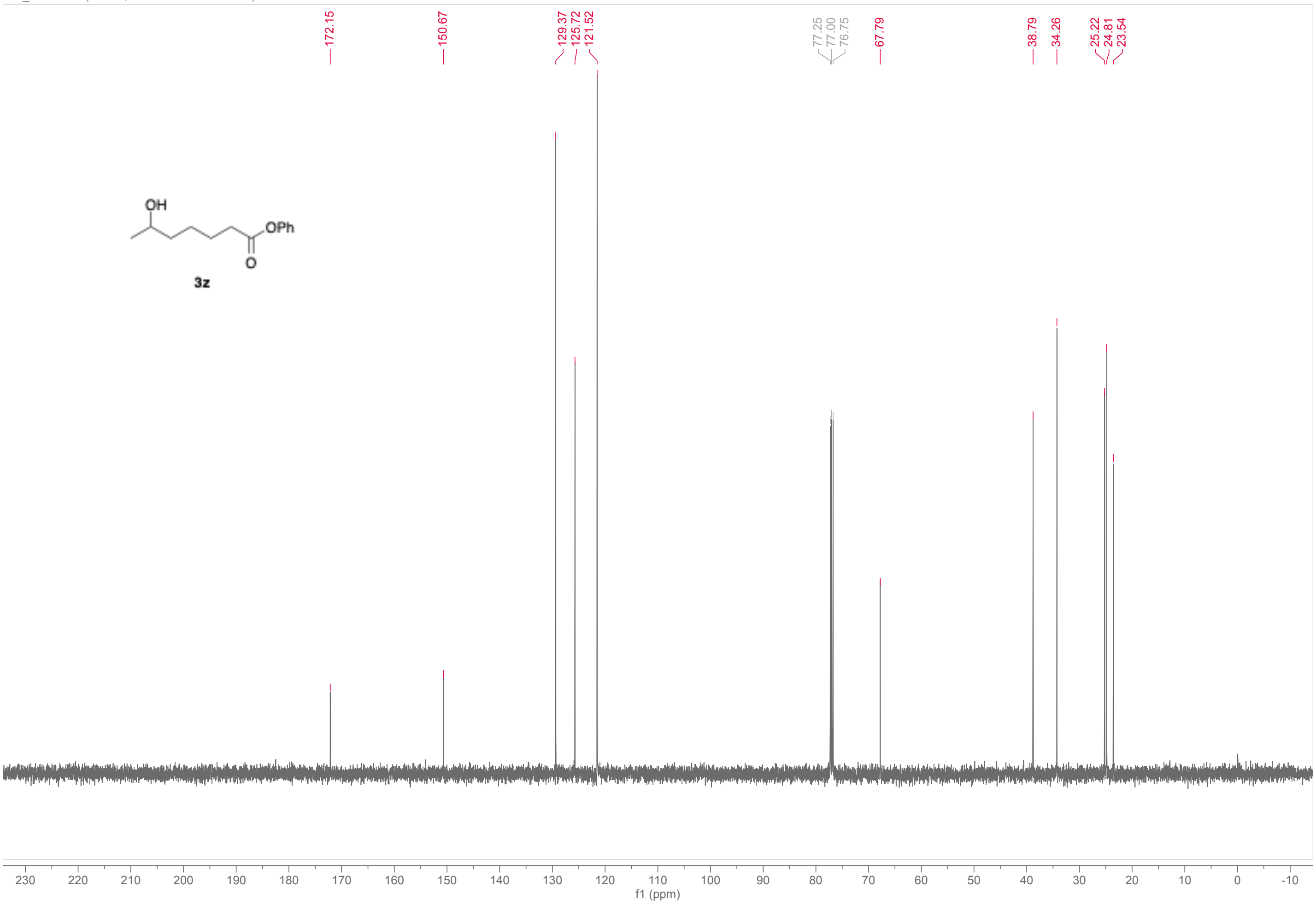

871_1H NMR (CDCl3, 499.94 MHz)

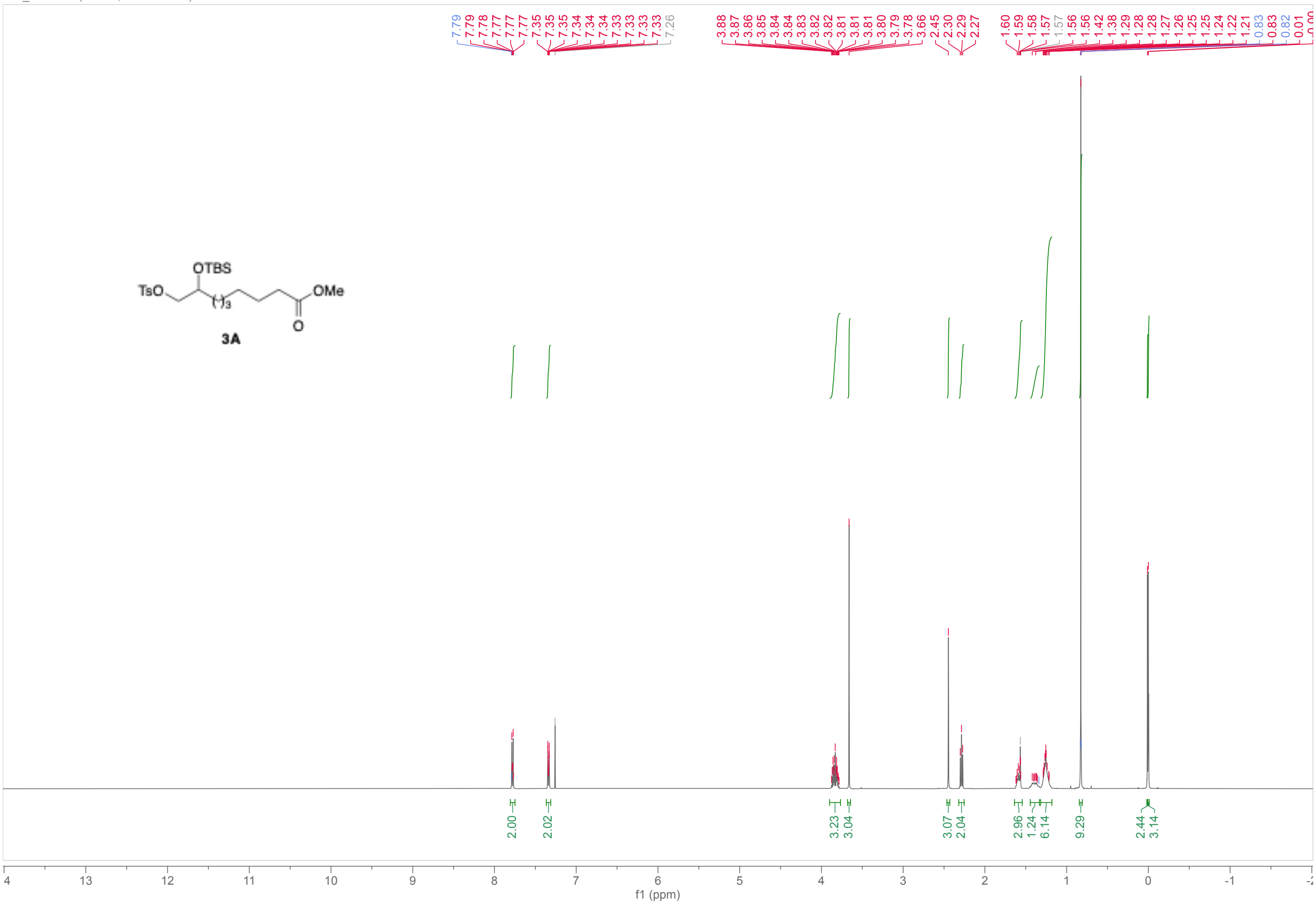

871_13C NMR (CDCl3, 125.72 MHz)

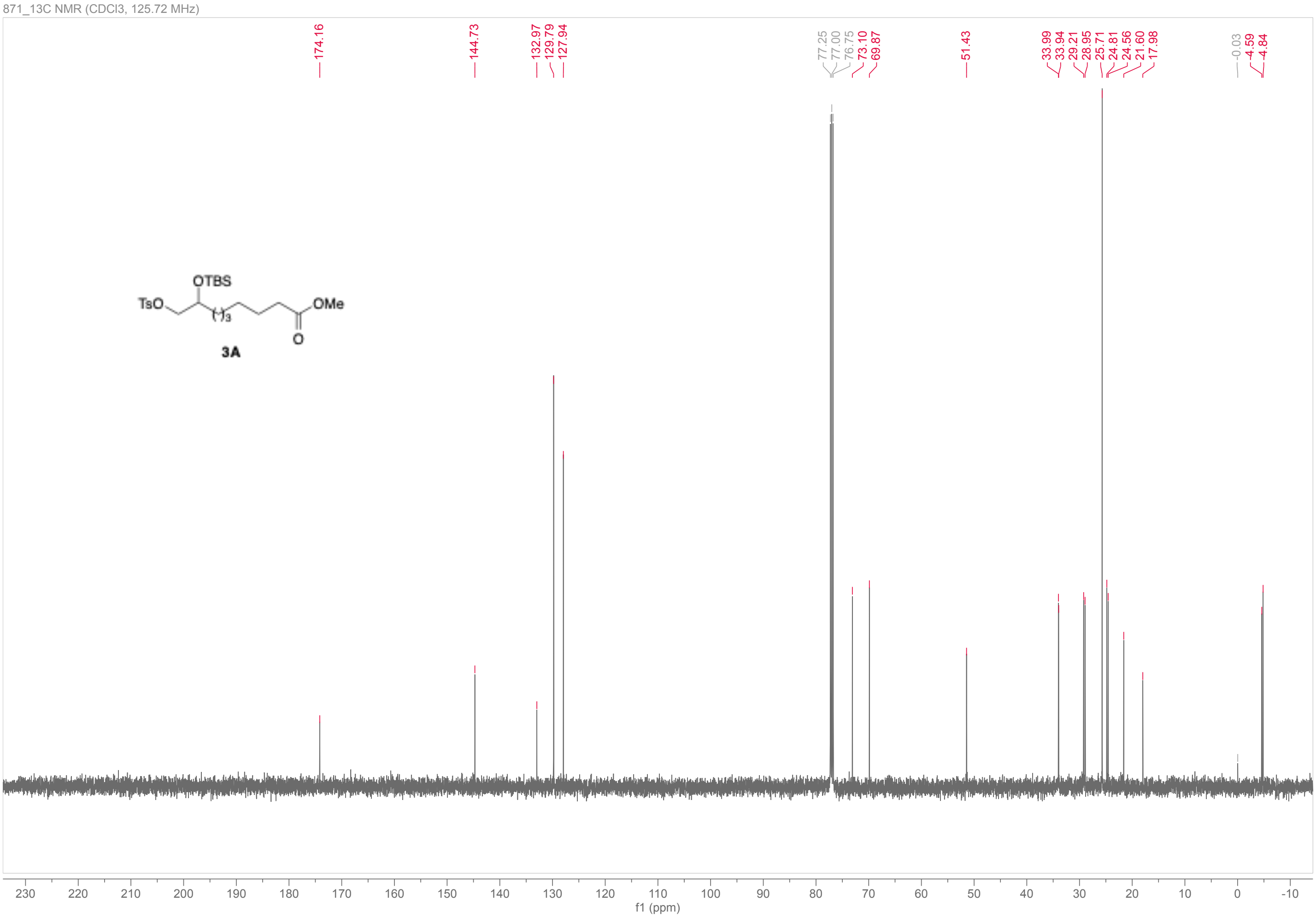

998_1H NMR (CDCl3, 499.94 MHz at 60.0 °C)

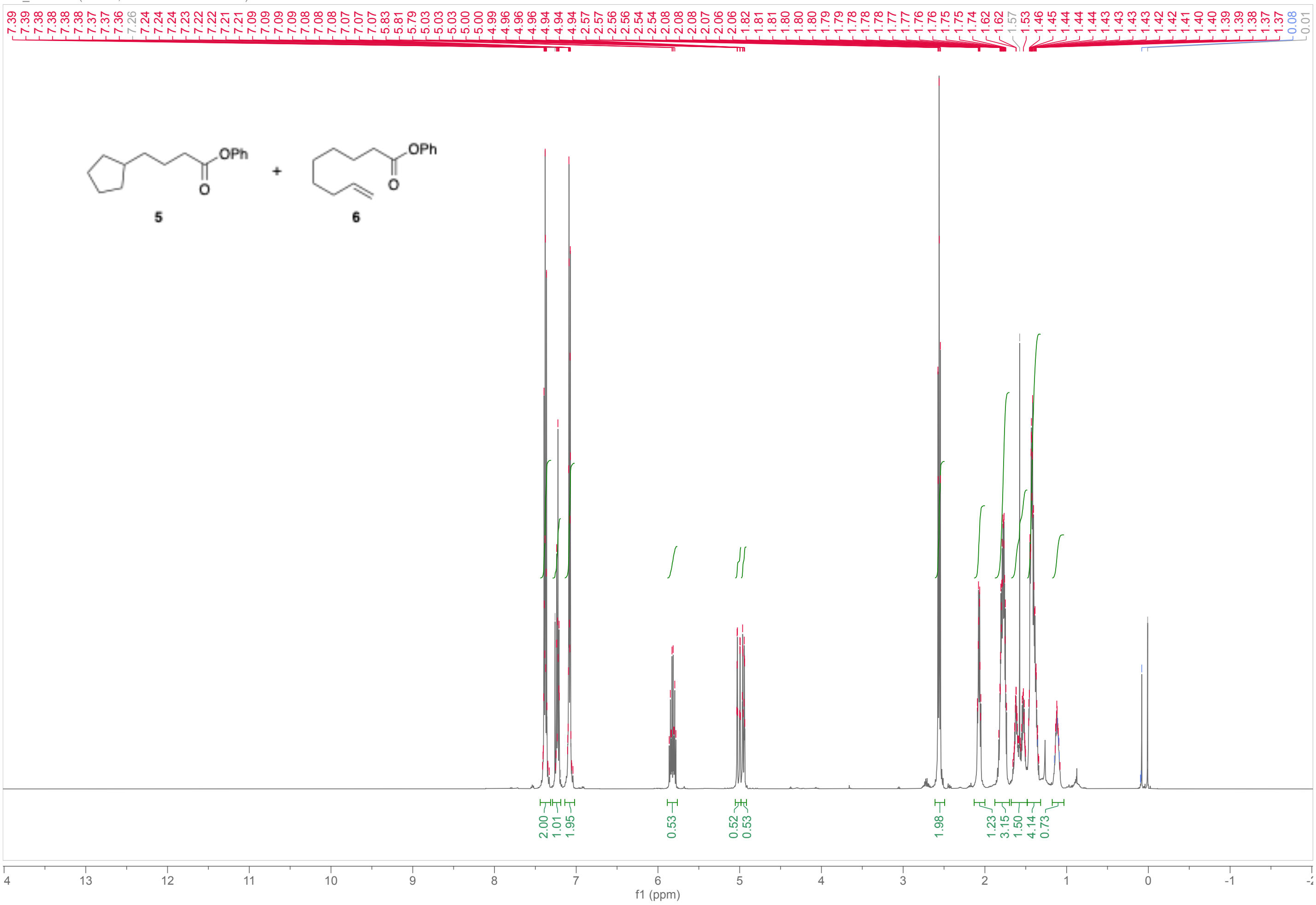

998_13C NMR (CDCl3, 125.72 MHz at 60.0 °C)

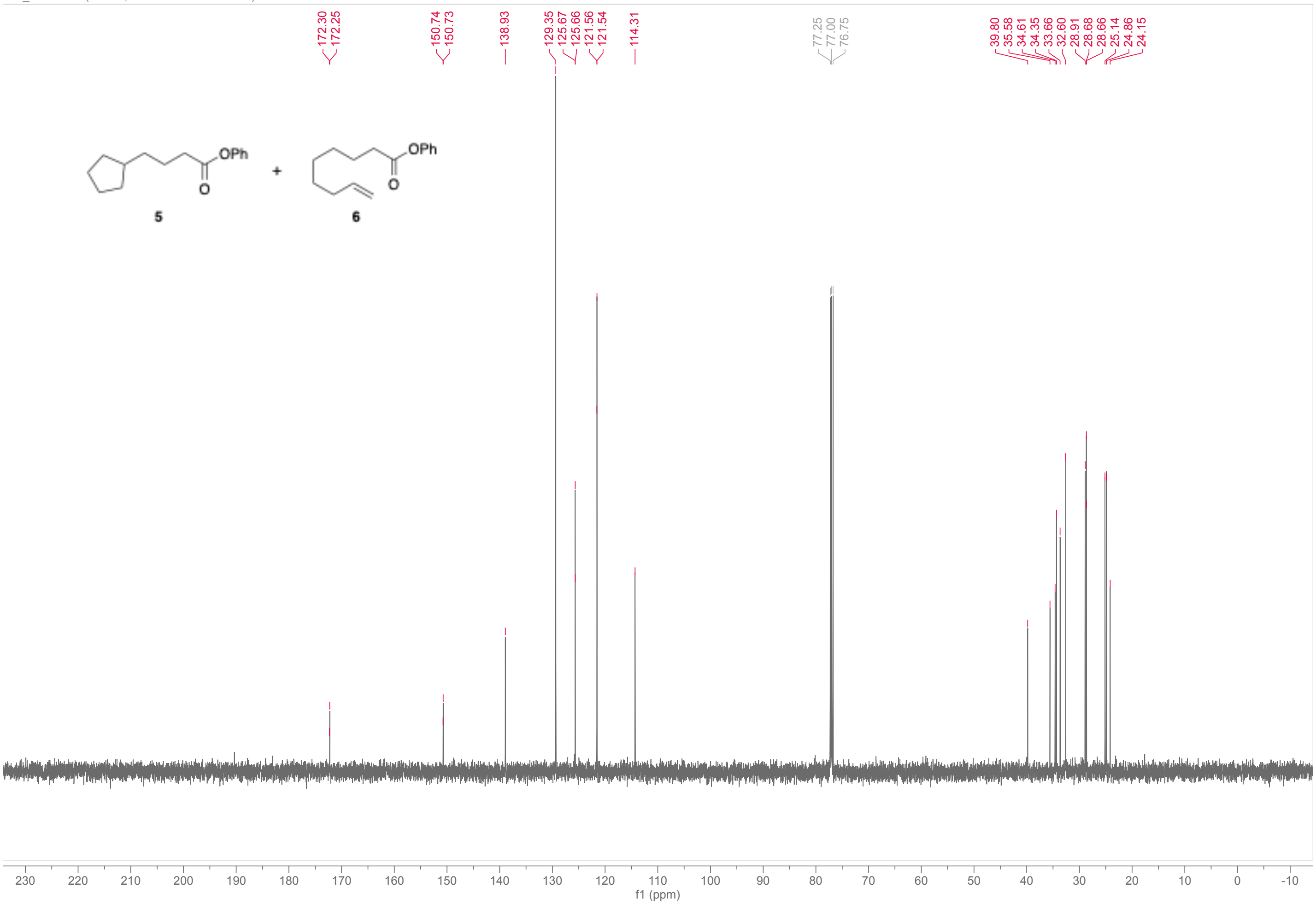

919_1H NMR (CDCl3, 499.94 MHz)

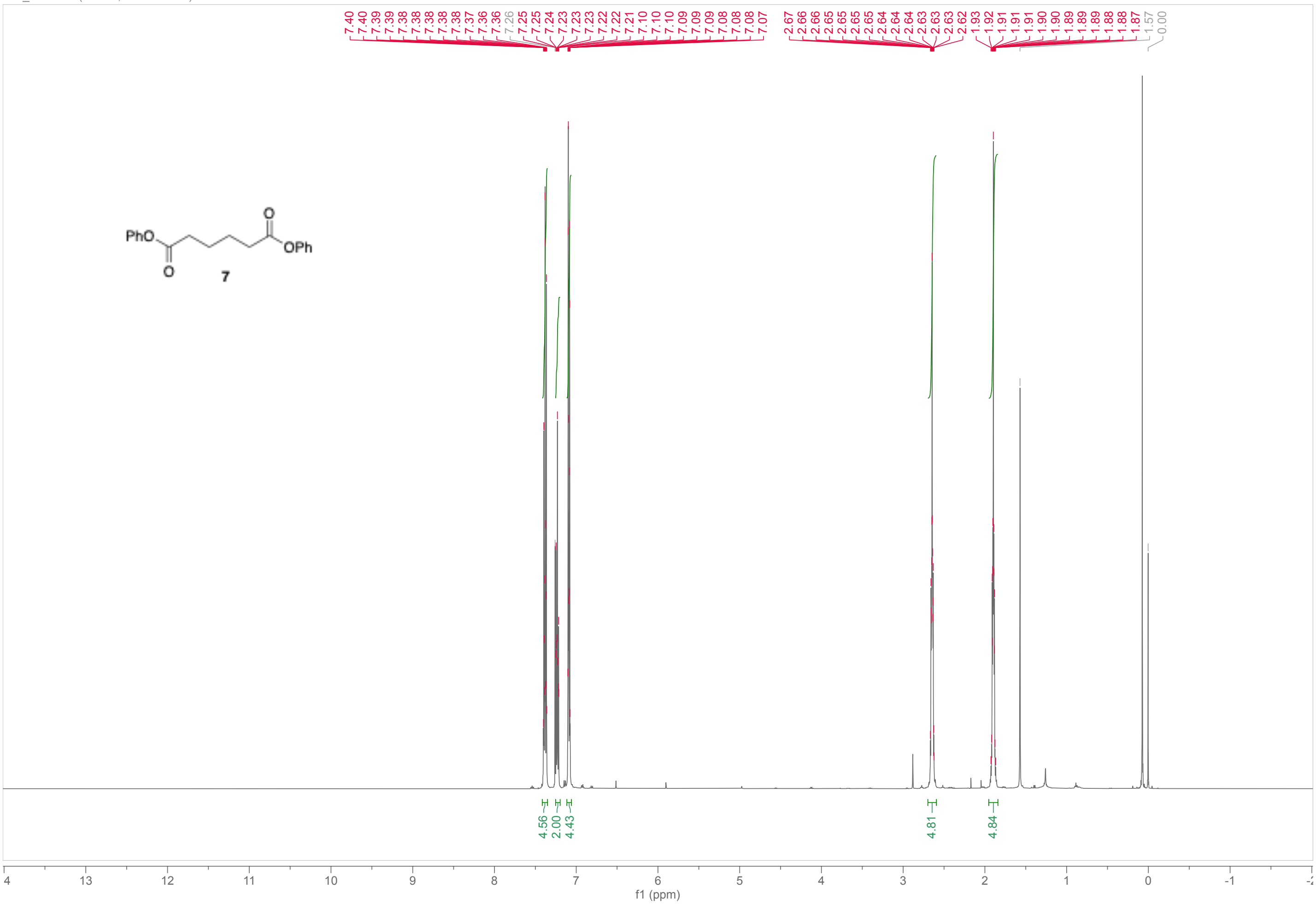

919_13C NMR (CDCl3, 125.72 MHz)

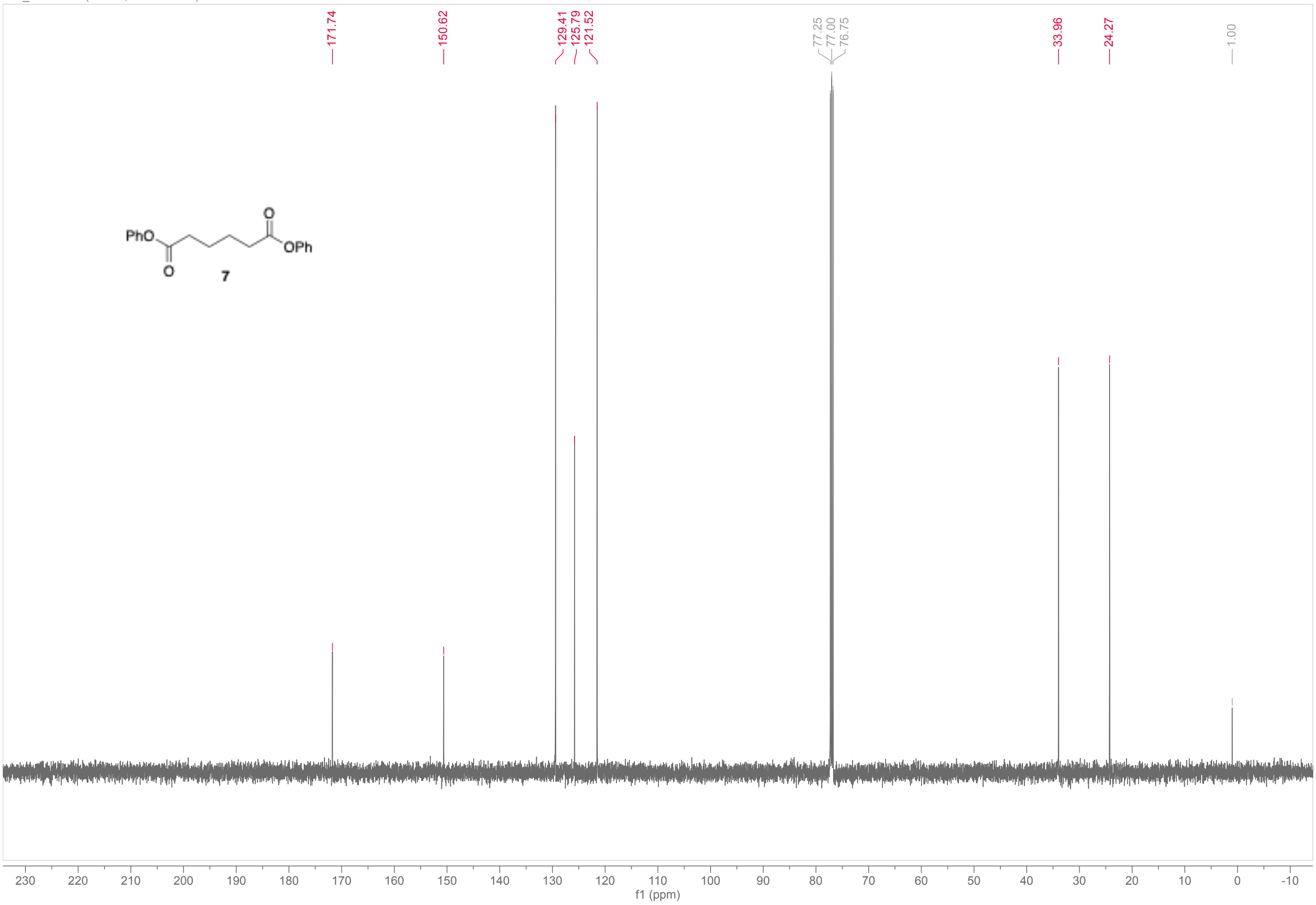

Supplement: RA-011-D0RA10739E-s001 [file RA-011-D0RA10739E-s001.pdf]
